# Advances in mesenchymal stem cell therapy for immune and inflammatory diseases: Use of cell‐free products and human pluripotent stem cell‐derived mesenchymal stem cells

**DOI:** 10.1002/sctm.21-0021

**Published:** 2021-05-19

**Authors:** Li‐Tzu Wang, Ko‐Jiunn Liu, Huey‐Kang Sytwu, Men‐Luh Yen, B. Linju Yen

**Affiliations:** ^1^ Department of Obstetrics & Gynecology National Taiwan University (NTU) Hospital & College of Medicine, NTU Taipei Taiwan Republic of China; ^2^ National Institute of Cancer Research National Health Research Institutes (NHRI) Tainan Taiwan Republic of China; ^3^ National Institute of Infectious Diseases & Vaccinology, NHRI Zhunan Taiwan Republic of China; ^4^ Department & Graduate Institute of Microbiology & Immunology National Defense Medical Center Taipei Taiwan Republic of China; ^5^ Regenerative Medicine Research Group Institute of Cellular & System Medicine, NHRI Zhunan Taiwan Republic of China

**Keywords:** autoimmune diseases, clinical trials, exosomes, extracellular vesicles, graft rejection, human, human pluripotent stem cells, liver cirrhosis, mesenchymal stem/stromal cell therapy, organ transplantation, pulmonary inflammation

## Abstract

Mesenchymal stem cell therapy (MSCT) for immune and inflammatory diseases continues to be popular based on progressive accumulation of preclinical mechanistic evidence. This has led to further expansion in clinical indications from graft rejection, autoimmune diseases, and osteoarthritis, to inflammatory liver and pulmonary diseases including COVID‐19. A clear trend is the shift from using autologous to allogeneic MSCs, which can be immediately available as off‐the‐shelf products. In addition, new products such as cell‐free exosomes and human pluripotent stem cell (hPSC)‐derived MSCs are exciting developments to further prevalent use. Increasing numbers of trials have now published results in which safety of MSCT has been largely demonstrated. While reports of therapeutic endpoints are still emerging, efficacy can be seen for specific indications—including graft‐vs‐host‐disease, strongly Th17‐mediated autoimmune diseases, and osteoarthritis—which are more robustly supported by mechanistic preclinical evidence. In this review, we update and discuss outcomes in current MSCT clinical trials for immune and inflammatory disease, as well as new innovation and emerging trends in the field.

## Graphical abstract

Broad immunomodulatory properties of human tissue and pluripotent stem cell‐derived mesenchymal stem cells (MSCs), as well as MSC‐derived products toward multiple types of immune cells allow for therapeutic use toward a spectrum of immune and inflammatory disorders.

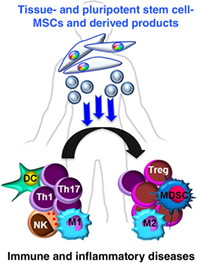

**Significance statement**

Mesenchymal stem cell therapy (MSCT) for immune and inflammatory diseases continues to be popular, leading to further expansion of clinical indications. A clear trend is the shift from using autologous to allogeneic MSCs, and new products such as cell‐free exosomes and human pluripotent stem cell (hPSC)‐derived MSCs, all of which can be immediately available as off‐the‐shelf products. While safety of MSCT is well demonstrated, reports of therapeutic endpoints are still emerging with some trends for specific diseases, which are updated and discussed in this article.

## 1 INTRODUCTION

Mesenchymal stem/stromal cells (MSCs) are multipotent progenitor cells capable of supporting hematopoiesis and differentiation into the multiple mesodermal lineages of osteoblasts, chondrocytes, and adipocytes.^1^, ^2^, ^3^ First found in the bone marrow (BM), MSCs have been isolated from numerous organs/tissues over the past several decades, but in vivo identity remains somewhat elusive, with increasing evidence for a perivascular origin.^4^, ^5^, ^6^ An unexpected function of MSCs—especially prominent with human sources—is its strong immunomodulatory properties, which have been best delineated toward CD4 cells but also well characterized against a variety of myeloid and innate leukocytes, including dendritic cells, monocytes, and macrophages.^7^, ^8^, ^9^ While initial preclinical data of MSC therapeutic efficacy were mainly focused on regenerative and differentiation capacity, it quickly became apparent that the immunomodulatory properties not only are clinically relevant but allow for allogeneic, unmatched use of these progenitor cells. The application of MSCs toward immune and inflammatory diseases rapidly ensued, with a doubling of clinical trials for these conditions within the past 5 years.^10^ Moreover, discoveries of new mechanisms and new products—including using MSC‐derived products and human pluripotent stem cell (hPSC) including embryonic stem cell (ESC) and induced PSC (iPSC)‐derived MSCs—as well as emerging diseases such as COVID‐19 has continued to widen the clinical application of MSC immunomodulation.^11^ We therefore review the current status of clinical trials using MSC therapy (MSCT) for inflammatory or immune‐related diseases and discuss new advances in the field.

## 2 BRIEF SUMMARY ON PRECLINICAL EVIDENCE OF MSC IMMUNOMODULATORY MECHANISMS

**FIGURE 1:**
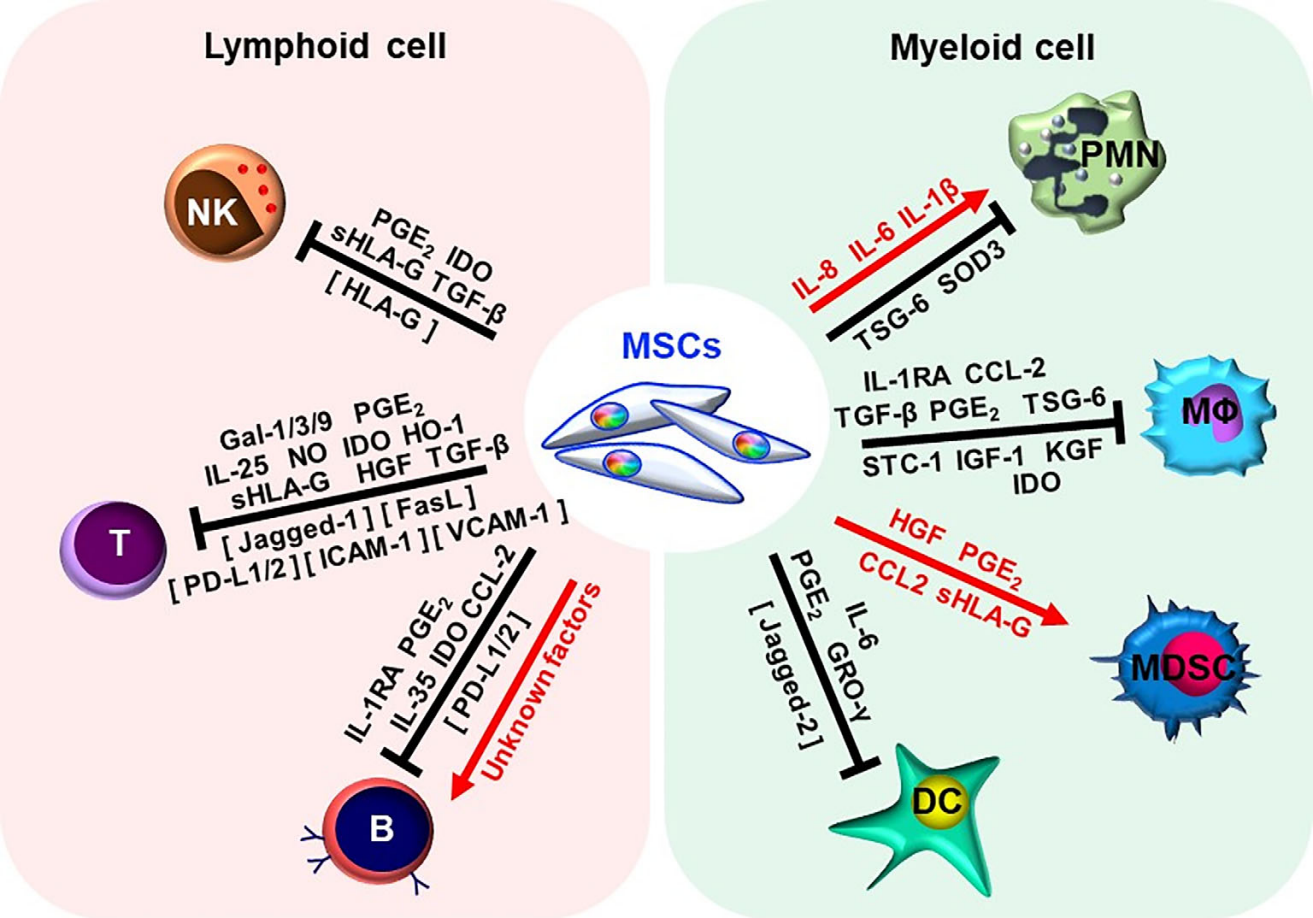
Mechanisms of mesenchymal stem cell (MSC) immunomodulation toward lymphoid and myeloid cells as evidenced in preclinical in vivo studies. NK, natural killer cell; PMN, polymorphonuclear neutrophil; MΦ, macrophage; MDSC, myeloid‐derived suppressor cell; DC, dendritic cell; PGE_2_, prostaglandin E2; IDO, Indoleamine 2,3‐dioxygenase; HLA‐G, human leukocyte antigen‐G; TGF‐β, transforming growth factor beta; Gal, galectin; IL, Interleukin; NO, nitric oxide; HO‐1, heme oxygenase‐1; HGF, hepatocyte growth factor; PD‐L, programmed death‐ligand; IL‐1RA, interleukin‐1 receptor antagonist; CCL2, chemokine ligand 2; TSG‐6, TNF‐stimulated gene 6 protein; SOD3, superoxide dismutase 3; SCT‐1, stanniocalcin‐1; IGF‐1, insulin‐like growth factor‐1; KGF, keratinocyte growth factor; GRO‐γ, growth related oncogene γ. Cell‐contact factors are denoted in brackets

The immunomodulatory properties of MSCs are well demonstrated toward both lymphoid and myeloid cells, with increasing accumulation of mechanistic evidence (Figure 1). MSC immune functions have been best documented against CD4 T lymphocytes, a critical leukocyte population in orchestrating overall immune responses. Numerous reports have shown that MSCs modulate these adaptive cells from an inflammatory milieu filled predominantly with effector T cells to a regulatory T (Treg)‐rich microenvironment largely through paracrine factors, most commonly through transforming growth factor beta (TGF‐β), hepatocyte growth factor (HGF)^12^ prostaglandin E_2_ (PGE_2_),^13^ nitric oxide (NO), and indoleamine 2,3‐dioxygenase (IDO).^8^, ^14^, ^15^, ^16^ While a few studies found cell‐cell contact involved in MSC‐T cell immunomodulation,^17^ this mechanism is more prominent in MSC‐NK interactions, involving downregulation of activating NK receptors such as KIR, NKp30, NKp44 and NKG2D through MSC‐expressed surface and soluble HLA‐G, a non‐classical MHC class I molecule important in fetal‐maternal immunomodulation.^18^, ^19^ Interestingly, immunomodulatory paracrine factors such as PGE_2_ and IDO inducible by inflammatory signals including IFN‐γ and IL‐1β are prominent in MSC interactions across leukocyte subpopulations including all lymphoid cells including T cells, NKs,^20^ and B cells in which IL‐10‐expressing regulatory B cells are expanded.^21^, ^22^ Reports are most scarce for MSC‐B cell interactions, but most demonstrate suppression of B cell proliferation, differentiation, and antibody production^23^, ^24^, ^25^, ^26^, ^27^, ^28^, ^29^; our recent report found that MSC‐B cell interactions may be more complex than previously thought due to MSC source‐specific differences in expression of relevant factors.^30^ Such information on tissue‐specific MSC properties may provide insights which could prove relevant for clinical application.^11^, ^31^

The broad reach of MSC immunomodulation is best exemplified by interactions with myeloid cells, which are much more heterogenous than lymphoid cells. Among early reports of MSC modulation are studies on dendritic cells (DCs)—professional antigen‐presenting cells that initiate T cell response—in which MSC paracrine factors including IL‐6,^32^ PGE_2_,^33^ and growth‐regulated oncogene (GRO)‐γ,^34^ as well as cell‐cell contact through Jagged‐2^35^, suppress maturation and lead to the development of regulatory DCs, which are more immature and tolerogenic. MSCs have also been seen to inhibit activation of macrophages and/or induce polarization into an alternative M2 phenotype which are critical in resolving inflammation, with data indicating involvement of paracrine factors including IL‐1RA,^36^ PGE_2_,^37^ and IDO.^10^, ^38^, ^39^ MSCs also expand myeloid‐derived suppressor cells (MDSCs), a heterogeneous myeloid population defined by their tolerogenic function, through secreting HGF,^40^ CCL2,^41^ PGE2,^42^ and HLA‐G.^43^ MSC interactions with granulocytes are best reported for polymorphonuclear neutrophils (PMNs), but results are somewhat variable possibly due to differences in MSC tissue source. Both BMMSCs and PMSCs prevented PMN apoptosis via IL‐6 secretion,^44^, ^45^ but BMMSCs suppress PMN recruitment via TNF‐stimulated gene 6 protein (TSG‐6) secretion,^46^ and inhibit PMN respiratory burst to prevent neutrophil extracellular traps (NETs) formation^47^; placental MSCs (PMSCs), on the other hand, enhance PMN migration via IL‐8 secretion^45^ and multiple anti‐bacterial functions of PMNs through IL‐1β secretion.^48^ Such accumulation and broadening of evidence for MSC interactions with numerous leukocyte populations is extremely relevant for better tailoring of MSCT—that is, determine which tissue‐specific sources or cell‐derived products to use—for more effective targeting of specific diseases and/or patient subpopulations.

## 3 CURRENT CLINICAL TRIALS OF MSCT FOR IMMUNE‐RELATED DISEASES: OVERVIEW

**FIGURE 2:**
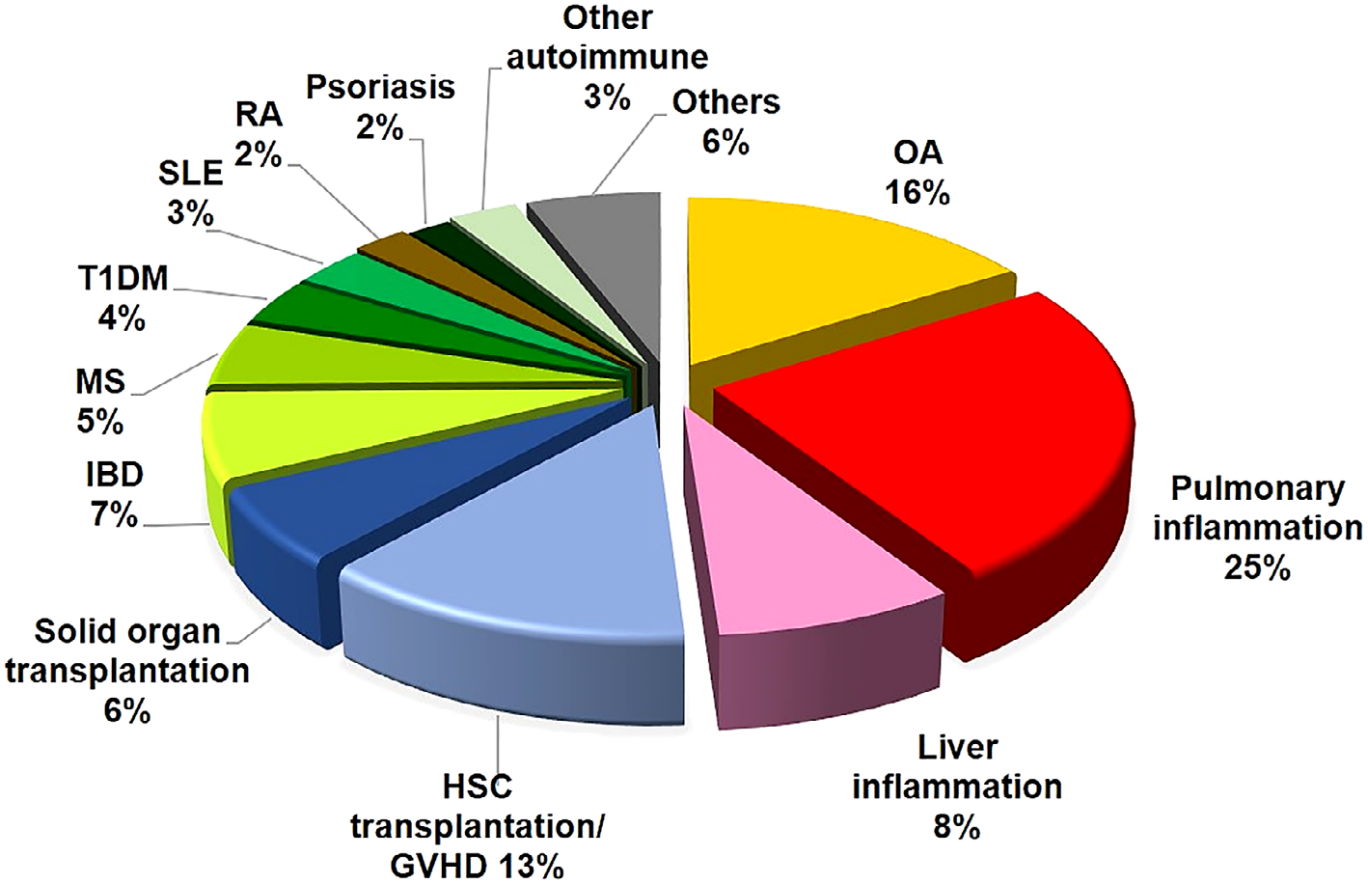
Immune and inflammatory disease indications in current clinical trials of MSC therapy. Summary of clinical trials of immune and inflammatory disease using MSC therapy as registered on the website http://ClinicalTrials.gov (accessed March 2021). OA, osteoarthritis; IBD, inflammatory bowel disease; GVHD, graft vs host disease; MS, multiple sclerosis; T1DM, type 1 diabetes mellitus; SLE, systemic lupus erythematosus; RA, rheumatoid arthritis

**FIGURE 3:**
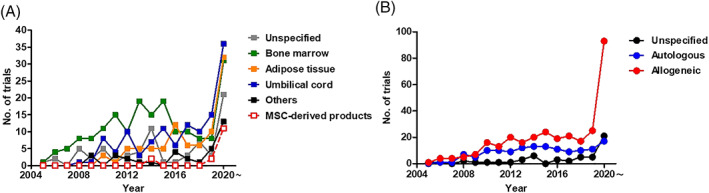
Sources of human MSCs used in clinical trials of immune/inflammatory diseases. A, Number of trials using different tissue sources of human MSCs as well as MSC‐derived products, including exosomes and conditioned medium. B, Number of trials using autologous or allogenic source of human MSCs or MSC‐derived products. Data accessed on March 2021 from the NIH Clinical Trial website (https://ClinicalTrials.gov/)

As of March 2021, there were approximately 1000 clinical trials using MSCT registered on the NIH Clinical Trial Database (https://ClinicalTrials.gov/), with 491 trials (47.1%) for immune−/inflammation‐mediated diseases (Figure 2). The major indications for MSCT trials involving immunomodulatory function include for graft rejection (n = 93), autoimmune diseases (n = 129), and non‐immune diseases with inflammatory components (n = 269). These trials are mainly in early phases, such as Phase 1 to evaluate safety (n = 136 or 27.7%), Phase 2 to evaluate efficacy (n = 97 or 19.8%), or combined Phase 1/2 (n = 203 or 41.3%) (Table 1). Only a few trials are in Phase 3 to determine effectiveness (n = 18 or 3.7%), combined Phase 2/3 studies (n = 15 or 3.1%), and only two trials are in Phase 4 to monitor long‐term effects (0.4%); 20 trials did not specify phase (4.1%). The most prevalent used sources are adult BMMSCs and adipose‐derived MSCs (AdMSCs) at 35.8% and 16.9%, respectively, while the fetal source of Wharton's jelly/umbilical cord (WJUC) at 24.3% is also increasingly popular (Table 1 and Figure 3A). Other sources of MSCs utilized include from fetal sources of umbilical cord blood (UCB), placenta, and amnion tissues, as well as adult sources of menstrual blood (MB), dental pulp (DP), olfactory mucosa (OM), gingiva, and skin. For the first time, there are trials using ESC‐derived MSCs (ESC‐MSCs; n = 2) and iPSC‐derived MSCs (iPSC‐MSCs; n = 2) as well as MSC‐derived products including extracted exosomes or conditioned medium (n = 16). A very clear emerging trend is the use of allogeneic over autologous MSCs in 61.6% vs 27.8%, respectively, while 10.6% of trials did not specify donor source (Figure 3B). In this review, we will focus on immune‐related disease entities with a high number of ongoing clinical trials.

**TABLE 1:** Current clinical trials of MSCT and derived products for immune‐related diseases

MSC source	Total %	Total no.	No. of clinical trial phases						
			N/A	1	1 and 2	2	2 and 3	3	4
Unspecified	13.1	66	2	13	21	20	6^l^	3	1
Bone marrow	35.8	180	7	55^b^	62^c,d,e,f,g^	40^h^	7	9^i^	0
Adipose tissue	16.9	85	3	21	40^j^	16	1	4^i^	0
Umbilical cord	24.5	122	4^a^	33^b^	66^c,d,e,f,k^	17	0	1	1
Umbilical cord blood	2.8	14	1	5	4	2^h^	0	2	0
Placenta	1.0	5	0	1	2^g^	2	0	0	0
Amnion	0.4	2	1^a^	1	0	0	0	0	0
Menstrual blood	0.4	2	0	0	2	0	0	0	0
Dental pulp	0.6	3	0	2	1	0	0	0	0
Olfactory mucosa	0.4	2	0	0	2	0	0	0	0
Gingiva	0.2	1	1	0	0	0	0	0	0
Skin	0.2	1	0	0	1	0	0	0	0
ESC‐MSC	0.4	2	0	1	0	0	0	0	0
iPSC‐MSC^A^	0.4	2	0	1	1	0	0	0	0
MSC‐derived products^B^	3.2	16	2	3	7^j,k^	2	2^l^	0	0
Total no. of clinical trial phases		491	20	136	203	97	15	18	2
Total % of clinical trial phases			4.1	27.7	41.3	19.8	3.1	3.7	0.4

*Notes*: ^a^Trial using two sources of MSCs: WJUC and amnion; ^b,c,d,e,f^Trials using two sources of MSCs: BM and WJUC; ^g^Trial using two sources of MSCs: BM and placenta; ^i^Trial using two sources of MSCs: BM and adipose; ^h^Trial using two sources of MSCs: BM and cord blood; ^j,k,l^Trials using both the MSC and its derived products. ^A^Reprogrammed from peripheral blood mononuclear cells. ^B^Exosomes or trophic factors collected from conditioned medium.

## 4 CURRENT CLINICAL TRIALS OF MSCT FOR IMMUNE‐RELATED DISEASES: SPECIFIC INDICATIONS

### 4.1 Graft‐vs‐host disease and transplant graft rejection

The first clinical report of MSCT for immune‐related diseases was in 2004 for steroid‐resistant severe acute graft‐vs‐host disease (GVHD) in a pediatric patient post‐allogenic hematopoietic stem cell transplantation (HSCT) for leukemia.^49^ This successful case report rapidly led to two large‐scale trials for GVHD^50^ and also rapidly generated interest for use of MSCs in solid organ transplant rejection.^51^, ^52^ A number of preclinical reports support the use of MSCs for improvement of HSCT engraftment and/or GVHD, with mechanisms including suppression of effector T cells and improvement of survival in murine in vivo GVHD models through MSC‐expressed IDO and PD‐L1 in response to inflammatory cytokines such as IFN‐γ, TNF‐α, and IL‐1β, all inflammatory cytokines which are elevated in GVHD patients.^53^, ^54^ MSC modulation of CD4 T cells from effector to regulatory phenotypes was also seen in an early report of solid organ transplant using a murine heart semi‐allograft model,^55^ with rapid accumulation of other studies on MSCs prolonging survival and/or preventing rejection of other organ/tissue types including skin,^56^ pancreatic islet cells,^57^ liver,^58^ kidney,^59^ and cornea.^60^, ^61^, ^62^ A common finding in these studies is the ability of MSCs to promote IL‐10‐producing immune cells, surprisingly in both adaptive as well as innate leukocytes.^55^, ^57^ These early clinical and preclinical successes have made graft rejection a leading indication for use of MSCT.

Currently, there are 63 registered trials of MSCT for HSCT engraftment/GVHD, and 30 trials for prevention or treatment for solid organ transplant rejection (Table S1). The majority of these trials are Phase 1 (n = 22), Phase 2 (n = 23) or combined Phase 1/2 (n = 32), with only a small portion in Phase 3 (n = 6) and combined Phase 2/3 (n = 6); four trials did not specify phase. BM is the major source of MSCs in the trials for GVHD and graft rejection (n = 43), while a few trials utilize MSCs from adipose tissue (n = 8), WJUC (n = 7), UCB (n = 3) with one trial in comparison with BMMSCs; remarkably, there is a very recent trial using iPSC‐MSCs (n = 1). However, 28 trials did not specify the source of MSCs, and two used MSC‐derived products with one using exosomes from WJUCMSCs and one using conditioned medium (CM) from unspecified MSCs. Interestingly, the majority of trials use allogeneic rather than autologous MSCs (57 vs 16 trials, respectively), while one trial used both types and 19 trials were undefined.

Because graft rejection was the first clinical application of MSCT, this field has the most published studies of clinical data so far, with nine reports on GVHD and 11 reports on solid organ transplantation. Several adult and pediatric trials—including two Phase 3 trials—for steroid‐refractory acute or chronic GVHD using intravenous infusion of allogenic BMMSCs at doses of 1~2 × 10^6^/kg showed significant therapeutic efficacy and safety^63^, ^64^, ^65^, ^66^, ^67^, ^68^; moreover, co‐transplantation of BMMSCs at the time of HSCT was found to prevent GVHD progression and/or occurrence.^69^, ^70^ One of the most exciting results is a Phase 1 trial published this year using allogeneic iPSC‐MSCs for steroid‐resistant acute GVHD, in which safety and some efficacy was seen.^71^ Interestingly, for solid organ rejection, all seven published reports used autologous BMMSCs for renal transplantation,^72^, ^73^, ^74^, ^75^, ^76^, ^77^ and pancreatic islet cell transplantation.^78^ Immunosuppressive drugs were used in all these studies except in the islet cell transplantation study and all demonstrated safety, with some efficacy seen in these studies. Allogeneic BMMSCs or WJUCMSC were used in two studies of renal transplantation, and no toxicity was seen.^79^, ^80^ When considering the efficacy of allogenic MSCs, WJUCMSCs was reported to successfully prevent both delayed graft function and acute rejection in renal transplantation,^81^ whereas BMMSCs were not found to induce immunosuppression in a report on liver transplantation.^82^ These published results overall demonstrate that for graft rejection, MSCT is safe and may be efficacious, especially for pediatrics cases of GVHD where allogeneic BMMSC therapy may be particularly beneficial.

### 4.2 Autoimmune diseases

Autoimmune diseases are disorders in which the body's immune system attacks its own cells and organs, and autoreactive T lymphocytes—particularly CD4/helper T cells—are now known to be the critical leukocytes causing these disorders. A key finding in most autoimmune diseases is an imbalance between effector T cell subpopulations of Th1/Th17, vs immunomodulatory IL‐10‐producing Treg, which then lead to inflammation and injury of targeted tissues. Since MSC immunomodulation has been best demonstrated toward CD4 cells, it is no surprise that clinical trials of MSCT for autoimmune diseases collectively are the most numerous. Currently, more than 25% MSCT immune‐related trials are for autoimmune diseases overall, and these 129 trials includes 34 trials for inflammatory bowel disease (IBD), 25 trials for multiple sclerosis (MS), 18 trials for type 1 diabetes mellitus (T1DM), 16 trials for systemic lupus erythematosus (SLE), 12 trials for rheumatoid arthritis (RA), 9 trials for psoriasis, and 15 trials for other autoimmune diseases (Table S2). The overwhelming majority of these trials are in early phases, with 29 in Phase 1, 15 in Phase 2, and 73 in combined Phase1/2; there are seven ongoing trials evaluating efficacy, with three trials in combined Phase 2/3, three trials in Phase 3, and one trial in Phase 4; five trials did not specify phase. Interestingly, all three Phase 3 trials are for IBD—all specifically for Crohn's disease (CD)—and use allogenic sources of BMMSCs (n = 2) or AdMSCs (n = 1). The majority of trials use either BMMSCs (n = 43) or WJUC (n = 39) with two trials using both types of MSCs together, with the next most common source being AdMSCs (n = 21); 15 trials did not unspecified MSC type. There are a few trials using UCBMSCs (n = 4), amniotic MSCs (n = 1), MBMSCs (n = 1), OMMSCs (n = 1), with one trial using BMMSC‐derived neurotrophic factors (BMMSC‐NFs) as well as one using UCBMSC‐derived exosomes. Overwhelmingly, allogenic MSCs are utilized (n = 84) over autologous MSCs (n = 40), with three trials being undefined. Encouragingly, there are two trials comparing different sources of MSCs (autologous BMMSCs vs allogenic WJUCMSCs).

Currently, there are 26 published papers delineating the results of 29 MSCT trials for various autoimmune diseases. The most encouraging results were for CD/IBD, in which peri‐fistula injections of either autologous or allogenic BMMSCs or allogenic AdMSCs promoted healing of perianal fistulas^83^, ^84^, ^85^, ^86^; intravenous injection of allogenic WJUCMSCs to CD and ulcerative colitis (UC) patients also reduced mucosal inflammation.^87^, ^88^ The six published reports using autologous BMMSCs administered intravenously or intrathecally for MS demonstrated safety and some non‐significant reduction of inflammatory parameters^89^, ^90^, ^91^, ^92^, ^93^, ^94^; two other reports utilized allogeneic WJUCMSCs, with one study still ongoing^95^ and one seeing benefit in several clinical parameters.^96^ For RA, both autologous BMMSCs and allogenic AdMSCs were well tolerated and trended toward efficacy.^97^, ^98^ Allogeneic BMMSCs and WJUCMSCs also appear to be safe and feasible for patients with aplastic anemia,^99^ Sjögren syndrome,^100^ and systemic sclerosis.^101^, ^102^ For T1DM, however, while autologous BMMSCs and allogenic WJUCMSCs demonstrated safety and potential therapeutic effect on preserving β‐cell function,^103^, ^104^ allogenic AdMSCs resulted in unanticipated mild transient adverse events in T1DM patients without immunosuppression.^105^ Similarly, in SLE/lupus nephritis, while allogenic BMMSCs were well‐tolerated,^106^ results with WJUCMSCs were discrepant with one trial showing no efficacy^107^ and two trials demonstrate satisfactory clinical response via MSC‐mediated IDO expression in most patients, albeit with disease relapse after 6 months in a few patients.^108^, ^109^ While most of these published clinical reports are early Phase studies focusing on safety, collectively these reports seem to imply that MSCT may be more effective for some autoimmune diseases—IBD and MS—than others. Both IBD and MS are considered to be predominantly mediated by Th17,^110^, ^111^ whereas other commonly MSCT‐targeted autoimmune diseases are more Th1‐predominant, such as T1DM, or prominently involve non‐CD4 populations, such as SLE and RA. Close attention should be paid to trial results for psoriasis, also a predominately Th17 disease, which have been numerous recently. Another variable to follow closely is whether different sources of MSCs would be particularly suited to specific diseases, since preclinical results are starting to indicate tissue‐specific differences in MSC immunomodulatory mechanisms.^30^, ^48^

### 4.3 Osteoarthritis

Due to rapidly aging populations globally, osteoarthritis (OA) has become one of the most common diseases worldwide. While the pathogenesis is degradation of joint cartilage due to wear and tear, this degenerative process elicits inflammation involving macrophage‐mediated activation of innate and adaptive immune responses which lead to further destruction of joint cartilage, a tissue which does not regenerate.^112^ As the stem cells for chondrocytes, MSCs have long been favored for treatment of OA, and the additional benefit of immunomodulation appears to synergistically further improve outcome.^113^, ^114^ Indeed, in addition to MSC chondrogenesis, much preclinical evidence exist on the immunomodulatory efficacy of MSCT for OA, including modulation of activated M1 to alternative M2 macrophage^115^, ^116^ and priming by OA‐related inflammation including IL‐1β—an inflammatory cytokine critical in many joint pathologies—to further enhance MSC immunomodulation.^117^, ^118^

Nearly 18% of all MSCT clinical trials are for OA/non‐rheumatoid degenerative arthritis, and of these 79 trials, most are in early phases with 22 at Phase 1, 15 at Phase 2, and 30 at combined Phase1/2. There are six Phase 3 trials, two are combined Phase 2/3, and four are undefined (Table S3). Most of these trials use AdMSCs (n = 28), BMMSCs (n = 24) and WJUCMSCs (n = 13), with a few trials using MSCs from other tissues, including placenta (n = 1), UCB (n = 4), and DP (n = 1); three trials did not specify source. Interestingly, there are three trials which evaluated two types of MSCs simultaneously, with two trials testing BMMSCs against AdMSCs or PMSCs, and another trial evaluating WJUCMSCs to amniotic MSCs. There are two trials using MSC‐derived products, with one using WJUCMSC‐conditioned medium and the other using AdMSC‐secretome. Surprisingly unlike for other immune diseases, autologous MSCs are most commonly used for OA (n = 43) compared with allogenic MSCs (n = 29); one trial used both autologous and allogeneic sources and six trials were unspecified. Currently, there are 15 published results of MSCT for OA, with five reports each on autologous BMMSCs^119^, ^120^, ^121^, ^122^, ^123^ and autologous AdMSCs.^124^, ^125^, ^126^, ^127^, ^128^ The other five reports all used allogeneic sources, including BMMSCs,^129^, ^130^, ^131^ AdMSCs,^132^ or WJUCMSCs.^133^ All these studies demonstrated safety with intra‐articular MSC injection, as well as varying degrees of improvement in symptoms and disease progression. Such consistent findings highlight MSCT as a promising treatment for OA with its burden on quality of life.

### 4.4 Pulmonary inflammation and COVID‐19

The high entrapment of cells within the lungs after intravenous injection—the most common method to deliver cell therapy—has long been known, and can be taken advantage of in MSCT for pulmonary diseases.^134^ As an organ open to the environment, a number of infectious and non‐infectious/allergic immune pathologies affect the lungs, some with lethal consequences including the post‐inflammatory syndrome associated with COVID‐19.^11^, ^135^ Preclinical data demonstrate that in non‐infectious pulmonary diseases, MSCs can inhibit Th2 responses in asthma, as well as repair alveolar epithelial damage in obstructive diseases such as chronic obstructive pulmonary diseases (COPDs) and restrictive diseases such as idiopathic pulmonary fibrosis (IPF) through secreted factors, exosomes, and even mitochondrial transfer. In infectious pulmonary diseases including pneumonia and acute respiratory distress syndrome (ARDS), preclinical data consistently demonstrate MSCT to be most useful in suppressing over‐exuberate host immune responses. Such reports based on bacterial and influenza pneumonia/ARDS animal models have led to an overwhelming number of MSCT trials for COVID‐19, despite the lack of preclinical evidence with this novel virus.

The numbers of MSCT clinical trials for pulmonary immune‐related diseases have skyrocketed in 2020 due to the COVID‐19 global pandemic, which now stands at 121 registered trials. Over half of the trials are for COVID‐19 (n = 74), and a further 14 trials for pneumonia/ARDS, an inflammatory complication of severe infectious pneumonia including COVID‐19. There are also two trials for asthma, 11 trials for COPD, five trials for IPF, and 15 trials for other inflammatory respiratory diseases including cystic fibrosis and radiation pulmonary injury (Table S4). The trials are predominately in early phase, with 48 in Phase 1, 28 in Phase 2, and 37 in combined Phase 1/2 trials; there is one Phase 3 trial, and three combined Phase 2/3 trials, with four trials in undefined phase. Remarkably, a whopping 92 trials utilize allogeneic MSCs—mainly BM‐ and WJUCMSCs—with only 12 trials using autologous sources and 17 trials undefined. Overall, BMMSCs (n = 39), WJUCMSCs (n = 33), and AdMSCs (n = 17) are the most popular sources, with a few trials using other sources including PMSCs (n = 3), UCBMSCs (n = 1), DPMSCs (n = 2), OMMSCs (n = 1), and even ESC‐MSCs (n = 1) as well as iPSC‐MSCs (n = 1); 13 trials did not specify source. MSC‐derived products are also popular for treatment of pulmonary diseases, with eight trials using cell‐free materials including three trials using AdMSC‐exosomes, one using WJUCMSC‐NFs, one using BMMSC‐exosomes, and three trials using extracellular vesicles (EVs) from unspecified source. Moreover, there were two trials combining MSCs and their derived products with one using WJUCMSCs and CM, and another using an undefined source of MSCs and EVs. The rapid decompensation of many pulmonary infectious diseases including COVID‐19 may be the reason for prevalent use of allogeneic MSCs and cell‐free products, both of which can be immediately available as off‐the‐shelf products.

To date, there are 12 published results demonstrating the feasibility of MSCT in pulmonary immune‐related diseases. There are two Phase 1 reports^136^, ^137^ and two Phase 2 reports—the Acute Physiology and Chronic Health Evaluation III (APACHE III) trial^138^ and the ARDS cue to COVID‐19 (COVID‐AT) trial^139^—as well as one report combining results from one Phase 1 and one Phase 2 trials^140^ demonstrating safety of intravenous infusion of allogenic BMMSCs or AdMSCs for intensive care unit (ICU) patients with moderate to severe ARDS. For COPD‐related lung injury, both intravenous BMMSCs (autologous or allogeneic) and autologous AdMSCs were found to be safe, but therapeutic effects on pulmonary function were found to be variable.^141^, ^142^, ^143^ Infusion of allogenic BMMSCs also well tolerated in patients with IPF or bronchiolitis obliterans syndrome (BOS) after allo‐HSC transplantation,^144^, ^145^ with improvement in one trial for 3‐year survival likely due to a significant increase of IL‐10^+^CD5^+^ B cells in patients receiving MSCT.^145^ For COVID‐19, there are already two published reports on the protocol for MSCT in severely ill patients using allogenic WJUC‐ or DPMSCs^146^, ^147^; additionally, one clinical case series of seven patients—not registered in the NIH clinical trial database—reported safety and efficacy with infusion of (likely) allogeneic BMMSCs.^148^ Clinical results of MSCT for lung diseases—especially COVID‐19—are highly anticipated because of the invaluable information these numerous trials will provide on the efficacy of not only MSCT but also MSC‐related products.

### 4.5 Liver cirrhosis

**FIGURE 4:**
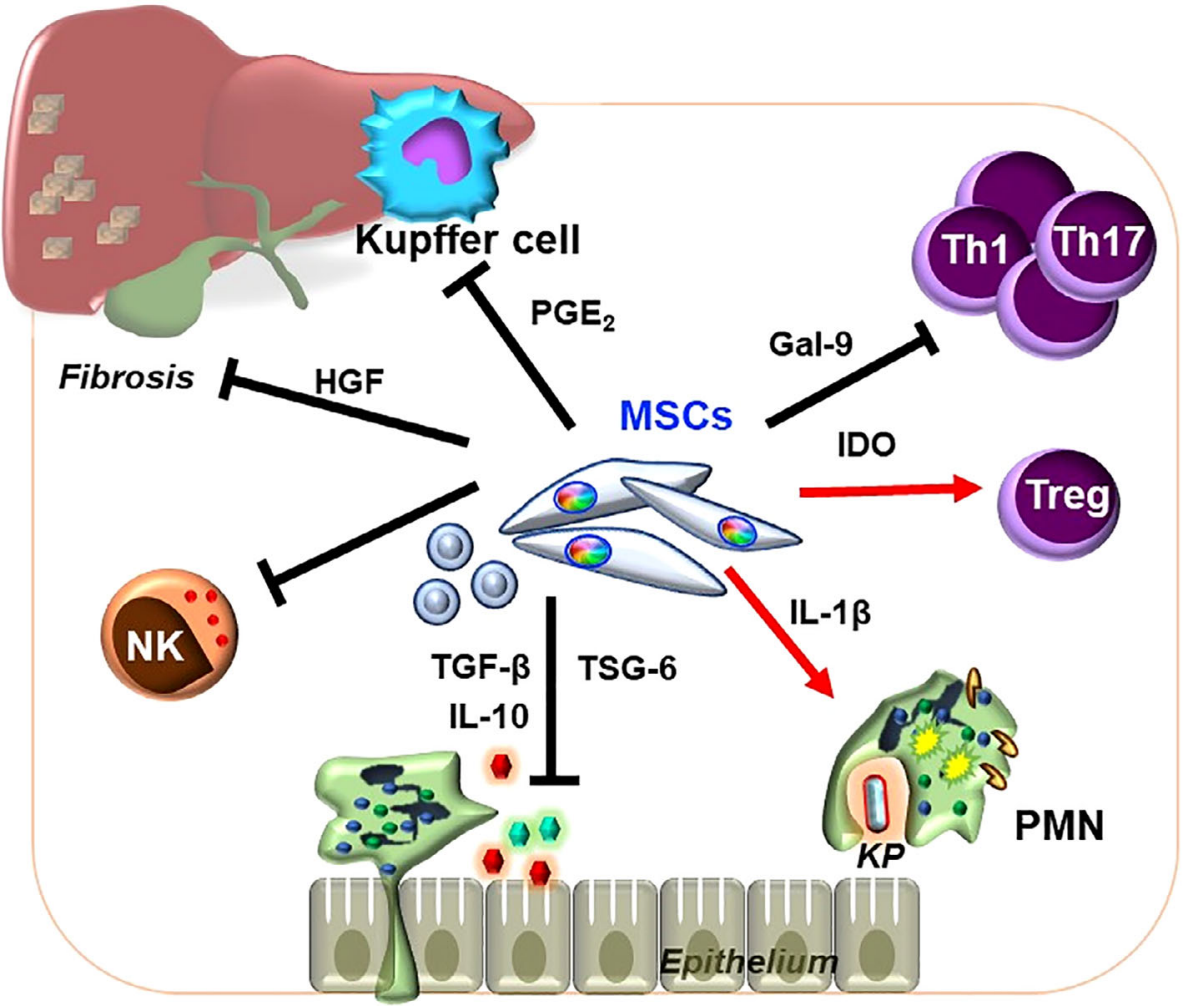
Mechanisms involved in MSCT for hepatitis or liver cirrhosis as evidenced in preclinical in vivo studies. MSC modulation of T cells and liver‐resident MΦs/Kupffer cells to tolerogenic phenotypes and prevention of inflammatory cell recruitment in liver failure, as well as enhancement of anti‐bacterial functions of PMNs during hypervirulent *Klebsiella pneumoniae* (*KP*) infection

Liver fibrosis or cirrhosis is the end‐stage manifestation of many hepatic diseases, ranging from infectious insults due to hepatitis viruses B and C, to non‐infectious conditions such as alcohol abuse and non‐alcoholic fatty liver disease; it is also a risk factor for hepatocellular carcinoma formation.^149^ Compared with other organ systems, mechanisms involved in MSC immunomodulation for liver diseases including cirrhosis are relatively less known (Figure 4). One of the earliest reports in 2004 demonstrated that infusion of murine BMMSCs can reduce chemically‐induced liver fibrosis.^150^ Since then, a number of preclinical in vivo studies have demonstrated that expression of PGE_2_, IDO, and TSG‐6 by BMMSCs can alter the hepatic milieu from being inflammatory to tolerogenic.^151^, ^152^, ^153^, ^154^, ^155^ EVs from human embryonic stem cell (ESC)‐derived MSCs were found to contain anti‐inflammatory cytokines IL‐10 or TGF‐β which ameliorated cirrhosis.^156^ We also recently demonstrated that PMSC treatment in a mouse model of hypervirulent *Klebsiella‐*induced severe intra‐abdominal infection can enhance neutrophil bactericidality to reduce liver injury and increase survival.^48^ In a mouse model of autoimmune cholangitis, liver inflammation was reduced through WJUCMSC‐secreted Gal‐9.^157^ These increasing numbers of preclinical reports support that MSC immunomodulation may be efficacious toward hepatitis and cirrhosis.

Currently, there are 40 registered trials of MSCT for liver failure involving viral hepatitis or cirrhosis, with trials predominantly in early phase: six trials in Phase 1, 12 in Phase 2, and 18 in combined Phase1/2 (Table S5). Only one trial each are in Phase 3 and Phase 4; two trials did not define phase. BM (n = 16) and WJUC (n = 13) are the major sources of MSCs used in these trials, with two other trials using both sources combined. For other sources, there are two trials using AdMSCs, and one trial each using UCBMSCs, MBMSCs, and skin‐derived MSCs (n = 1), as well as four undetermined sources. Again, the majority of trials use allogeneic (n = 21) over autologous sources (n = 15); one trial used both types and three trials were undefined. To date, there are only two published results, with one trial demonstrating therapeutic efficacy of autologous BMMSCs in alcoholic cirrhosis,^158^ while another trial using allogenic WJUCMSCs showed limited improvement on short‐term outcome of HBV‐related liver failure patients.^159^ Further accumulation of clinical data is urgently needed to assess whether MSCT is efficacious for inflammation‐mediated liver failure and/or cirrhosis.

## 5 NEW DEVELOPMENTS: USE OF MSC‐DERIVED PRODUCTS AND HPSC‐MSCS FOR IMMUNE‐RELATED DISEASES

While the minimal reports of adverse events so far in the large number of MSC clinical trials are reassuring, efficacy has been as easy to achieve as was expected. This has increasingly led to the idea that MSC immunomodulation may be represent short‐term immune evasion rather than long‐term immune privilege, and that the MSC itself likely rapidly undergo apoptosis after administration.^160^, ^161^, ^162^ These reports along with increasing evidence of MSC‐derived products harboring immunomodulatory function, render cell‐free options particularly attractive for clinical use. It is well known that paracrine factors are disproportionately responsible for MSC immunomodulatory effects,^7^, ^8^ thus it is perhaps not surprising to find MSC‐derived, cell‐free products to have significant efficacy for immune and inflammatory diseases. Preclinical animal data are promising, demonstrating human MSC‐EVs to be efficacious for GVHD and numerous autoimmune diseases including EAE, T1DM, and RA.^163^ Moreover, in addition to CD4 T cells, MSC‐derived products have been shown to modulate numerous other types of leukocytes including B cells,^164^ NK cells,^165^ macrophages,^166^ and DCs.^167^ To date, there are 16 trials using MSC‐derived products including CM, EVs, or exosomes: three trials are in Phase 1, two in Phase 2, seven are combined Phase 1/2, two are combined Phase 2/3 trials, and two did not specify phase (Table 2). Interestingly, most of the trials (n = 10) are for pulmonary diseases,^11^ with mode of delivery ranging from intravenous injection for severely affected COVID‐19 patients, to aerosol inhalation of AdMSC‐derived exosomes in healthy volunteers and bacterial ARDS patients, and intranasal injection of WJMSC‐derived trophic factors in asthma patients. Other disease indications in which MSC‐derived products are applied include two trials using WJUCMSC‐exosomes or MSC‐CM (unspecified source) for transplant rejection, two trials using WJUCMSC‐exosomes or BMMSC‐derived neurotrophic factors for the autoimmune diseases of T1DM and MS, and two trials using AdMSC‐secretome or WJUCMSC‐CM for OA. The rising popularity of MSC‐free derivatives is also evident in 16 trials for non‐immune diseases which include neural degeneration, wound repair, and ophthalmological conditions (Table S6). It can be anticipated that clinical trials using MSC‐derived products—readily available as off‐the‐shelf products—will continue to increase.

**TABLE 2:** Clinical trials for immune‐related diseases using MSC‐derived products

Condition	NCT number	Title	Status	Delivery	Auto/allo?	Source MSCs	Age	Phase	First posted
Asthma	NCT02192736	Safety and Feasibility Study of Intranasal Mesenchymal Trophic Factor (MTF) for Treatment of Asthma	Active, not recruiting	Intranasal	Allo	WJUCMSC‐TFs	21 to 60 years	Phase 1|Phase 2	July 17, 2014
ARDS	NCT04544215	A Clinical Study of Mesenchymal Progenitor Cell Exosomes Nebulizer for the Treatment of Pulmonary Infection	Recruiting	Aerosol Inhalation	Allo	AdMSC‐Exo	18 to 75 years	Phase 1|Phase 2	September 10, 2020
ARDS	NCT04602104	A Clinical Study of Mesenchymal Stem Cell Exosomes Nebulizer for the Treatment of ARDS	Not yet recruiting	Aerosol Inhalation	Allo	UNS‐MSC‐Exo	18 to 70 years	Phase 1|Phase 2	October 26, 2020
COVID‐19	NCT04276987	A Pilot Clinical Study on Inhalation of Mesenchymal Stem Cells Exosomes Treating Severe Novel Coronavirus Pneumonia	Completed	Aerosol Inhalation	Allo	AdMSC‐Exo	18 to 75 years	Phase 1	February 19, 2020
COVID‐19	NCT04366063	Mesenchymal Stem Cell Therapy for SARS‐CoV‐2‐related Acute Respiratory Distress Syndrome	Recruiting	IV	UNS	UNS‐MSCs/MSC‐EVs	18 to 65 years	Phase 2|Phase 3	April 28, 2020
COVID‐19	NCT04398303	ACT‐20 in Patients With Severe COVID‐19 Pneumonia	Not yet recruiting	IV	Allo	WJUCMSCs/MSC‐CM	18 to 85 years	Phase 1|Phase 2	May 21, 2020
COVID‐19	NCT04657458	Expanded Access Protocol on Bone Marrow Mesenchymal Stem Cell Derived Extracellular Vesicle Infusion Treatment for Patients With COVID‐19 Associated ARDS	Available	IV	UNS	BMMSC‐Evs	18 years and older	Not Applicable	December 8, 2020
COVID‐19	NCT04753476	Treatment of Severe COVID‐19 Patients Using Secretome of Hypoxia‐Mesenchymal Stem Cells in Indonesia	Recruiting	IM	UNS	UNS‐MSC‐secretome	Child, Adult, Older Adult	Phase 2	February 15, 2021
COVID‐19	NCT04798716	The Use of Exosomes for the Treatment of Acute Respiratory Distress Syndrome or Novel Coronavirus Pneumonia Caused by COVID‐19	Not yet recruiting	IV	UNS	UNS‐MSC‐Exo	18 years and older	Phase 1|Phase 2	March 15, 2021
Lung injury test in HD	NCT04313647	A Tolerance Clinical Study on Aerosol Inhalation of Mesenchymal Stem Cells Exosomes In Healthy Volunteers	Recruiting	Aerosol Inhalation	Allo	AdMSC‐Exo	18 to 45 years	Phase 1	March 18, 2020
OA	NCT04314661	Comparative Effectiveness of Arthroscopy and Non‐Arthroscopy Using Mesenchymal Stem Cell Therapy (MSCs) and Conditioned Medium for Osteoartrithis	Recruiting	Intraarticular	Allo	WJUCMSCs/MSC‐CM	55 to 70 years	Phase 1|Phase 2	March 19, 2020
OA	NCT04223622	Effects of ASC Secretome on Human Osteochondral Explants	Not yet recruiting	Unspecified	UNS	AdMSC‐secretome	18 years and older	Not Applicable	January 10, 2020
GVHD with dry eye	NCT04213248	Effect of UMSCs Derived Exosomes on Dry Eye in Patients With cGVHD	Recruiting	On eye	Allo	WJUCMSC‐Exo	18 to 70 years	Phase 1|Phase 2	December 30, 2019
T1DM	NCT02138331	Effect of Microvesicles and Exosomes Therapy on β‐cell Mass in Type I Diabetes Mellitus (T1DM)	Unknown status	IV	Allo	UCBMSC‐Exo	18 to 60 years	Phase 2|Phase 3	May 14, 2014
MS	NCT03799718	Safety and Efficacy of Repeated Administration of NurOwn (MSC‐NTF Cells) in Participants With Progressive MS	Recruiting	Intrathecal	Auto	BMMSC‐NFs	18 to 65 years	Phase 2	January 10, 2019
Skin transplant	NCT04234750	Mesenchymal Stem Cell‐derived Pleiotropic Factor in the Treatment of Donor Sites	Recruiting	On skin	UNS	UNS‐MSC‐CM	6 to 60 years	Phase 1	January 21, 2020

Abbreviations: Ad, adipose; allo, allogenic; Auto, autologous; IV, intravenous; TF, trophic factor; UCB, umbilical cord blood; UNS, unspecified; WJUC, Wharton's jelly/umbilical cord.

The ability to isolate MSCs from a myriad of organs/tissues does not solve the problem that all tissue‐derived MSCs undergo senescence, which not only decreases proliferative capacity but differentiation capacity as well.^168^, ^169^ While there is no data currently demonstrating a loss in MSC immunomodulatory capacity with senescence, the decreased proliferative capacity associated with senescence significantly reduces *ex vivo* cell volumes to the extent that clinical use may no longer be feasible. Thus, there has always been interest in efficient derivation of MSCs from pluripotent stem cells^170^—including ESCs^171^, ^172^, ^173^, ^174^ and more recently, induced pluripotent stem cells (iPSCs)^175^—which express telomerase and provide essentially continuous cell sources. The concerns with using ESCs and iPSCs have always been the fear of teratoma formation due to undifferentiated cells, along with the additional fear of cancer formation in iPSCs with its higher genetic and epigenetic instability.^176^ These concerns can be precluded with stringent QA/QC protocols to exclude undifferentiated and genetically unstable cells.^177^ In addition, the functional variability with primary‐isolated human samples and cell products is avoided since MSCs can be generated from a particular ESC or iPSC line essentially indefinitely to continually provide functionally stable lots of cells and cell products.^178^ While a few reports relying on only one or two hPSC lines did not find immunomodulatory functions,^179^, ^180^ most studies found both ESC‐MSCs and iPSC‐MSCs to be similar to BMMSCs in terms of low immunogenicity and strong immunomodulation toward T cells and NK cells^175^, ^181^, ^182^, ^183^; these findings likely explain the capacity of MSCs to prevent graft rejection since both lymphocyte populations are critically involved in inducing alloreactivity.^184^, ^185^ Very recently, preclinical data on cell‐free products derived from ESC‐/iPSC‐MSCs have emerged as well, with therapeutic immune outcome seen.^156^, ^186^ Currently, there are two trials using ESC‐MSCs for COVID‐19 (combined Phase1/2) and interstitial cystitis (Phase 1) registered in 2020; and two trials using iPSC‐MSCs (CYP‐001) from the same sponsor for GVHD (Phase 1) registered in 2016, and for COVID‐19/ARDS (combined Phase1/2) registered in 2020. Results from the GVHD trial have just been published, in which safety was found with some efficacy seen in this exciting milestone.^71^ Based on increasing preclinical data and these four sentinel trials, it is anticipated that more clinical trials using hPSC‐derived MSCs will be conducted.

## 6 PERSPECTIVES ON CHALLENGES IN MSCT

Although there are over 1000 trials using various types of MSCs or MSC‐free derivatives being conducted in more than 40 countries, only nine MSC‐based products have been approved worldwide for either regenerative or immune‐related diseases.^187^ Encouragingly, these approved MSC‐based products overwhelmingly except for one product are for immune‐related indications, and include allogenic AdMSCs approved by the European Medicines Agency of the European Union to treat complex perianal fistulas in adult CD patients; allogenic BMMSCs approved by Health Canada in Canada, Medsafe in New Zealand, and the Therapeutic Goods Administration in Australia to treat acute GVHD in pediatric patients 2 to 17 years‐old who failed previous therapies for GVHD; and allogenic BMMSCs approved by the Pharmaceuticals and Medical Devices Agency in Japan to treat acute GVHD after allo‐HSC transplantation. In Korea, the Ministry of Food and Drug Safety have authorized three MSC products, including autologous BMMSCs for acute myocardial infarction, allogenic WJUCMSCs for OA, and autologous AdMSCs for CD.

While clinical effectiveness is ultimately the criteria for approval of therapeutic products, the low numbers of approved MSC products also reflect large national/regional differences in regulation for cell‐based products,^188^ as well as difficulties in transitioning from preclinical to clinical platforms.^189^ MSC products in particular suffers from a lack of agreement on robust and relevant characterization criteria for clinical reliability and functionality; the current Minimal Criteria for MSCs date back nearly two decades and was not established for clinical use nor take into account immunomodulatory properties which were largely discovered after these criteria were agreed upon. Moreover, “gold standard” double‐blind randomized clinical trial—including testing for critical parameters such as dose, delivery route, and timing—are clearly more difficult to conduct with complex and live cell‐based products than pharmaceutical products.^178^ Continually progress to overcome these hurdles is occurring which should allow for clinical effectiveness to be more evident in the near future.^187^

## 7 CONCLUSION

MSCT for immune and inflammatory diseases continue to rise in popularity, with a clear trend seen on increasing use of allogeneic sources—especially WJUC and AdMSCs—likely for biological as well as commercial reasons. Approximately 16% of immune‐related trials have now reported some results, most being early phase trials in which safety have largely been demonstrated. While reports of therapeutic endpoints are scarce and still emerging, efficacy have been most consistently reported for specific indications: pediatric cases of GVHD, predominately Th17‐mediated autoimmune diseases such as IBD and MS, and OA in which both the regenerative and immunomodulatory capacity of MSCs can be useful. New developments in use of cell‐free products and iPSC‐MSCs, as well as more preclinical data on tissue‐specific differences in MSC sources are all likely to further improve MSCT outcomes in the very near future.

## CONFLICT OF INTEREST

The authors declared no potential conflicts of interest.

## AUTHOR CONTRIBUTIONS

L.T.W.: conception, manuscript writing, final approval, funding, data research and organization; M.L.Y. and B.L.Y.: conception, manuscript writing, final approval, funding; K.J.L. and H.K.S.: manuscript editing.

## Supporting information

**Appendix S1**: Supplementary Information

## Data Availability

Data sharing is not applicable to this article as no new data were created or analyzed in this study.

## References

[1] Friedenstein AJ. Precursor cells of mechanocytes. Int Rev Cytol. 1976;47:327‐359. 11195 10.1016/s0074-7696(08)60092-3

[2] Dominici M, Le Blanc K, Mueller I, et al. Minimal criteria for defining multipotent mesenchymal stromal cells. The International Society for Cellular Therapy position statement. Cytotherapy. 2006;8:315‐317. 16923606 10.1080/14653240600855905

[3] Reinisch A, Etchart N, Thomas D, et al. Epigenetic and in vivo comparison of diverse MSC sources reveals an endochondral signature for human hematopoietic niche formation. Blood. 2015;125:249‐260. 25406351 10.1182/blood-2014-04-572255 PMC4287636

[4] Sacchetti B, Funari A, Michienzi S, et al. Self‐renewing osteoprogenitors in bone marrow sinusoids can organize a hematopoietic microenvironment. Cell. 2007;131:324‐336. 17956733 10.1016/j.cell.2007.08.025

[5] Crisan M, Yap S, Casteilla L, et al. A perivascular origin for mesenchymal stem cells in multiple human organs. Cell Stem Cell. 2008;3:301‐313. 18786417 10.1016/j.stem.2008.07.003

[6] Nombela‐Arrieta C, Ritz J, Silberstein LE. The elusive nature and function of mesenchymal stem cells. Nat Rev Mol Cell Biol. 2011;12:126‐131. 21253000 10.1038/nrm3049 PMC3346289

[7] Uccelli A, Moretta L, Pistoia V. Mesenchymal stem cells in health and disease. Nat Rev Immunol. 2008;8:726‐736. 19172693 10.1038/nri2395

[8] Chen PM, Yen ML, Liu KJ, et al. Immunomodulatory properties of human adult and fetal multipotent mesenchymal stem cells. J Biomed Sci. 2011;18:49. 21762539 10.1186/1423-0127-18-49 PMC3156728

[9] Le Blanc K, Mougiakakos D. Multipotent mesenchymal stromal cells and the innate immune system. Nat Rev Immunol. 2012;12:383‐396. 22531326 10.1038/nri3209

[10] Wang LT, Ting CH, Yen ML, et al. Human mesenchymal stem cells (MSCs) for treatment towards immune‐ and inflammation‐mediated diseases: review of current clinical trials. J Biomed Sci. 2016;23:76. 27809910 10.1186/s12929-016-0289-5 PMC5095977

[11] Yen BL, Yen ML, Wang LT, et al. Current status of mesenchymal stem cell therapy for immune/inflammatory lung disorders: gleaning insights for possible use in COVID‐19. Stem Cells Translational Medicine. 2020;9:1163‐1173. 32526079 10.1002/sctm.20-0186 PMC7300965

[12] Di Nicola M, Carlo‐Stella C, Magni M, et al. Human bone marrow stromal cells suppress T‐lymphocyte proliferation induced by cellular or nonspecific mitogenic stimuli. Blood. 2002;99:3838‐3843. 11986244 10.1182/blood.v99.10.3838

[13] Aggarwal S, Pittenger MF. Human mesenchymal stem cells modulate allogeneic immune cell responses. Blood. 2005;105:1815‐1822. 15494428 10.1182/blood-2004-04-1559

[14] Meisel R, Zibert A, Laryea M, et al. Human bone marrow stromal cells inhibit allogeneic T‐cell responses by indoleamine 2,3‐dioxygenase‐mediated tryptophan degradation. Blood. 2004;103:4619‐4621. 15001472 10.1182/blood-2003-11-3909

[15] Sato K, Ozaki K, Oh I, et al. Nitric oxide plays a critical role in suppression of T‐cell proliferation by mesenchymal stem cells. Blood. 2007;109:228‐234. 16985180 10.1182/blood-2006-02-002246

[16] Sioud M. New insights into mesenchymal stromal cell‐mediated T‐cell suppression through galectins. Scand J Immunol. 2011;73:79‐84. 21198747 10.1111/j.1365-3083.2010.02491.x

[17] Fan XL, Zhang Y, Li X, et al. Mechanisms underlying the protective effects of mesenchymal stem cell‐based therapy. Cell Mol Life Sci. 2020;77:2771‐2794. 31965214 10.1007/s00018-020-03454-6 PMC7223321

[18] Selmani Z, Naji A, Zidi I, et al. Human leukocyte antigen‐G5 secretion by human mesenchymal stem cells is required to suppress T lymphocyte and natural killer function and to induce CD4+CD25highFOXP3+ regulatory T cells. Stem Cells. 2008;26:212‐222. 17932417 10.1634/stemcells.2007-0554

[19] Liu KJ, Wang CJ, Chang CJ, et al. Surface expression of HLA‐G is involved in mediating immunomodulatory effects of placenta‐derived multipotent cells (PDMCs) towards natural killer lymphocytes. Cell Transplant. 2011;20:1721‐1730. 21669042 10.3727/096368911X580590

[20] Spaggiari GM, Capobianco A, Abdelrazik H, et al. Mesenchymal stem cells inhibit natural killer‐cell proliferation, cytotoxicity, and cytokine production: role of indoleamine 2,3‐dioxygenase and prostaglandin E2. Blood. 2008;111:1327‐1333. 17951526 10.1182/blood-2007-02-074997

[21] Li H, Deng Y, Liang J, et al. Mesenchymal stromal cells attenuate multiple sclerosis via IDO‐dependent increasing the suppressive proportion of CD5+ IL‐10+ B cells. Am J Transl Res. 2019;11:5673‐5688. 31632539 PMC6789281

[22] Chen X, Cai C, Xu D, et al. Human mesenchymal stem cell‐treated regulatory CD23(+)CD43(+) B cells alleviate intestinal inflammation. Theranostics. 2019;9:4633‐4647. 31367246 10.7150/thno.32260 PMC6643430

[23] Che N, Li X, Zhang L, et al. Impaired B cell inhibition by lupus bone marrow mesenchymal stem cells is caused by reduced CCL2 expression. J Immunol. 2014;193:5306‐5314. 25339674 10.4049/jimmunol.1400036 PMC12230989

[24] Luz‐Crawford P, Djouad F, Toupet K, et al. Mesenchymal stem cell‐derived interleukin 1 receptor antagonist promotes macrophage polarization and inhibits B cell differentiation. Stem Cells. 2016;34:483‐492. 26661518 10.1002/stem.2254

[25] Corcione A, Benvenuto F, Ferretti E, et al. Human mesenchymal stem cells modulate B‐cell functions. Blood. 2006;107:367‐372. 16141348 10.1182/blood-2005-07-2657

[26] Che N, Li X, Zhou S, et al. Umbilical cord mesenchymal stem cells suppress B‐cell proliferation and differentiation. Cell Immunol. 2012;274:46‐53. 22414555 10.1016/j.cellimm.2012.02.004

[27] Budoni M, Fierabracci A, Luciano R, et al. The immunosuppressive effect of mesenchymal stromal cells on B lymphocytes is mediated by membrane vesicles. Cell Transplant. 2013;22:369‐379. 23433427 10.3727/096368911X582769

[28] Augello A, Tasso R, Negrini SM, et al. Bone marrow mesenchymal progenitor cells inhibit lymphocyte proliferation by activation of the programmed death 1 pathway. Eur J Immunol. 2005;35:1482‐1490. 15827960 10.1002/eji.200425405

[29] Cho KA, Lee JK, Kim YH, et al. Mesenchymal stem cells ameliorate B‐cell‐mediated immune responses and increase IL‐10‐expressing regulatory B cells in an EBI3‐dependent manner. Cell Mol Immunol. 2017;14:895‐908. 10.1038/cmi.2016.59 PMC5675953 28042143

[30] Lee W, Wang LT, Yen ML, et al. Resident vs nonresident multipotent mesenchymal stromal cell interactions with B lymphocytes result in disparate outcomes. Stem Cells Translational Medicine. 2021;10:711‐724. 33506633 10.1002/sctm.20-0289 PMC8046079

[31] McIntyre LA, Moher D, Fergusson DA, et al. Efficacy of mesenchymal stromal cell therapy for acute lung injury in preclinical animal models: a systematic review. PLoS One. 2016;11:e0147170. 26821255 10.1371/journal.pone.0147170 PMC4731557

[32] Djouad F, Charbonnier LM, Bouffi C, et al. Mesenchymal stem cells inhibit the differentiation of dendritic cells through an interleukin‐6‐dependent mechanism. Stem Cells. 2007;25:2025‐2032. 17510220 10.1634/stemcells.2006-0548

[33] Spaggiari GM, Abdelrazik H, Becchetti F, et al. MSCs inhibit monocyte‐derived DC maturation and function by selectively interfering with the generation of immature DCs: central role of MSC‐derived prostaglandin E2. Blood. 2009;113:6576‐6583. 19398717 10.1182/blood-2009-02-203943

[34] Chen HW, Chen HY, Wang LT, et al. Mesenchymal stem cells tune the development of monocyte‐derived dendritic cells toward a myeloid‐derived suppressive phenotype through growth‐regulated oncogene chemokines. J Immunol. 2013;190:5065‐5077. 23589610 10.4049/jimmunol.1202775

[35] Zhang B, Liu R, Shi D, et al. Mesenchymal stem cells induce mature dendritic cells into a novel Jagged‐2‐dependent regulatory dendritic cell population. Blood. 2009;113:46‐57. 18832657 10.1182/blood-2008-04-154138

[36] Ortiz LA, Dutreil M, Fattman C, et al. Interleukin 1 receptor antagonist mediates the antiinflammatory and antifibrotic effect of mesenchymal stem cells during lung injury. Proc Natl Acad Sci USA. 2007;104:11002‐11007. 17569781 10.1073/pnas.0704421104 PMC1891813

[37] Nemeth K, Leelahavanichkul A, Yuen PS, et al. Bone marrow stromal cells attenuate sepsis via prostaglandin E(2)‐dependent reprogramming of host macrophages to increase their interleukin‐10 production. Nat Med. 2009;15:42‐49. 19098906 10.1038/nm.1905 PMC2706487

[38] FrancoisM, Romieu‐MourezR, LiM, et al. Human MSC suppression correlates with cytokine induction of indoleamine 2,3‐dioxygenase and bystander M2 macrophage differentiation. Mol Ther. 2012;20:187‐195.2193465710.1038/mt.2011.189

[39] Stevens HY, Bowles AC, Yeago C, et al. Molecular crosstalk between macrophages and mesenchymal stromal cells. Front Cell Dev Biol. 2020;8:600160. 33363157 10.3389/fcell.2020.600160 PMC7755599

[40] Yen BL, Yen ML, Hsu PJ, et al. Multipotent human mesenchymal stromal cells mediate expansion of myeloid‐derived suppressor cells via hepatocyte growth factor/c‐met and STAT3. Stem Cell Rep. 2013;1:139‐151. 10.1016/j.stemcr.2013.06.006 PMC3757753 24052949

[41] Lee HJ, Ko JH, Jeong HJ, et al. Mesenchymal stem/stromal cells protect against autoimmunity via CCL2‐dependent recruitment of myeloid‐derived suppressor cells. J Immunol. 2015;194:3634‐3645. 25769927 10.4049/jimmunol.1402139

[42] Lee HJ, Ko JH, Kim HJ, et al. Mesenchymal stromal cells induce distinct myeloid‐derived suppressor cells in inflammation. JCI Insight. 2020;5:e136059. 10.1172/jci.insight.136059 PMC7406289 32453713

[43] Yang S, Wei Y, Sun R, et al. Umbilical cord blood‐derived mesenchymal stromal cells promote myeloid‐derived suppressor cell proliferation by secreting HLA‐G to reduce acute graft‐versus‐host disease after hematopoietic stem cell transplantation. Cytotherapy. 2020;22:718‐733. 32811747 10.1016/j.jcyt.2020.07.008

[44] Raffaghello L, Bianchi G, Bertolotto M, et al. Human mesenchymal stem cells inhibit neutrophil apoptosis: a model for neutrophil preservation in the bone marrow niche. Stem Cells. 2008;26:151‐162. 17932421 10.1634/stemcells.2007-0416

[45] Chen CP, Chen YY, Huang JP, et al. The effect of conditioned medium derived from human placental multipotent mesenchymal stromal cells on neutrophils: possible implications for placental infection. Mol Hum Reprod. 2014;20:1117‐1125. 25140001 10.1093/molehr/gau062

[46] Lee RH, Yu JM, Foskett AM, et al. TSG‐6 as a biomarker to predict efficacy of human mesenchymal stem/progenitor cells (hMSCs) in modulating sterile inflammation in vivo. Proc Natl Acad Sci USA. 2014;111:16766‐16771. 25385603 10.1073/pnas.1416121111 PMC4250139

[47] Jiang D, Muschhammer J, Qi Y, et al. Suppression of neutrophil‐mediated tissue damage‐a novel skill of mesenchymal stem cells. Stem Cells. 2016;34:2393‐2406. 27299700 10.1002/stem.2417 PMC5572139

[48] Wang LT, Wang HH, Chiang HC, et al. Human placental MSC‐secreted IL‐1beta enhances neutrophil bactericidal functions during hypervirulent *Klebsiella* infection. Cell Rep. 2020;32:108188. 32997996 10.1016/j.celrep.2020.108188

[49] Le Blanc K, Rasmusson I, Sundberg B, et al. Treatment of severe acute graft‐versus‐host disease with third party haploidentical mesenchymal stem cells. Lancet. 2004;363:1439‐1441. 15121408 10.1016/S0140-6736(04)16104-7

[50] Dahlke MH, Hoogduijn M, Eggenhofer E, et al. Toward MSC in solid organ transplantation: 2008 position paper of the MISOT study group. Transplantation. 2009;88:614‐619. 19741455 10.1097/TP.0b013e3181b4425a

[51] Taupin P. OTI‐010 osiris therapeutics/JCR pharmaceuticals. Curr Opin Investig Drugs. 2006;7:473‐481. 16729725

[52] Le Blanc K, Frassoni F, Ball L, et al. Mesenchymal stem cells for treatment of steroid‐resistant, severe, acute graft‐versus‐host disease: a phase II study. Lancet. 2008;371:1579‐1586. 18468541 10.1016/S0140-6736(08)60690-X

[53] Kim DS, Jang IK, Lee MW, et al. Enhanced immunosuppressive properties of human mesenchymal stem cells primed by interferon‐gamma. EBioMedicine. 2018;28:261‐273. 29366627 10.1016/j.ebiom.2018.01.002 PMC5898027

[54] Zhang A, Xiong Y, Xu F, et al. IL‐1beta enhances human placenta‐derived mesenchymal stromal cells ability to mediate Th1/Th2 and Th1/CD4(+)IL‐10(+) T cell balance and regulates its adhesion, proliferation and migration via PD‐L1. Cell Immunol. 2020;352:104113. 32331794 10.1016/j.cellimm.2020.104113

[55] Casiraghi F, Azzollini N, Cassis P, et al. Pretransplant infusion of mesenchymal stem cells prolongs the survival of a semiallogeneic heart transplant through the generation of regulatory T cells. J Immunol. 2008;181:3933‐3946. 18768848 10.4049/jimmunol.181.6.3933

[56] Zhang Y, Zhao D, Tian C, et al. Stro‐1‐positive human mesenchymal stem cells prolong skin graft survival in mice. Transplant Proc. 2013;45:726‐729. 23498813 10.1016/j.transproceed.2012.06.086

[57] Kim YH, Wee YM, Choi MY, et al. Interleukin (IL)‐10 induced by CD11b(+) cells and IL‐10‐activated regulatory T cells play a role in immune modulation of mesenchymal stem cells in rat islet allografts. Mol Med. 2011;17:697‐708. 21365122 10.2119/molmed.2010.00098 PMC3146595

[58] Niu J, Yue W, Song Y, et al. Prevention of acute liver allograft rejection by IL‐10‐engineered mesenchymal stem cells. Clin Exp Immunol. 2014;176:473‐484. 24527865 10.1111/cei.12283 PMC4008992

[59] Cao Z, Zhang G, Wang F, et al. Protective effects of mesenchymal stem cells with CXCR4 up‐regulation in a rat renal transplantation model. PLoS One. 2013;8:e82949. 24386129 10.1371/journal.pone.0082949 PMC3875425

[60] Jia Z, Jiao C, Zhao S, et al. Immunomodulatory effects of mesenchymal stem cells in a rat corneal allograft rejection model. Exp Eye Res. 2012;102:44‐49. 22800963 10.1016/j.exer.2012.06.008

[61] Khan MA, Alanazi F, Ahmed HA, et al. iPSC‐derived MSC therapy induces immune tolerance and supports long‐term graft survival in mouse orthotopic tracheal transplants. Stem Cell Res Ther. 2019;10:290. 31547869 10.1186/s13287-019-1397-4 PMC6757436

[62] Vandermeulen M, Erpicum P, Weekers L, et al. Mesenchymal stromal cells in solid organ transplantation. Transplantation. 2020;104:923‐936. 31929427 10.1097/TP.0000000000003077

[63] Perez‐Simon JA, Lopez‐Villar O, Andreu EJ, et al. Mesenchymal stem cells expanded in vitro with human serum for the treatment of acute and chronic graft‐versus‐host disease: results of a phase I/II clinical trial. Haematologica. 2011;96:1072‐1076. 21393326 10.3324/haematol.2010.038356 PMC3128230

[64] Introna M, Lucchini G, Dander E, et al. Treatment of graft versus host disease with mesenchymal stromal cells: a phase I study on 40 adult and pediatric patients. Biol Blood Marrow Transplant. 2014;20:375‐381. 24321746 10.1016/j.bbmt.2013.11.033

[65] Boberg E, von Bahr L, Afram G, et al. Treatment of chronic GvHD with mesenchymal stromal cells induces durable responses: a phase II study. Stem Cells Translational Medicine. 2020;9:1190‐1202. 32573983 10.1002/sctm.20-0099 PMC7519760

[66] Kebriaei P, Hayes J, Daly A, et al. A phase 3 randomized study of Remestemcel‐L versus placebo added to second‐line therapy in patients with steroid‐refractory acute graft‐versus‐host disease. Biol Blood Marrow Transplant. 2020;26:835‐844. 31505228 10.1016/j.bbmt.2019.08.029 PMC7060124

[67] Kurtzberg J, Abdel‐Azim H, Carpenter P, et al. A phase 3, single‐arm, prospective study of Remestemcel‐L, ex vivo culture‐expanded adult human mesenchymal stromal cells for the treatment of pediatric patients who failed to respond to steroid treatment for acute graft‐versus‐host disease. Biol Blood Marrow Transplant. 2020;26:845‐854. 32018062 10.1016/j.bbmt.2020.01.018 PMC8322819

[68] Kurtzberg J, Prockop S, Chaudhury S, et al. Study 275: updated expanded access program for Remestemcel‐L in steroid‐refractory acute graft‐versus‐host disease in children. Biol Blood Marrow Transplant. 2020;26:855‐864. 32044400 10.1016/j.bbmt.2020.01.026 PMC8292970

[69] Baron F, Lechanteur C, Willems E, et al. Cotransplantation of mesenchymal stem cells might prevent death from graft‐versus‐host disease (GVHD) without abrogating graft‐versus‐tumor effects after HLA‐mismatched allogeneic transplantation following nonmyeloablative conditioning. Biol Blood Marrow Transplant. 2010;16:838‐847. 20109568 10.1016/j.bbmt.2010.01.011

[70] Ebens CL, McGrath JA, Tamai K, et al. Bone marrow transplant with post‐transplant cyclophosphamide for recessive dystrophic epidermolysis bullosa expands the related donor pool and permits tolerance of nonhaematopoietic cellular grafts. Br J Dermatol. 2019;181:1238‐1246. 30843184 10.1111/bjd.17858 PMC6731170

[71] Bloor AJC, Patel A, Griffin JE, et al. Production, safety and efficacy of iPSC‐derived mesenchymal stromal cells in acute steroid‐resistant graft versus host disease: a phase I, multicenter, open‐label, dose‐escalation study. Nat Med. 2020;26:1720‐1725. 32929265 10.1038/s41591-020-1050-x

[72] Perico N, Casiraghi F, Introna M, et al. Autologous mesenchymal stromal cells and kidney transplantation: a pilot study of safety and clinical feasibility. Clin J Am Soc Nephrol. 2011;6:412‐422. 20930086 10.2215/CJN.04950610 PMC3052234

[73] Tan J, Wu W, Xu X, et al. Induction therapy with autologous mesenchymal stem cells in living‐related kidney transplants: a randomized controlled trial. Jama. 2012;307:1169‐1177. 22436957 10.1001/jama.2012.316

[74] Perico N, Casiraghi F, Gotti E, et al. Mesenchymal stromal cells and kidney transplantation: pretransplant infusion protects from graft dysfunction while fostering immunoregulation. Transpl Int. 2013;26:867‐878. 23738760 10.1111/tri.12132

[75] Reinders ME, de Fijter JW, Roelofs H, et al. Autologous bone marrow‐derived mesenchymal stromal cells for the treatment of allograft rejection after renal transplantation: results of a phase I study. Stem Cells Translational Medicine. 2013;2:107‐111. 23349326 10.5966/sctm.2012-0114 PMC3659754

[76] Reinders ME, Bank JR, Dreyer GJ, et al. Autologous bone marrow derived mesenchymal stromal cell therapy in combination with everolimus to preserve renal structure and function in renal transplant recipients. J Transl Med. 2014;12:331. 25491391 10.1186/s12967-014-0331-x PMC4273432

[77] Perico N, Casiraghi F, Todeschini M, et al. Long‐term clinical and immunological profile of kidney transplant patients given mesenchymal stromal cell immunotherapy. Front Immunol. 2018;9:1359. 29963053 10.3389/fimmu.2018.01359 PMC6014158

[78] Wang H, Strange C, Nietert PJ, et al. Autologous mesenchymal stem cell and islet cotransplantation: safety and efficacy. Stem Cells Translational Medicine. 2018;7:11‐19. 29159905 10.1002/sctm.17-0139 PMC5746145

[79] Reinders ME, Dreyer GJ, Bank JR, et al. Safety of allogeneic bone marrow derived mesenchymal stromal cell therapy in renal transplant recipients: the neptune study. J Transl Med. 2015;13:344. 26537851 10.1186/s12967-015-0700-0 PMC4632480

[80] Sun Q, Huang Z, Han F, et al. Allogeneic mesenchymal stem cells as induction therapy are safe and feasible in renal allografts: pilot results of a multicenter randomized controlled trial. J Transl Med. 2018;16:52. 29514693 10.1186/s12967-018-1422-x PMC5842532

[81] Sun Q, Hong L, Huang Z, et al. Allogeneic mesenchymal stem cell as induction therapy to prevent both delayed graft function and acute rejection in deceased donor renal transplantation: study protocol for a randomized controlled trial. Trials. 2017;18:545. 29145879 10.1186/s13063-017-2291-y PMC5689202

[82] Detry O, Vandermeulen M, Delbouille MH, et al. Infusion of mesenchymal stromal cells after deceased liver transplantation: a phase I‐II, open‐label, clinical study. J Hepatol. 2017;67:47‐55. 28284916 10.1016/j.jhep.2017.03.001

[83] Molendijk I, Bonsing BA, Roelofs H, et al. Allogeneic bone marrow‐derived mesenchymal stromal cells promote healing of refractory perianal fistulas in patients with Crohn's disease. Gastroenterology. 2015;149:918‐927.e916. 26116801 10.1053/j.gastro.2015.06.014

[84] Panes J, Garcia‐Olmo D, Van Assche G, et al. Expanded allogeneic adipose‐derived mesenchymal stem cells (Cx601) for complex perianal fistulas in Crohn's disease: a phase 3 randomised, double‐blind controlled trial. Lancet. 2016;388:1281‐1290. 27477896 10.1016/S0140-6736(16)31203-X

[85] Dietz AB, Dozois EJ, Fletcher JG, et al. Autologous mesenchymal stem cells, applied in a bioabsorbable matrix, for treatment of perianal fistulas in patients with Crohn's disease. Gastroenterology. 2017;153:59‐62.e52. 28400193 10.1053/j.gastro.2017.04.001 PMC5484717

[86] Panes J, Garcia‐Olmo D, Van Assche G, et al. Long‐term efficacy and safety of stem cell therapy (Cx601) for complex perianal fistulas in patients with Crohn's disease. Gastroenterology. 2018;154:1334‐1342.e1334. 29277560 10.1053/j.gastro.2017.12.020

[87] Forbes GM, Sturm MJ, Leong RW, et al. A phase 2 study of allogeneic mesenchymal stromal cells for luminal Crohn's disease refractory to biologic therapy. Clin Gastroenterol Hepatol. 2014;12:64‐71. 23872668 10.1016/j.cgh.2013.06.021

[88] Hu J, Zhao G, Zhang L, et al. Safety and therapeutic effect of mesenchymal stem cell infusion on moderate to severe ulcerative colitis. Exp Ther Med. 2016;12:2983‐2989. 27882104 10.3892/etm.2016.3724 PMC5103734

[89] Karussis D, Karageorgiou C, Vaknin‐Dembinsky A, et al. Safety and immunological effects of mesenchymal stem cell transplantation in patients with multiple sclerosis and amyotrophic lateral sclerosis. Arch Neurol. 2010;67:1187‐1194. 20937945 10.1001/archneurol.2010.248 PMC3036569

[90] Connick P, Kolappan M, Patani R, et al. The mesenchymal stem cells in multiple sclerosis (MSCIMS) trial protocol and baseline cohort characteristics: an open‐label pre‐test: post‐test study with blinded outcome assessments. Trials. 2011;12:62. 21366911 10.1186/1745-6215-12-62 PMC3059276

[91] Connick P, Kolappan M, Crawley C, et al. Autologous mesenchymal stem cells for the treatment of secondary progressive multiple sclerosis: an open‐label phase 2a proof‐of‐concept study. Lancet Neurol. 2012;11:150‐156. 22236384 10.1016/S1474-4422(11)70305-2 PMC3279697

[92] Llufriu S, Sepulveda M, Blanco Y, et al. Randomized placebo‐controlled phase II trial of autologous mesenchymal stem cells in multiple sclerosis. PLoS One. 2014;9:e113936. 25436769 10.1371/journal.pone.0113936 PMC4250058

[93] Cohen JA, Imrey PB, Planchon SM, et al. Pilot trial of intravenous autologous culture‐expanded mesenchymal stem cell transplantation in multiple sclerosis. Mult Scler. 2018;24:501‐511. 28381130 10.1177/1352458517703802 PMC5623598

[94] Uccelli A, Laroni A, Brundin L, et al. MEsenchymal StEm cells for multiple sclerosis (MESEMS): a randomized, double blind, cross‐over phase I/II clinical trial with autologous mesenchymal stem cells for the therapy of multiple sclerosis. Trials. 2019;20:263. 31072380 10.1186/s13063-019-3346-z PMC6507027

[95] Riordan NH, Morales I, Fernandez G, et al. Clinical feasibility of umbilical cord tissue‐derived mesenchymal stem cells in the treatment of multiple sclerosis. J Transl Med. 2018;16:57. 29523171 10.1186/s12967-018-1433-7 PMC5845260

[96] Alghwiri AA, Jamali F, Aldughmi M, et al. The effect of stem cell therapy and comprehensive physical therapy in motor and non‐motor symptoms in patients with multiple sclerosis: a comparative study. Medicine (Baltimore). 2020;99:e21646. 32846775 10.1097/MD.0000000000021646 PMC7447403

[97] Alvaro‐Gracia JM, Jover JA, Garcia‐Vicuna R, et al. Intravenous administration of expanded allogeneic adipose‐derived mesenchymal stem cells in refractory rheumatoid arthritis (Cx611): results of a multicentre, dose escalation, randomised, single‐blind, placebo‐controlled phase Ib/IIa clinical trial. Ann Rheum Dis. 2017;76:196‐202. 27269294 10.1136/annrheumdis-2015-208918

[98] Shadmanfar S, Labibzadeh N, Emadedin M, et al. Intra‐articular knee implantation of autologous bone marrow‐derived mesenchymal stromal cells in rheumatoid arthritis patients with knee involvement: results of a randomized, triple‐blind, placebo‐controlled phase 1/2 clinical trial. Cytotherapy. 2018;20:499‐506. 29428486 10.1016/j.jcyt.2017.12.009

[99] Cle DV, Santana‐Lemos B, Tellechea MF, et al. Intravenous infusion of allogeneic mesenchymal stromal cells in refractory or relapsed aplastic anemia. Cytotherapy. 2015;17:1696‐1705. 26589752 10.1016/j.jcyt.2015.09.006

[100] Xu J, Wang D, Liu D, et al. Allogeneic mesenchymal stem cell treatment alleviates experimental and clinical Sjogren syndrome. Blood. 2012;120:3142‐3151. 22927248 10.1182/blood-2011-11-391144 PMC3471521

[101] Zhang H, Liang J, Tang X, et al. Sustained benefit from combined plasmapheresis and allogeneic mesenchymal stem cells transplantation therapy in systemic sclerosis. Arthritis Res Ther. 2017;19:165. 28724445 10.1186/s13075-017-1373-2 PMC5518166

[102] van Rhijn‐Brouwer FCC, Gremmels H, Fledderus JO, et al. A randomised placebo‐controlled double‐blind trial to assess the safety of intramuscular administration of allogeneic mesenchymal stromal cells for digital ulcers in systemic sclerosis: the MANUS trial protocol. BMJ Open. 2018;8:e020479. 10.1136/bmjopen-2017-020479 PMC6104757 30127049

[103] Carlsson PO, Schwarcz E, Korsgren O, et al. Preserved beta‐cell function in type 1 diabetes by mesenchymal stromal cells. Diabetes. 2015;64:587‐592. 25204974 10.2337/db14-0656

[104] Cai J, Wu Z, Xu X, et al. Umbilical cord mesenchymal stromal cell with autologous bone marrow cell transplantation in established type 1 diabetes: a pilot randomized controlled open‐label clinical study to assess safety and impact on insulin secretion. Diabetes Care. 2016;39:149‐157. 26628416 10.2337/dc15-0171

[105] Araujo DB, Dantas JR, Silva KR, et al. Allogenic adipose tissue‐derived stromal/stem cells and vitamin D supplementation in patients with recent‐onset type 1 diabetes mellitus: a 3‐month follow‐up pilot study. Front Immunol. 2020;11:993. 32582156 10.3389/fimmu.2020.00993 PMC7280537

[106] Liang J, Zhang H, Kong W, et al. Safety analysis in patients with autoimmune disease receiving allogeneic mesenchymal stem cells infusion: a long‐term retrospective study. Stem Cell Res Ther. 2018;9:312. 30428931 10.1186/s13287-018-1053-4 PMC6236873

[107] Deng D, Zhang P, Guo Y, et al. A randomised double‐blind, placebo‐controlled trial of allogeneic umbilical cord‐derived mesenchymal stem cell for lupus nephritis. Ann Rheum Dis. 2017;76:1436‐1439. 28478399 10.1136/annrheumdis-2017-211073

[108] Wang D, Feng X, Lu L, et al. A CD8 T cell/indoleamine 2,3‐dioxygenase axis is required for mesenchymal stem cell suppression of human systemic lupus erythematosus. Arthritis Rheumatol. 2014;66:2234‐2245. 24756936 10.1002/art.38674 PMC4309486

[109] Wang D, Li J, Zhang Y, et al. Umbilical cord mesenchymal stem cell transplantation in active and refractory systemic lupus erythematosus: a multicenter clinical study. Arthritis Res Ther. 2014;16:R79. 24661633 10.1186/ar4520 PMC4060570

[110] Ueno A, Jeffery L, Kobayashi T, et al. Th17 plasticity and its relevance to inflammatory bowel disease. J Autoimmun. 2018;87:38‐49. 29290521 10.1016/j.jaut.2017.12.004

[111] Moser T, Akgun K, Proschmann U, et al. The role of TH17 cells in multiple sclerosis: therapeutic implications. Autoimmun Rev. 2020;19:102647. 32801039 10.1016/j.autrev.2020.102647

[112] Woodell‐May JE, Sommerfeld SD. Role of inflammation and the immune system in the progression of osteoarthritis. J Orthop Res. 2020;38:253‐257. 31469192 10.1002/jor.24457

[113] Barry F, Murphy M. Mesenchymal stem cells in joint disease and repair. Nat Rev Rheumatol. 2013;9:584‐594. 23881068 10.1038/nrrheum.2013.109

[114] Ruiz M, Maumus M, Fonteneau G, et al. TGFbetai is involved in the chondrogenic differentiation of mesenchymal stem cells and is dysregulated in osteoarthritis. Osteoarthr Cartil. 2019;27:493‐503. 10.1016/j.joca.2018.11.005 30502449

[115] Manferdini C, Maumus M, Gabusi E, et al. Adipose‐derived mesenchymal stem cells exert antiinflammatory effects on chondrocytes and synoviocytes from osteoarthritis patients through prostaglandin E2. Arthritis Rheum. 2013;65:1271‐1281. 23613363 10.1002/art.37908

[116] Manferdini C, Paolella F, Gabusi E, et al. Adipose stromal cells mediated switching of the pro‐inflammatory profile of M1‐like macrophages is facilitated by PGE2: in vitro evaluation. Osteoarthr Cartil. 2017;25:1161‐1171. 10.1016/j.joca.2017.01.011 28153787

[117] van Dalen SCM, Blom AB, Walgreen B, et al. IL‐1beta‐mediated activation of adipose‐derived mesenchymal stromal cells results in PMN reallocation and enhanced phagocytosis: a possible mechanism for the reduction of osteoarthritis pathology. Front Immunol. 2019;10:1075. 31191517 10.3389/fimmu.2019.01075 PMC6545928

[118] Leijs MJ, van Buul GM, Lubberts E, et al. Effect of arthritic synovial fluids on the expression of immunomodulatory factors by mesenchymal stem cells: an explorative in vitro study. Front Immunol. 2012;3:231. 22876244 10.3389/fimmu.2012.00231 PMC3410447

[119] Emadedin M, Ghorbani Liastani M, Fazeli R, et al. Long‐term follow‐up of intra‐articular injection of autologous mesenchymal stem cells in patients with knee, ankle, or hip osteoarthritis. Arch Iran Med. 2015;18:336‐344. 26058927

[120] Lamo‐Espinosa JM, Mora G, Blanco JF, et al. Intra‐articular injection of two different doses of autologous bone marrow mesenchymal stem cells versus hyaluronic acid in the treatment of knee osteoarthritis: multicenter randomized controlled clinical trial (phase I/II). J Transl Med. 2016;14:246. 27565858 10.1186/s12967-016-0998-2 PMC5002157

[121] Al‐Najar M, Khalil H, Al‐Ajlouni J, et al. Intra‐articular injection of expanded autologous bone marrow mesenchymal cells in moderate and severe knee osteoarthritis is safe: a phase I/II study. J Orthop Surg Res. 2017;12:190. 29233163 10.1186/s13018-017-0689-6 PMC5727956

[122] Lamo‐Espinosa JM, Mora G, Blanco JF, et al. Intra‐articular injection of two different doses of autologous bone marrow mesenchymal stem cells versus hyaluronic acid in the treatment of knee osteoarthritis: long‐term follow up of a multicenter randomized controlled clinical trial (phase I/II). J Transl Med. 2018;16:213. 30064455 10.1186/s12967-018-1591-7 PMC6069715

[123] Lamo‐Espinosa JM, Blanco JF, Sanchez M, et al. Phase II multicenter randomized controlled clinical trial on the efficacy of intra‐articular injection of autologous bone marrow mesenchymal stem cells with platelet rich plasma for the treatment of knee osteoarthritis. J Transl Med. 2020;18:356. 32948200 10.1186/s12967-020-02530-6 PMC7501623

[124] Pers YM, Rackwitz L, Ferreira R, et al. Adipose mesenchymal stromal cell‐based therapy for severe osteoarthritis of the knee: a phase I dose‐escalation trial. Stem Cells Translational Medicine. 2016;5:847‐856. 27217345 10.5966/sctm.2015-0245 PMC4922848

[125] Jo CH, Chai JW, Jeong EC, et al. Intra‐articular injection of mesenchymal stem cells for the treatment of osteoarthritis of the knee: a 2‐year follow‐up study. Am J Sports Med. 2017;45:2774‐2783. 28746812 10.1177/0363546517716641

[126] Song Y, Du H, Dai C, et al. Human adipose‐derived mesenchymal stem cells for osteoarthritis: a pilot study with long‐term follow‐up and repeated injections. Regen Med. 2018;13:295‐307. 29417902 10.2217/rme-2017-0152

[127] Lu L, Dai C, Zhang Z, et al. Treatment of knee osteoarthritis with intra‐articular injection of autologous adipose‐derived mesenchymal progenitor cells: a prospective, randomized, double‐blind, active‐controlled, phase IIb clinical trial. Stem Cell Res Ther. 2019;10:143. 31113476 10.1186/s13287-019-1248-3 PMC6528322

[128] Qiao Z, Tang J, Yue B, et al. Human adipose‐derived mesenchymal progenitor cells plus microfracture and hyaluronic acid for cartilage repair: a phase IIa trial. Regen Med. 2020;15:1193‐1214. 32043426 10.2217/rme-2019-0068

[129] Vega A, Martin‐Ferrero MA, Del Canto F, et al. Treatment of knee osteoarthritis with allogeneic bone marrow mesenchymal stem cells: a randomized controlled trial. Transplantation. 2015;99:1681‐1690. 25822648 10.1097/TP.0000000000000678

[130] Gupta PK, Chullikana A, Rengasamy M, et al. Efficacy and safety of adult human bone marrow‐derived, cultured, pooled, allogeneic mesenchymal stromal cells (Stempeucel(R)): preclinical and clinical trial in osteoarthritis of the knee joint. Arthritis Res Ther. 2016;18:301. 27993154 10.1186/s13075-016-1195-7 PMC5168586

[131] Garcia‐Sancho J, Sanchez A, Vega A, et al. Influence of HLA matching on the efficacy of allogeneic mesenchymal stromal cell therapies for osteoarthritis and degenerative disc disease. Transplant Direct. 2017;3:e205. 28894792 10.1097/TXD.0000000000000724 PMC5585421

[132] Zhao X, Ruan J, Tang H, et al. Multi‐compositional MRI evaluation of repair cartilage in knee osteoarthritis with treatment of allogeneic human adipose‐derived mesenchymal progenitor cells. Stem Cell Res Ther. 2019;10:308. 31639063 10.1186/s13287-019-1406-7 PMC6805685

[133] Matas J, Orrego M, Amenabar D, et al. Umbilical cord‐derived mesenchymal stromal cells (MSCs) for knee osteoarthritis: repeated MSC dosing is superior to a single MSC dose and to hyaluronic acid in a controlled randomized phase I/II trial. Stem Cells Translational Medicine. 2019;8:215‐224. 30592390 10.1002/sctm.18-0053 PMC6392367

[134] Baer PC, Sann J, Duecker RP, et al. Tracking of infused mesenchymal stem cells in injured pulmonary tissue in Atm‐deficient mice. Cell. 2020;9:1444. 10.3390/cells9061444 PMC7349119 32531978

[135] Al‐Khawaga S, Abdelalim EM. Potential application of mesenchymal stem cells and their exosomes in lung injury: an emerging therapeutic option for COVID‐19 patients. Stem Cell Res Ther. 2020;11:437. 33059757 10.1186/s13287-020-01963-6 PMC7558244

[136] Zheng G, Huang L, Tong H, et al. Treatment of acute respiratory distress syndrome with allogeneic adipose‐derived mesenchymal stem cells: a randomized, placebo‐controlled pilot study. Respir Res. 2014;15:39. 24708472 10.1186/1465-9921-15-39 PMC3994204

[137] Wilson JG, Liu KD, Zhuo H, et al. Mesenchymal stem (stromal) cells for treatment of ARDS: a phase 1 clinical trial. Lancet Respir Med. 2015;3:24‐32. 25529339 10.1016/S2213-2600(14)70291-7 PMC4297579

[138] Matthay MA, Calfee CS, Zhuo H, et al. Treatment with allogeneic mesenchymal stromal cells for moderate to severe acute respiratory distress syndrome (START study): a randomised phase 2a safety trial. Lancet Respir Med. 2019;7:154‐162. 30455077 10.1016/S2213-2600(18)30418-1 PMC7597675

[139] Payares‐Herrera C, Martinez‐Munoz ME, Vallhonrat IL, et al. Double‐blind, randomized, controlled, trial to assess the efficacy of allogenic mesenchymal stromal cells in patients with acute respiratory distress syndrome due to COVID‐19 (COVID‐AT): a structured summary of a study protocol for a randomised controlled trial. Trials. 2021;22:9. 33407777 10.1186/s13063-020-04964-1 PMC7785778

[140] Liu KD, Wilson JG, Zhuo H, et al. Design and implementation of the START (STem cells for ARDS treatment) trial, a phase 1/2 trial of human mesenchymal stem/stromal cells for the treatment of moderate‐severe acute respiratory distress syndrome. Ann Intensive Care. 2014;4:22. 25593740 10.1186/s13613-014-0022-z PMC4273700

[141] Weiss DJ, Casaburi R, Flannery R, et al. A placebo‐controlled, randomized trial of mesenchymal stem cells in COPD. Chest. 2013;143:1590‐1598. 23172272 10.1378/chest.12-2094 PMC4694112

[142] Stolk J, Broekman W, Mauad T, et al. A phase I study for intravenous autologous mesenchymal stromal cell administration to patients with severe emphysema. QJM. 2016;109:331‐336. 26819296 10.1093/qjmed/hcw001 PMC4888332

[143] Marzouni ET, Dorcheh SP, Nejad‐Moghaddam A. Adipose‐derived mesenchymal stem cells ameliorate lung epithelial injury through mitigating of oxidative stress in mustard lung. Regen Med. 2020;15:1861‐1876. 10.2217/rme-2020-0051 32935623

[144] Glassberg MK, Minkiewicz J, Toonkel RL, et al. Allogeneic human mesenchymal stem cells in patients with idiopathic pulmonary fibrosis via intravenous delivery (AETHER): a phase I safety clinical trial. Chest. 2017;151:971‐981. 27890713 10.1016/j.chest.2016.10.061 PMC6026255

[145] Chen S, Zhao K, Lin R, et al. The efficacy of mesenchymal stem cells in bronchiolitis obliterans syndrome after allogeneic HSCT: a multicenter prospective cohort study. EBioMedicine. 2019;49:213‐222. 31668569 10.1016/j.ebiom.2019.09.039 PMC6945279

[146] Gorman E, Shankar‐Hari M, Hopkins P, et al. Repair of acute respiratory distress syndrome by stromal cell administration in COVID‐19 (REALIST‐COVID‐19): a structured summary of a study protocol for a randomised, controlled trial. Trials. 2020;21:462. 32493473 10.1186/s13063-020-04416-w PMC7267756

[147] Ye Q, Wang H, Xia X, et al. Safety and efficacy assessment of allogeneic human dental pulp stem cells to treat patients with severe COVID‐19: structured summary of a study protocol for a randomized controlled trial (phase I / II). Trials. 2020;21:520. 32532356 10.1186/s13063-020-04380-5 PMC7290137

[148] Leng Z, Zhu R, Hou W, et al. Transplantation of ACE2(−) mesenchymal stem cells improves the outcome of patients with COVID‐19 pneumonia. Aging Dis. 2020;11:216‐228. 32257537 10.14336/AD.2020.0228 PMC7069465

[149] Fattovich G, Stroffolini T, Zagni I, et al. Hepatocellular carcinoma in cirrhosis: incidence and risk factors. Gastroenterology. 2004;127:S35‐S50. 15508101 10.1053/j.gastro.2004.09.014

[150] Fang B, Shi M, Liao L, et al. Systemic infusion of FLK1(+) mesenchymal stem cells ameliorate carbon tetrachloride‐induced liver fibrosis in mice. Transplantation. 2004;78:83‐88. 15257043 10.1097/01.tp.0000128326.95294.14

[151] Milosavljevic N, Gazdic M, Simovic Markovic B, et al. Mesenchymal stem cells attenuate liver fibrosis by suppressing Th17 cells—an experimental study. Transpl Int. 2018;31:102‐115. 28805262 10.1111/tri.13023

[152] Takahashi T, Tibell A, Ljung K, et al. Multipotent mesenchymal stromal cells synergize with costimulation blockade in the inhibition of immune responses and the induction of Foxp3+ regulatory T cells. Stem Cells Translational Medicine. 2014;3:1484‐1494. 25313200 10.5966/sctm.2014-0012 PMC4250203

[153] You Y, Zhang J, Gong J, et al. Mesenchymal stromal cell‐dependent reprogramming of Kupffer cells is mediated by TNF‐alpha and PGE2 and is crucial for liver transplant tolerance. Immunol Res. 2015;62:292‐305. 25982496 10.1007/s12026-015-8660-2

[154] Qu M, Cui J, Zhu J, et al. Bone marrow‐derived mesenchymal stem cells suppress NK cell recruitment and activation in PolyI:C‐induced liver injury. Biochem Biophys Res Commun. 2015;466:173‐179. 26342798 10.1016/j.bbrc.2015.08.125

[155] Wang S, Lee JS, Hyun J, et al. Tumor necrosis factor‐inducible gene 6 promotes liver regeneration in mice with acute liver injury. Stem Cell Res Ther. 2015;6:20. 25890163 10.1186/s13287-015-0019-z PMC4396561

[156] Mardpour S, Hassani SN, Mardpour S, et al. Extracellular vesicles derived from human embryonic stem cell‐MSCs ameliorate cirrhosis in thioacetamide‐induced chronic liver injury. J Cell Physiol. 2018;233:9330‐9344. 29266258 10.1002/jcp.26413

[157] Fan J, Tang X, Wang Q, et al. Mesenchymal stem cells alleviate experimental autoimmune cholangitis through immunosuppression and cytoprotective function mediated by galectin‐9. Stem Cell Res Ther. 2018;9:237. 30223894 10.1186/s13287-018-0979-x PMC6142687

[158] Jang YO, Kim YJ, Baik SK, et al. Histological improvement following administration of autologous bone marrow‐derived mesenchymal stem cells for alcoholic cirrhosis: a pilot study. Liver Int. 2014;34:33‐41. 23782511 10.1111/liv.12218

[159] Xu WX, He HL, Pan SW, et al. Combination treatments of plasma exchange and umbilical cord‐derived mesenchymal stem cell transplantation for patients with hepatitis B virus‐related acute‐on‐chronic liver failure: a clinical trial in China. Stem Cells Int. 2019;2019:4130757. 30863450 10.1155/2019/4130757 PMC6378797

[160] von Bahr L, Batsis I, Moll G, et al. Analysis of tissues following mesenchymal stromal cell therapy in humans indicates limited long‐term engraftment and no ectopic tissue formation. Stem Cells. 2012;30:1575‐1578. 22553154 10.1002/stem.1118

[161] Ankrum JA, Ong JF, Karp JM. Mesenchymal stem cells: immune evasive, not immune privileged. Nat Biotechnol. 2014;32:252‐260. 24561556 10.1038/nbt.2816 PMC4320647

[162] Galleu A, Riffo‐Vasquez Y, Trento C, et al. Apoptosis in mesenchymal stromal cells induces in vivo recipient‐mediated immunomodulation. Sci Transl Med. 2017;9:eaam7828. 29141887 10.1126/scitranslmed.aam7828

[163] Gomzikova MO, James V, Rizvanov AA. Therapeutic application of mesenchymal stem cells derived extracellular vesicles for immunomodulation. Front Immunol. 2019;10:2663. 31849929 10.3389/fimmu.2019.02663 PMC6889906

[164] Khare D, Or R, Resnick I, et al. Mesenchymal stromal cell‐derived exosomes affect mRNA expression and function of B‐lymphocytes. Front Immunol. 2018;9:3053. 30622539 10.3389/fimmu.2018.03053 PMC6308164

[165] Fan Y, Herr F, Vernochet A, et al. Human fetal liver mesenchymal stem cell‐derived exosomes impair natural killer cell function. Stem Cells Dev. 2019;28:44‐55. 30328799 10.1089/scd.2018.0015

[166] Liu H, Liang Z, Wang F, et al. Exosomes from mesenchymal stromal cells reduce murine colonic inflammation via a macrophage‐dependent mechanism. JCI Insight. 2019;4:e131273 10.1172/jci.insight.131273 PMC6975270 31689240

[167] Shahir M, Mahmoud Hashemi S, Asadirad A, et al. Effect of mesenchymal stem cell‐derived exosomes on the induction of mouse tolerogenic dendritic cells. J Cell Physiol. 2020;235:7043‐7055. 32043593 10.1002/jcp.29601 PMC7496360

[168] Wagner W, Horn P, Castoldi M, et al. Replicative senescence of mesenchymal stem cells: a continuous and organized process. PLoS One. 2008;3:e2213. 18493317 10.1371/journal.pone.0002213 PMC2374903

[169] Ho PJ, Yen ML, Tang BC, et al. H2O2 accumulation mediates differentiation capacity alteration, but not proliferative decline, in senescent human fetal mesenchymal stem cells. Antioxid Redox Signal. 2013;18:1895‐1905. 23088254 10.1089/ars.2012.4692 PMC3624695

[170] Karam M, Younis I, Elareer NR, et al. Scalable generation of mesenchymal stem cells and adipocytes from human pluripotent stem cells. Cells. 2020;9:710. 10.3390/cells9030710 PMC7140720 32183164

[171] Barberi T, Willis LM, Socci ND, et al. Derivation of multipotent mesenchymal precursors from human embryonic stem cells. PLoS Med. 2005;2:e161. 15971941 10.1371/journal.pmed.0020161 PMC1160574

[172] Yen ML, Hou CH, Peng KY, et al. Efficient derivation and concise gene expression profiling of human embryonic stem cell‐derived mesenchymal progenitors (EMPs). Cell Transplant. 2011;20:1529‐1545. 21396155 10.3727/096368910X564067

[173] Brown PT, Squire MW, Li WJ. Characterization and evaluation of mesenchymal stem cells derived from human embryonic stem cells and bone marrow. Cell Tissue Res. 2014;358:149‐164. 24927918 10.1007/s00441-014-1926-5 PMC4329984

[174] Lian Q, Lye E, Suan Yeo K, et al. Derivation of clinically compliant MSCs from CD105+, CD24− differentiated human ESCs. Stem Cells. 2007;25:425‐436. 17053208 10.1634/stemcells.2006-0420

[175] Giuliani M, Oudrhiri N, Noman ZM, et al. Human mesenchymal stem cells derived from induced pluripotent stem cells down‐regulate NK‐cell cytolytic machinery. Blood. 2011;118:3254‐3262. 21803852 10.1182/blood-2010-12-325324

[176] Ben‐David U, Benvenisty N. The tumorigenicity of human embryonic and induced pluripotent stem cells. Nat Rev Cancer. 2011;11:268‐277. 21390058 10.1038/nrc3034

[177] Mandai M, Kurimoto Y, Takahashi M. Autologous induced stem‐cell‐derived retinal cells for macular degeneration. N Engl J Med. 2017;377:792‐793. 10.1056/NEJMc1706274 28834478

[178] Temple S, Studer L. Lessons learned from pioneering neural stem cell studies. Stem Cell Rep. 2017;8:191‐193. 10.1016/j.stemcr.2017.01.024 PMC5312267 28199825

[179] Li O, Tormin A, Sundberg B, et al. Human embryonic stem cell‐derived mesenchymal stroma cells (hES‐MSCs) engraft in vivo and support hematopoiesis without suppressing immune function: implications for off‐the shelf ES‐MSC therapies. PLoS One. 2013;8:e55319. 23383153 10.1371/journal.pone.0055319 PMC3558469

[180] Frobel J, Hemeda H, Lenz M, et al. Epigenetic rejuvenation of mesenchymal stromal cells derived from induced pluripotent stem cells. Stem Cell Rep. 2014;3:414‐422. 10.1016/j.stemcr.2014.07.003 PMC4266008 25241740

[181] Trivedi P, Hematti P. Derivation and immunological characterization of mesenchymal stromal cells from human embryonic stem cells. Exp Hematol. 2008;36:350‐359. 18179856 10.1016/j.exphem.2007.10.007 PMC2315792

[182] Yen BL, Chang CJ, Liu KJ, et al. Brief report—human embryonic stem cell‐derived mesenchymal progenitors possess strong immunosuppressive effects toward natural killer cells as well as T lymphocytes. Stem Cells. 2009;27:451‐456. 18988708 10.1634/stemcells.2008-0390

[183] Wang LT, Jiang SS, Ting CH, et al. Differentiation of mesenchymal stem cells from human induced pluripotent stem cells results in downregulation of c‐Myc and DNA replication pathways with immunomodulation toward CD4 and CD8 cells. Stem Cells. 2018;36:903‐914. 29396902 10.1002/stem.2795

[184] Moretta L, Locatelli F, Pende D, et al. Killer Ig‐like receptor‐mediated control of natural killer cell alloreactivity in haploidentical hematopoietic stem cell transplantation. Blood. 2011;117:764‐771. 20889923 10.1182/blood-2010-08-264085

[185] Chong AS, Perkins DL. Transplantation: molecular phenotyping of T‐cell‐mediated rejection. Nat Rev Nephrol. 2014;10:678‐680. 25347945 10.1038/nrneph.2014.197 PMC4425203

[186] Kim H, Lee MJ, Bae EH, et al. Comprehensive molecular profiles of functionally effective MSC‐derived extracellular vesicles in immunomodulation. Mol Ther. 2020;28:1628‐1644. 32380062 10.1016/j.ymthe.2020.04.020 PMC7335740

[187] Lopez‐Beas J, Guadix JA, Clares B, et al. An overview of international regulatory frameworks for mesenchymal stromal cell‐based medicinal products: from laboratory to patient. Med Res Rev. 2020;40:1315‐1334. 32017179 10.1002/med.21659

[188] FDA rejects Mesoblast flagship treatment; 2020. https://www.pharmamanufacturing.com/industrynews/2020/fda-rejects-mesoblast-flagship-treatment/. Accessed Oct 02, 2020.

[189] Lipsitz YY, Timmins NE, Zandstra PW. Quality cell therapy manufacturing by design. Nat Biotechnol. 2016;34:393‐400. 27054995 10.1038/nbt.3525

