# Supplementary material for: Advances in mesenchymal stem cell therapy for immune and inflammatory diseases: Use of cell‐free products and human pluripotent stem cell‐derived mesenchymal stem cells
Source: Stem Cells Transl Med. 2021 May 19;10(9):1288–303. doi: 10.1002/sctm.21-0021 (PMC8380447; doi:10.1002/sctm.21-0021)
Supplement: Supplementary file 1 — Appendix S1: Supplementary Information [file SCT3-10-1288-s001.pdf]

Table S1: Clinical trials for GVHD & transplant graft rejection using MSC-based products

Conditions	NCT Number	Title	Status	Delivery	Auto/ Allo?	Source MSCs	Age	Phases	Enrollment	First Posted	Publications
GVHD	NCT00136903	Safety and Efficacy Study of Adult Human Mesenchymal Stem Cells to Treat Acute Graft Versus Host Disease (GVHD)	Completed	IV	Allo	BMMSCs	18 Years to 70 Years	Phase 2	33	2005/8/29	
GVHD	NCT00284986	Safety and Efficacy of Prochymal for the Salvage of Treatment-Refractory Acute GVHD Patients	Completed	IV	Allo	BMMSCs	6 Months to 70 Years	Phase 2	15	2006/2/1	
GVHD	NCT00366145	Efficacy and Safety of Adult Human Mesenchymal Stem Cells to Treat Steroid Refractory Acute Graft Versus Host Disease	Completed	IV	Allo	BMMSCs	6 Months to 70 Years	Phase 3	260	2006/8/21	Kebriaei 2020[66]
GVHD	NCT00603330	Mesenchymal Stem Cell Infusion as Treatment for Steroid-Resistant Acute Graft Versus Host Disease (GVHD) or Poor Graft Function	Recruiting	IV	UNS	UNS	Child, Adult, Older Adult	Phase 2	100	2008/1/29	
GVHD	NCT00826046	Prochymal® Expanded Access for Adults Who Have Failed Steroid Treatment for Acute Graft Versus Host Disease (GVHD)	No longer available	IV	Allo	BMMSCs	18 Years to 70 Years	Not Applicable		2009/1/21	
GVHD	NCT01589549	Mesenchymal Stromal Cells for Acute Graft Versus Host Disease	Unknown status	IV	UNS	UNS	18 Years to 55 Years	Phase 2	66	2012/5/2	
GVHD	NCT01754454	Safety and Efficacy of UC-MSC in Patients With Acute Severe Graft-versus-host Disease	Unknown status	IV	Allo	WJUCMSCs	18 Years to 70 Years	Phase 1|Phase 2	30	2012/12/21	
GVHD	NCT01765634	Mesenchymal Stem Cells for Treatment of Refractory Acute Graft-versus-host Disease	Unknown status	IV	Allo	BMMSCs	12 Years to 65 Years	Phase 2	40	2013/1/10	
GVHD	NCT01941394	Mesenchymal Stem Cells Infusion for aGVHD Prophylaxis Transplantation	Unknown status	IV	UNS	UNS	18 Years to 60 Years	Phase 2	70	2013/9/13	
GVHD	NCT01956903	Treatment of Refractory Acute Graft-Versus-Host Disease by Sequential Infusion of Allogenic Mesenchymal Stem Cell.	Completed	IV	Allo	UNS	18 Years to 65 Years	Phase 1|Phase 2	15	2013/10/8	
GVHD	NCT02241018	MSCs Combined With CD25 Monoclonal Antibody and Calcineurin Inhibitors for Treatment of Steroid-resistant aGVHD	Unknown status	UNS	Allo	BMMSCs	14 Years to 65 Years	Phase 2|Phase 3	200	2014/9/16	
GVHD	NCT02923375	A Study of CYP-001 for the Treatment of Steroid-Resistant Acute Graft Versus Host Disease	Completed	IV	Allo	iPSC-MSCs	18 Years to 70 Years	Phase 1	16	2016/10/4	Bloor 2020[71]

GVHD	NCT02336230	A Prospective Study of Remestemcel-L, Ex-vivo Cultured Adult Human Mesenchymal Stromal Cells, for the Treatment of Pediatric Patients Who Have Failed to Respond to Steroid Treatment for Acute GVHD	Completed	IV	Allo	BMMSCs	2 Months to 17 Years	Phase 3	55	2015/1/12	Kurtzberg 2020[67]
GVHD	NCT02379442	Early Treatment of Acute Graft Versus Host Disease With Bone Marrow-Derived Mesenchymal Stem Cells and Corticosteroids	Terminated	IV	Allo	BMMSCs	4 Years to 99 Years	Phase 1|Phase 2	1	2015/3/5	
GVHD	NCT02687646	Clinical Trial With MSC for Graft Versus Host Disease Treatment	Recruiting	IV	Allo	AdMSCs	18 Years to 65 Years	Phase 1|Phase 2	15	2016/2/22	
GVHD	NCT03158896	Evaluation of Umbilical Cord-Derived Wharton's Jelly Stem Cells for the Treatment of Acute Graft Versus Host Disease	Active, not recruiting	UNS	Allo	WJUCMSCs	18 Years to 75 Years	Phase 1	10	2017/5/18	
GVHD	NCT04328714	Interferon γ-Primed Mesenchymal Stromal Cells as Prophylaxis for Acute Graft v Host Disease	Not yet recruiting	IV	Allo	BMMSCs	1 Year and older	Phase 1	45	2020/3/31	
GVHD	NCT02770430	Mesenchymal Stem Cells as First Treatment Line for Resistant Acute Graft Versus Host Disease	Unknown status	IV	UNS	BMMSCs	Child, Adult, Older Adult	Phase 2	90	2016/5/12	
GVHD	NCT03631589	MSC for Severe aGVHD	Recruiting	UNS	UNS	UNS	up to 65 Years	Phase 2|Phase 3	50	2018/8/15	
GVHD	NCT03847844	UCMSCs as Front-line Approach of Treatment for Patients With aGVHD	Recruiting	UNS	Allo	WJUCMSCs	16 Years and older	Phase 1|Phase 2	40	2019/2/20	
GVHD	NCT00972660	Safety and Efficacy Study of Allogenic Mesenchymal Stem Cells to Treat Extensive Chronic Graft Versus Host Disease	Unknown status	IV	Allo	UNS	Child, Adult, Older Adult	Phase 2	52	2009/9/7	
GVHD	NCT01222039	Multicenter Clinical Trial for the Evaluation of Mesenchymal Stem Cells From Adipose Tissue in Patients With Chronic Graft Versus Host Disease.	Completed	IV	Allo	AdMSCs	18 Years to 65 Years	Phase 1|Phase 2	19	2010/10/18	
GVHD	NCT01522716	Mesenchymal Stromal Cells as Treatment of Chronic Graft-versus-host Disease	Unknown status	IV	Allo	BMMSCs	18 Years and older	Phase 1	11	2012/2/1	Boberg 2020[65]
GVHD	NCT01526850	Efficacy and Safety Study of Allogenic Mesenchymal Stem Cells for Patients With Chronic Graft Versus Host Disease	Unknown status	BM injection	Allo	UNS	2 Years to 60 Years	Phase 2|Phase 3	100	2012/2/6	
GVHD	NCT01765660	Mesenchymal Stem Cells for Treatment of Refractory Chronic Graft-versus-host Disease	Unknown status	IV	Allo	BMMSCs	12 Years to 65 Years	Phase 2	60	2013/1/10	
GVHD	NCT02291770	Treatment of Chronic Graft-Versus-Host Disease With Mesenchymal Stromal Cells	Unknown status	IV	UNS	UNS	14 Years to 65 Years	Phase 3	130	2014/11/14	

GVHD	NCT04213248	Effect of UMSCs Derived Exosomes on Dry Eye in Patients With cGVHD	Recruiting	On eye	Allo	WJUCMSC-exosome	18 Years to 70 Years	Phase 1|Phase 2	27	2019/12/30	
GVHD	NCT00314483	Evaluation of the Role of Mesenchymal Stem Cells in the Treatment of Graft Versus Host Disease	Unknown status	IV	UNS	UNS	2 Years to 60 Years	Phase 1|Phase 2	25	2006/4/14	
GVHD	NCT00361049	Donor Mesenchymal Stem Cell Infusion in Treating Patients With Acute or Chronic Graft-Versus-Host Disease After Undergoing a Donor Stem Cell Transplant	Completed	IV	Allo	UNS	18 Years and older	Phase 1	49	2006/8/7	
GVHD	NCT00447460	Treatment of Refractory (Acute or Chronic) Graft-Versus-Host Disease by the Infusion of Expanded in-Vitro Allogenic Mesenchymal Stem Cell	Unknown status	IV	Allo	BMMSCs	18 Years to 65 Years	Phase 1|Phase 2	15	2007/3/14	Pérez-Simon 2011[63]
GVHD	NCT00749164	Allogeneic Mesenchymal Stem Cell for Graft-Versus-Host Disease Treatment	Unknown status	IV	Allo	WJUCMSCs	Child, Adult, Older Adult	Phase 1|Phase 2	20	2008/9/9	
GVHD	NCT00827398	Treatment of Steroid Resistant GVHD by Infusion MSC	Completed	IV	UNS	UNS	1 Month to 68 Years	Phase 1|Phase 2	50	2009/1/22	
GVHD	NCT01318330	Safety Study of Homeo-GH (Bone Marrow Derived Clonal Mesenchymal Stem Cell) to Treat Acute/Chronic Graft Versus Host Disease (GVHD)	Completed	IV	Allo	BMMSCs	18 Years and older	Phase 1	10	2011/3/18	
GVHD	NCT01549665	Umbilical Cord Blood-derived Mesenchymal Stem Cells for the Treatment of Steroid-refractory Acute or Chronic Graft-versus-host-disease	Unknown status	IV	Allo	UCBMSCs	up to 30 Years	Phase 1|Phase 2	30	2012/3/9	
GVHD	NCT01764100	Mesenchymal Stromal Cells (MSCs) for the Treatment of Graft Versus Host Disease (GVHD)	Unknown status	IV	Allo	BMMSCs	Child, Adult, Older Adult	Phase 1	10	2013/1/9	Introna 2014[64]
GVHD	NCT02032446	Umbilical Cord Derived Mesenchymal Stromal Cells For The Treatment of Severe Steroid-resistant Graft Versus Host Disease	Recruiting	IV	Allo	WJUCMSCs	up to 70 Years	Phase 1|Phase 2	47	2014/1/10	
GVHD	NCT02055625	Mesenchymal Stem Cells as a Treatment for Oral Complications of Graft-versus-host Disease	Withdrawn	On mucosal lesions	UNS	UNS	18 Years to 80 Years	Phase 1|Phase 2	0	2014/2/5	
GVHD	NCT02359929	BMT Autologous MSCs for GvHD	Recruiting	IV	Auto	BMMSCs	12 Years and older	Phase 1	24	2015/2/10	
GVHD	NCT02824653	Allogenic Bone Marrow Mesenchymal Stem Cells Infusion in Patients With Steroid-refractory GVHD	Completed	IV	Allo	BMMSCs	1 Year to 65 Years	Phase 1|Phase 2	10	2016/7/7	

GVHD	NCT00759018	Prochymal Expanded Access Treatment for Pediatric Patients Who Have Failed Steroids for Acute GVHD	No longer available	IV	Allo	BMMSCs	2 Months to 17 Years	Not Applicable		2008/9/25	Kurtzberg 2020[68]
GVHD	NCT04629833	Treatment Of Steroid-Refractory Acute Graft-versus-host Disease With Mesenchymal Stromal Cells Versus Best Available Therapy	Not yet recruiting	IV	Allo	BMMSCs	12 Years and older	Phase 3	210	2020/11/16	
GVHD	NCT04744116	Addition of Cord Blood Tissue-Derived Mesenchymal Stromal Cells to Ruxolitinib for the Treatment of Steroid-Refractory Acute Graft Versus Host Disease	Not yet recruiting	IV	Allo	UCBMSCs	12 Years to 80 Years	Early Phase 1	24	2021/2/8	
GVHD	NCT04692376	MSC for Treatment of cGVHD After Allo-HSCT	Recruiting	IV	UNS	UNS	18 Years to 65 Years	Phase 2	152	2020/12/31	
BMT	NCT02270307	MSC and Cyclophosphamide for Acute Graft-Versus-Host Disease (aGVHD) Prophylaxis	Unknown status	Unspecified	UNS	UNS	18 Years to 65 Years	Phase 2|Phase 3	40	2014/10/21	
BMT	NCT01061099	Repeated Infusions of Mesenchymal Stromal Cells in Children With Osteogenesis Imperfecta	Completed	IV	Allo	BMMSCs	up to 19 Years	Phase 1	5	2010/2/2	
BMT	NCT02582775	MT2015-20: Biochemical Correction of Severe EB by Allo HSCT and Serial Donor MSCs	Recruiting	IV	Allo	UNS	up to 25 Years	Phase 2	84	2015/10/21	Ebens 2019[70]
HSC transplant	NCT03106662	Mesenchymal Stem Cell Infusion in Haploidentical Hematopoietic Stem Cell Transplantation in Patients With Hematological Malignancies	Completed	IV	UNS	UNS	18 Years and older	Phase 3	6	2017/4/10	
HSC transplant	NCT01045382	MSC and HSC Coinfusion in Mismatched Minitransplants	Recruiting	IV	UNS	UNS	up to 75 Years	Phase 2	120	2010/1/11	
HSC transplant	NCT01092026	Unrelated Umbilical Cord Blood Transplantation With Coinfusion of Mesenchymal Stem Cells	Unknown status	IV	Allo	UNS	15 Years to 60 Years	Phase 1|Phase 2	20	2010/3/24	
HSC transplant	NCT01763086	Mesenchymal Stem Cells for Treatment of Poor Graft Function After Allogeneic Hematopoietic Stem Cell Transplant	Unknown status	IV	Allo	BMMSCs	14 Years to 65 Years	Phase 2	60	2013/1/8	
HSC transplant	NCT01763099	Mesenchymal Stem Cells Combined With Cord Blood for Treatment of Graft Failure	Unknown status	IV	Allo	BMMSCs	14 Years to 65 Years	Phase 2	60	2013/1/8	
HSC transplant	NCT02083718	Peripheral Blood Stem Cell Combined With Mesenchymal Stem Cells for Treatment of Poor Graft Function	Unknown status	IV	Allo	BMMSCs	14 Years to 65 Years	Phase 2	120	2014/3/11	

HSC transplant	NCT02104440	Clinical Trial In The Treatment Of Allogeneic Post-Transplant Cytopenias With Sequential Infusion Of Allogeneic Mesenchymal Cells Expanded In Vitro	Completed	UNS	Allo	UNS	18 Years to 70 Years	Phase 2	15	2014/4/4	
HSC transplant	NCT02181478	Intra-Osseous Co-Transplant of UCB and hMSC	Active, not recruiting	Intraosseous Injection	UNS	UNS	18 Years to 75 Years	Early Phase 1	6	2014/7/4	
HSC transplant	NCT02240992	MSCs With or Without Peripheral Blood Stem Cell for Treatment of Poor Graft Function and Delayed Platelet Engraftment	Unknown status	IV	UNS	UNS	14 Years to 65 Years	Phase 2|Phase 3	120	2014/9/16	
HSC transplant	NCT02247973	Mesenchymal Stem Cells Co-transplantation in Alternative Donor Transplantation of Severe Aplastic Anemia.	Unknown status	IV	Allo	BMMSCs	14 Years to 50 Years	Phase 2	100	2014/9/25	
HSC transplant	NCT03298399	Mesenchymal Stromal Cells for Haplo Hematopoietic Cell Transplantation for Sickle Cell Disease	Withdrawn	IV	Auto	BMMSCs	12 Years to 40 Years	Phase 1	0	2017/10/2	
HSC transplant	NCT03389919	Intraosseous Administration of Mesenchymal Stromal Cells for Patients With Graft Failure After Allo-HSCT	Recruiting	Intraosseous Injection	UNS	UNS	18 Years and older	Phase 3	20	2018/1/4	
HSC transplant	NCT04247945	Co-transplantation of MSC in the Setting of Allo-HSCT	Recruiting	IV	UNS	UNS	up to 65 Years	Phase 2|Phase 3	120	2020/1/30	
HSC transplant	NCT01033552	Biochemical Correction of Severe EB by Allo HSCT and "Off-the-shelf" MSCs	Recruiting	IV	Allo	BMMSCs/UCBMSCs	up to 25 Years	Phase 2	75	2009/12/16	
HSC transplant	NCT02407470	Safety and Efficacy of Patient's Own AD-MSC and AD-HSC Transplantation in Patients With Severe Aplastic Anemia	Unknown status	IV	Auto	AdMSCs	14 Years to 70 Years	Phase 1|Phase 2	90	2015/4/3	
HSC transplant	NCT00504803	Mesenchymal Stem Cell Infusion as Prevention for Graft Rejection and Graft-versus-host Disease	Completed	IV	Allo	BMMSCs	up to 75 Years	Phase 2	30	2007/7/20	Baron 2010[69]
HSC transplant	NCT00823316	Safety and Efficacy Study of Umbilical Cord Blood-Drived Mesenchymal Stem Cells to Promote Engraftment of Unrelated Hematopoietic Stem Cell Transplantation	Completed	IV	Allo	UCBMSCs	2 Years to 19 Years	Phase 1|Phase 2	10	2009/1/15	
Heart transplant	NCT02472002	Mesenchymal Stem Cell Administration in the Treatment of Coronary Graft Disease in Heart Transplant Patients	Suspended	Intramyocardial injection	Auto	BMMSCs	18 Years to 80 Years	Phase 1|Phase 2	14	2015/6/15	
Islet transplant	NCT02384018	Mesenchymal Stem Cell and Islet Co-transplantation	Active, not recruiting	UNS	Auto	BMMSCs	18 Years to 69 Years	Phase 1	24	2015/3/10	Wang 2018[78]

Islet transplant	NCT00646724	Cotransplantation of Islet and Mesenchymal Stem Cell in Type 1 Diabetic Patients	Unknown status	UNS	Auto	UNS	18 Years to 60 Years	Phase 1|Phase 2	30	2008/3/28	
Kideney transplant	NCT02492308	Induction With SVF Derived MSC in Living-related Kidney Transplantation	Unknown status	IV	Auto	AdMSCs	18 Years to 60 Years	Phase 1|Phase 2	120	2015/7/8	
Kideney transplant	NCT00658073	Induction Therapy With Autologous Mesenchymal Stem Cells for Kidney Allografts	Completed	IV	Auto	BMMSCs	18 Years to 60 Years	Not Applicable	165	2008/4/14	Tan 2012[73]
Kideney transplant	NCT00659620	Mesenchymal Stem Cell Transplantation in the Treatment of Chronic Allograft Nephropathy	Unknown status	IV	UNS	UNS	18 Years to 60 Years	Phase 1|Phase 2	20	2008/4/16	
Kideney transplant	NCT00734396	Mesenchymal Stem Cells and Subclinical Rejection	Completed	IV	Auto	BMMSCs	18 Years to 65 Years	Phase 1|Phase 2	15	2008/8/14	Reinders 2013[75]
Kideney transplant	NCT00752479	Mesenchymal Stem Cells Under Basiliximab/Low Dose RATG to Induce Renal Transplant Tolerance	Terminated	IV	Auto	BMMSCs	18 Years to 80 Years	Phase 1|Phase 2	4	2008/9/15	Perico 2011[72], Perico 2013[74], Perico 2018[77]
Kideney transplant	NCT02012153	Mesenchymal Stromal Cells in Kidney Transplant Recipients	Recruiting	IV	Auto	BMMSCs	18 Years and older	Phase 1	6	2013/12/16	Perico 2018[77]
Kideney transplant	NCT02057965	Mesenchymal Stromal Cell Therapy in Renal Recipients	Recruiting	IV	Auto	BMMSCs	18 Years to 75 Years	Phase 2	70	2014/2/7	Reinders 2014[76]
Kideney transplant	NCT02387151	Allogeneic Mesenchymal Stromal Cell Therapy in Renal Transplant Recipients	Completed	IV	Allo	BMMSCs	18 Years to 75 Years	Phase 1	10	2015/3/12	Reinders 2015[79]
Kideney transplant	NCT02409940	To Elucidate the Effect of Mesenchymal Stem Cells on the T Cell Repertoire of the Kidney Transplant Patients	Unknown status	IV	Auto/ Allo	BMMSCs	18 Years to 65 Years	Phase 1	17	2015/4/7	
Kideney transplant	NCT02490020	A Perspective Multicenter Controlled Study On Application Of Mesenchymal Stem Cell(MSC) To Prevent Rejection After Renal Transplantation By Donation After Cardiac Death	Unknown status	IV	Allo	WJUCMSCs	18 Years to 60 Years	Phase 1	260	2015/7/3	Sun 2018[80], Sun 2017[81]
Kideney transplant	NCT02492490	Effect of SVF Derived MSC in DCD Renal Transplantation	Unknown status	IV	Auto	AdMSCs	18 Years to 60 Years	Phase 1|Phase 2	120	2015/7/8	
Kideney transplant	NCT02561767	Effect of BM-MSCs in DCD Kidney Transplantation	Unknown status	IV	Allo	BMMSCs	18 Years to 65 Years	Phase 1|Phase 2	120	2015/9/28	
Kideney transplant	NCT02563340	Effect of BM-MSCs on Chronic AMR After Kidney Transplantation	Unknown status	IV	Allo	BMMSCs	18 Years to 65 Years	Phase 1|Phase 2	60	2015/9/30	

Kideney transplant	NCT02563366	Effect of BM-MSCs on Early Graft Function Recovery After DCD Kidney Transplant.	Unknown status	IV	Allo	BMMSCs	18 Years to 65 Years	Phase 1|Phase 2	120	2015/9/30	
Kideney transplant	NCT03478215	Mesenchymal Stromal Cells in Living Donor Kidney Transplantation	Recruiting	IV	Auto	UNS	18 Years to 65 Years	Phase 2	24	2018/3/27	
Kideney transplant	NCT03504241	Tolerance by Engaging Antigen During Cellular Homeostasis	Recruiting	IV	Allo	BMMSCs	19 Years and older	Phase 1	6	2018/4/20	
Kideney transplant	NCT03585855	Mesenchymal Stem Cell Transplantation in the Treatment of Chronic Antibody Mediated Kidney Graft Rejection (ABMR)	Terminated	UNS	Auto	BMMSCs	18 Years and older	Not Applicable	4	2018/7/13	
Kideney transplant	NCT04388761	Feasibility and Safety of Allogeneic Adipose Mesenchymal Stem Cells (aMSCs) Delivery Into Kidney Allografts Procured From Deceased Donors With High Kidney Donor Profile Index (KDPI)	Not yet recruiting	Intraparenchymal injection & Intra-arterial infusion	Allo	AdMSCs	18 Years and older	Phase 2	15	2020/5/14	
Kideney transplant	NCT04445220	A Study of Cell Therapy in COVID-19 Subjects With Acute Kidney Injury Who Are Receiving Renal Replacement Therapy	Not yet recruiting	Integrated into the renal replacement circuit	Allo	UNS	18 Years and older	Phase 1|Phase 2	22	2020/6/24	
Kideney/Liver transplant	NCT01429038	Mesenchymal Stem Cells After Renal or Liver Transplantation	Unknown status	IV	Allo	BMMSCs	18 Years to 75 Years	Phase 1|Phase 2	40	2011/9/5	Detry 2017[82]
Liver transplant	NCT01690247	Human Mesenchymal Stem Cells Induce Liver Transplant Tolerance	Unknown status	IV	Allo	WJUCMSCs	18 Years to 70 Years	Phase 1	50	2012/9/21	
Liver transplant	NCT02260375	MSC Therapy in Liver Transplantation	Recruiting	IV	Allo	BMMSCs	18 Years and older	Phase 1	20	2014/10/9	
Liver transplant	NCT02706132	Therapeutic Strategy and the Role of Mesenchymal Stromal Cells for ABO Incompatible Liver Transplantation	Unknown status	IV	Auto	UNS	18 Years to 60 Years	Phase 1|Phase 2	15	2016/3/11	
Liver transplant	NCT02957552	Safety and Tolerance of Immunomodulating Therapy With Donor-specific MSC in Pediatric Living-Donor Liver Transplantation	Recruiting	IV	Allo	BMMSCs	8 Weeks to 18 Years	Phase 1	7	2016/11/6	

Lung transplant	NCT04714801	Adipose Derived Mesenchymal Cell Treatment in Lung transplantation									
Skin trasnplant	NCT03113747	Allogeneic ADSCs and Platelet-Poor Plasma Fibrin Hydrogel to Treat the Patients With Burn Wounds (ADSCs-BWs)	Unknown status	On skin	Allo	AdMSCs	18 Years to 65 Years	Phase 1|Phase 2	20	2017/4/14	
Skin transplant	NCT04234750	Mesenchymal Stem Cell-derived Pleiotropic Factor in the Treatment of Donor Sites	Recruiting	On skin	UNS	UNS-MSC-CM	6 Years to 60 Years	Phase 1	20	2020/1/21	

Table S2: Clinical trials for autoimmune diseases using MSC-based products

Conditions	NCT Number	Title	Status	Delivery	Auto/Allo?	Source MSCs	Age	Phases	Enrollment	First Posted	Publications
CD	NCT02445547	Umbilical Cord Mesenchymal Stem Cell Treatment for Crohn's Disease	Completed	IV	Allo	WJUCMSCs	18 Years to 70 Years	Phase 1|Phase 2	82	2015/5/15	
CD	NCT02000362	Safety and Efficacy of FURESTEM-CD Inj. in Patients With Moderately Active Crohn's Disease(CD)	Unknown status	UNS	Allo	UCBMSCs	19 Years to 70 Years	Phase 1|Phase 2	24	2013/12/4	
CD	NCT01915927	Stem Cell Fistula Plug in Perianal Crohn's Disease	Completed	Peri-fistula injection	Auto	AdMSCs	18 Years to 65 Years	Phase 1	20	2013/8/5	Dietz 2017[85]
CD	NCT01874015	Transplantation of Bone Marrow Mesenchymal Stem Cell in Crohn's Disease	Unknown status	Intralesional injection	Auto	BMMSCs	18 Years to 65 Years	Phase 1	10	2013/6/10	
CD	NCT01541579	Adipose Derived Mesenchymal Stem Cells for Induction of Remission in Perianal Fistulizing Crohn's Disease	Completed	Intralesional injection	Allo	AdMSCs	18 Years and older	Phase 3	278	2012/3/1	Panés 2016[84], Panés 2018[86]
CD	NCT01144962	Dose-escalating Therapeutic Study of Allogeneic Bone Marrow Derived Mesenchymal Stem Cells for the Treatment of Fistulas in Patients With Refractory Perianal Crohn's Disease	Completed	Peri-fistula injection	Allo	BMMSCs	18 Years and older	Phase 1|Phase 2	21	2010/6/16	Molendijk 2015[83]
CD	NCT00482092	Evaluation of PROCHYMAL® Adult Human Stem Cells for Treatment-resistant Moderate-to-severe Crohn's Disease	Completed	IV	Allo	BMMSCs	18 Years to 70 Years	Phase 3	330	2007/6/4	
CD	NCT00543374	Extended Evaluation of PROCHYMAL® Adult Human Stem Cells for Treatment-Resistant Moderate-to-Severe Crohn's Disease	Completed	IV	Allo	BMMSCs	18 Years to 70 Years	Phase 3	98	2007/10/15	
CD	NCT00294112	Prochymal® Adult Human Mesenchymal Stem Cells for Treatment of Moderate-to-severe Crohn's Disease	Completed	IV	Allo	BMMSCs	18 Years to 70 Years	Phase 2	10	2006/2/20	

CD	NCT04073472	Mesenchymal Stem Cells for the Treatment of Pouch Fistulas in Crohn's	Not yet recruiting	Peri-fistula injection	Allo	BMMSCs	18 Years to 75 Years	Phase 1	15	2019/8/29	
CD	NCT04519684	Study of Mesenchymal Stem Cells for the Treatment of Ileal Pouch Fistula's in Participants With Crohn's Disease	Not yet recruiting	Peri-fistula injection	Allo	BMMSCs	18 Years to 75 Years	Phase 1|Phase 2	20	2020/8/20	
CD	NCT04519697	Mesenchymal Stem Cells for the Treatment of Rectovaginal Fistulas in Participants With Crohn's Disease	Not yet recruiting	Peri-fistula injection	Allo	BMMSCs	18 Years to 75 Years	Phase 1|Phase 2	20	2020/8/20	
CD	NCT04519671	Mesenchymal Stem Cells for the Treatment of Perianal Fistulizing Crohn's Disease	Not yet recruiting	Peri-fistula injection	Allo	BMMSCs	18 Years to 75 Years	Phase 1|Phase 2	20	2020/8/20	
CD	NCT01659762	A Phase I Study Evaluating Autologous Bone Marrow Derived Mesenchymal Stromal for Crohn's Disease.	Completed	IV	Auto	BMMSCs	18 Years to 65 Years	Phase 1	16	2012/8/8	
CD	NCT01090817	An Australian Study of Mesenchymal Stromal Cells for Crohn's Disease	Completed	IV	Allo	UNS	18 Years to 55 Years	Phase 2	21	2010/3/23	Forbes 2014[87]
CD	NCT03555773	Micro-fragmented Adipose Tissue and Complex Crohns' Anal Fistulas	Completed	Peri-fistula injection	Auto	AdMSCs	18 Years and older	Not Applicable	15	2018/6/14	
CD	NCT02926300	Long-term Safety and Efficacy of FURESTEM-CD Inj. in Patients With Moderately Active Crohn's Disease(CD)	Recruiting	UNS	Allo	UCBMSCs	19 Years to 70 Years	Not Applicable	24	2016/10/6	
CD	NCT03209700	Re-treatment From a Phase I Study of MSC-AFP in Patients With Perianal Fistulas	Completed	Peri-fistula injection	Auto	AdMSCs	18 Years to 65 Years	Phase 1	7	2017/7/6	
CD	NCT03014219	Phase 1 Crohn's Pediatric Sub-study of MSC AFP	Withdrawn	Peri-fistula injection	Auto	AdMSCs	12 Years to 17 Years	Phase 1	0	2017/1/9	
CD	NCT03449069	Pediatric MSC-AFP Sub-study for Crohn's Fistula	Recruiting	Peri-fistula injection	Auto	UNS	12 Years to 17 Years	Phase 1	5	2018/2/28	
CD	NCT03901235	MSC Intratissular Injection in Crohn Disease Patients	Recruiting	Intratissular injection	UNS	UNS	18 Years and older	Phase 1|Phase 2	60	2019/4/3	

CD	NCT01540292	Mesenchymal Stem Cell Therapy for the Treatment of Severe or Refractory Inflammatory and/or Autoimmune Disorders	Unknown status	IV	Allo	BMMSCs	18 Years to 75 Years	Phase 1|Phase 2	20	2012/2/28	
CD	NCT04548583	Study of Mesenchymal Stem Cells for the Treatment of Medically Refractory Crohn's Colitis	Recruiting	Submucosal injection in colon	Allo	BMMSCs	18 Years to 75 Years	Phase 1|Phase 2	24	2020/9/14	
CD	NCT04519671	Mesenchymal Stem Cells for the Treatment of Perianal Fistulizing Crohn's Disease	Recruiting	Peri-fistula injection	Allo	BMMSCs	18 Years to 75 Years	Phase 1|Phase 2	20	2020/8/20	
CD	NCT04791878	Study of Mesenchymal Stem Cells for Pediatric Perianal Fistulizing Crohn's Disease	Not yet recruiting	Peri-fistula injection	Allo	BMMSCs	13 Years to 17 Years	Phase 1	10	2021/3/10	
IBD	NCT01157650	Treatment of Fistulous Crohn's Disease by Implant of Autologous Mesenchymal Stem Cells Derived From Adipose Tissue	Completed	UNS	Auto	AdMSCs	18 Years and older	Phase 1|Phase 2	15	2010/7/7	
IBD	NCT03299413	Use of Mesenchymal Stem Cells in Inflammatory Bowel Disease	Active, not recruiting	IV	Allo	WJUCMSCs	18 Years to 75 Years	Phase 1|Phase 2	20	2017/10/3	
IBD	NCT02677350	AlloGeneic Human Mesenchymal Stem Cells (hMSC) in PAtients With FistuLizing Crohn's Disease Via PErifistula iNjEctions (GALENE)	Withdrawn	Peri-fistula injection	Allo	BMMSCs	18 Years and older	Phase 1	0	2016/2/9	
UC	NCT02442037	Human Umbilical-Cord-Derived Mesenchymal Stem Cell Therapy in Active Ulcerative Colitis	Unknown status	IV	Allo	WJUCMSCs	18 Years to 65 Years	Phase 1|Phase 2	30	2015/5/13	
UC	NCT01221428	Umbilical Cord Mesenchymal Stem Cells Infusion for Ulcerative Colitis	Unknown status	IV	Allo	WJUCMSCs	18 Years to 70 Years	Phase 1|Phase 2	50	2010/10/15	Hu 2016[88]
UC	NCT01914887	Allogeneic Adipose Tissue-derived Mesenchymal Stem Cells for the Induction of Remission in Ulcerative Colitis	Unknown status	Endoscopic injection	Allo	AdMSCs	18 Years and older	Phase 1|Phase 2	8	2013/8/2	
UC	NCT04543994	Study of Mesenchymal Stem Cells for the Treatment of Medically Refractory Ulcerative Colitis (UC)	Not yet recruiting	Submucosal injection in colon	Allo	BMMSCs	18 Years to 75 Years	Phase 1|Phase 2	24	2020/9/10	
UC	NCT04312113	Angiographic Delivery of AD-MSC for Ulcerative Colitis	Not yet recruiting	Intraarterial injection	Auto	AdMSCs	18 Years to 65 Years	Phase 1	20	2020/3/18	

UC	NCT03609905	Adipose Mesenchymal Stem Cells (AMSC) for Treatment of Ulcerative Colitis	Recruiting	Submucosal injection in colon	Auto	AdMSCs	18 Years to 65 Years	Phase 1|Phase 2	50	2018/8/1	
MS	NCT00395200	Mesenchymal Stem Cells in Multiple Sclerosis (MSCIMS)	Completed	IV	Auto	BMMSCs	18 Years to 65 Years	Phase 1|Phase 2	10	2006/11/2	Connick 2011[90], Connick 2012[91]
MS	NCT00781872	Mesenchymal Stem Cells for the Treatment of MS	Completed	Intrathecal injection	Auto	BMMSCs	35 Years to 65 Years	Phase 1|Phase 2	20	2008/10/29	Karussis 2010[89]
MS	NCT00813969	Autologous Mesenchymal Stem Cell (MSC) Transplantation in MS	Completed	IV	Auto	UNS	18 Years to 55 Years	Phase 1	24	2008/12/23	Cohen 2018[93]
MS	NCT01056471	Autologous Mesenchymal Stem Cells From Adipose Tissue in Patients With Secondary Progressive Multiple Sclerosis	Completed	IV	Auto	AdMSCs	18 Years and older	Phase 1|Phase 2	30	2010/1/26	
MS	NCT01228266	Mesenchymal Stem Cell Transplantation in MS	Terminated	IV	Auto	UNS	18 Years to 50 Years	Phase 2	9	2010/10/26	Llufriu 2014[92]
MS	NCT01364246	Safety and Efficacy of Umbilical Cord Mesenchymal Stem Cell Therapy for Patients With Progressive Multiple Sclerosis and Neuromyelitis Optica	Unknown status	UNS	Allo	WJUCMSCs	18 Years to 60 Years	Phase 1|Phase 2	20	2011/6/2	
MS	NCT01377870	Evaluation of Autologous Mesenchymal Stem Cell Transplantation (Effects and Side Effects) in Multiple Sclerosis	Completed	IV	Auto	BMMSCs	18 Years to 55 Years	Phase 1|Phase 2	22	2011/6/21	
MS	NCT01606215	Stem Cells in Rapidly Evolving Active Multiple Sclerosis	Completed	IV	Auto	BMMSCs	18 Years to 50 Years	Phase 1|Phase 2	21	2012/5/25	Uccelli 2019[94]
MS	NCT01730547	Mesenchymal Stem Cells for Multiple Sclerosis	Unknown status	IV	Auto	UNS	18 Years to 50 Years	Phase 1|Phase 2	15	2012/11/21	Uccelli 2019[94]
MS	NCT01745783	Mesenchymal Cells From Autologous Bone Marrow, Administered Intravenously in Patients Diagnosed With Multiple Sclerosis	Recruiting	IV	Auto	BMMSCs	18 Years to 50 Years	Phase 1|Phase 2	30	2012/12/10	Uccelli 2019[94]
MS	NCT01854957	MEsenchymal StEm Cells for Multiple Sclerosis	Unknown status	IV	Auto	UNS	18 Years to 50 Years	Phase 1|Phase 2	20	2013/5/16	Uccelli 2019[94]
MS	NCT01895439	Safety and Efficacy Study of Autologus Bone Marrow Mesenchymal Stem Cells in Multiple Sclerosis	Completed	Intrathecal injection	Auto	BMMSCs	18 Years to 65 Years	Phase 1|Phase 2	13	2013/7/10	

MS	NCT02034188	Feasibility Study of Human Umbilical Cord Tissue-Derived Mesenchymal Stem Cells in Patients With Multiple Sclerosis	Completed	IV	Allo	WJUCMSCs	18 Years to 55 Years	Phase 1|Phase 2	20	2014/1/13	Riordan 2018[95]
MS	NCT02035514	Phase I-II Clinical Trial With Autologous Bone Marrow Derived Mesenchymal Stem Cells for the Therapy of Multiple Sclerosis	Completed	IV	Auto	BMMSCs	18 Years to 50 Years	Phase 1|Phase 2	9	2014/1/14	Uccelli 2019[94]
MS	NCT02166021	Clinical Efficacy of Autologous Mesenchymal Bone Marrow Stem Cells in Active & Progressive Multiple Sclerosis	Completed	IV/Intrathecal injection	Auto	BMMSCs	18 Years to 65 Years	Phase 2	48	2014/6/18	
MS	NCT02239393	Safety and Efficacy of Intravenous Autologous Mesenchymal Stem Cells for MS: a Phase 2 Proof of Concept Study	Completed	IV	Auto	UNS	18 Years to 50 Years	Phase 2	31	2014/9/12	Uccelli 2019[94]
MS	NCT02326935	Multi-Center Study Safety of Adipose Derived Mesenchymal Stem Cells for the Treatment of Multiple Sclerosis	Terminated	IV	Auto	AdMSCs	18 Years to 65 Years	Phase 1	2	2014/12/30	
MS	NCT02403947	MEsenchymal StEm Cells for Multiple Sclerosis	Terminated	IV	Auto	BMMSCs	18 Years to 50 Years	Phase 1|Phase 2	1	2015/3/31	Uccelli 2019[94]
MS	NCT02418325	A Study of Allogeneic Human UC-MSC and Liberation Therapy (When Associated With CCSVI) in Patients With RRMS	Terminated	IV/Intrathecal injection	Allo	WJUCMSCs	18 Years to 60 Years	Phase 1|Phase 2	69	2015/4/16	
MS	NCT02495766	Autologous Mesenchymal Stromal Cells for Multiple Sclerosis	Completed	IV	Auto	BMMSCs	18 Years to 60 Years	Phase 1|Phase 2	8	2015/7/13	
MS	NCT02587715	A Study of Allogeneic Human UC-MSC and Liberation Therapy (When Associated With CCSVI) in Patients With RRMS	Unknown status	IV/Intrathecal injection	Allo	WJUCMSCs	18 Years to 60 Years	Phase 1|Phase 2	69	2015/10/27	
MS	NCT03326505	Allogenic Mesenchymal Stem Cells And Physical Therapy for MS Treatment	Completed	Intrathecal injection	Allo	WJUCMSCs	18 Years to 65 Years	Phase 1|Phase 2	60	2017/10/31	Alghwiri 2020[96]
MS	NCT03778333	Mesenchymal Stem Cells for Progressive Multiple Sclerosis_Sweden	Completed	IV	Auto	BMMSCs	18 Years to 65 Years	Phase 1	7	2018/12/19	
MS	NCT03799718	Safety and Efficacy of Repeated Administration of NurOwn (MSC-NTF Cells) in Participants With Progressive MS	Recruiting	Intrathecal injection	Auto	BMMSC-NF	18 Years to 65 Years	Phase 2	20	2019/1/10	
MS	NCT04749667	Study of Mesenchymal Autologous Stem Cells as Regenerative Treatment for Multiple Sclerosis	Not yet recruiting	Intrathecal injection	Auto	BMMSCs	18 Years to 55 Years	Phase 1|Phase 2	18	2021/2/11	

T1DM	NCT00690066	PROCHYMAL® (Human Adult Stem Cells) for the Treatment of Recently Diagnosed Type 1 Diabetes Mellitus (T1DM)	Completed	IV	Allo	BMMSCs	12 Years to 35 Years	Phase 2	63	2008/6/4	
T1DM	NCT01068951	Treatment of Patients With Newly Onset of Type 1 Diabetes With Mesenchymal Stem Cells	Completed	IV	Auto	BMMSCs	18 Years to 40 Years	Not Applicable	20	2010/2/17	Carlsson 2015[103]
T1DM	NCT01157403	Autologous Transplantation of Mesenchymal Stem Cells for Treatment of Patients With Onset of Type 1 Diabetes	Unknown status	IV	Auto	BMMSCs	10 Years to 40 Years	Phase 2|Phase 3	80	2010/7/7	
T1DM	NCT01219465	Umbilical Cord Mesenchymal Stem Cells Infusion for Initial Type 1 Diabetes Mellitus	Unknown status	IV	Allo	WJUCMSCs	3 Years to 35 Years	Phase 1|Phase 2	50	2010/10/13	
T1DM	NCT01322789	Safety and Efficacy of Mesenchymal Stem Cells in Newly-diagnosed Type 1 Diabetic Patients	Unknown status	IV	UNS	BMMSCs	12 Years to 35 Years	Phase 1|Phase 2	10	2011/3/25	
T1DM	NCT01374854	Umbilical Mesenchymal Stem Cells and Mononuclear Cells Infusion in Type 1 Diabetes Mellitus	Unknown status	Intraarterial injection	Allo	WJUCMSCs	18 Years to 40 Years	Phase 1|Phase 2	44	2011/6/16	Cai 2016[104]
T1DM	NCT01496339	Human Menstrual Blood-derived Mesenchymal Stem Cells Transplantation in Treating Type 1 Diabetic Patients	Unknown status	IV	Allo	MenSCs	18 Years to 75 Years	Phase 1|Phase 2	50	2011/12/21	
T1DM	NCT02057211	Mesenchymal Stem Cells to Intervene in the Development of Type 1 Diabetes: a Blinded Randomized Study	Suspended	UNS	Auto	UNS	18 Years to 40 Years	Phase 2	50	2014/2/7	
T1DM	NCT02138331	Effect of Microvesicles and Exosomes Therapy on β-cell Mass in Type I Diabetes Mellitus (T1DM)	Unknown status	IV	Allo	UCBMSC-exosome	18 Years to 60 Years	Phase 2|Phase 3	20	2014/5/14	
T1DM	NCT02893306	MSC Administration for the Management of Type 1 Diabetic Patients	Unknown status	IV	Allo	BMMSCs	18 Years and older	Phase 2	10	2016/9/8	
T1DM	NCT02940418	Use of Stem Cells in Diabetes Mellitus Type 1	Recruiting	IV	Allo	AdMSCs	18 Years to 35 Years	Phase 1	20	2016/10/20	
T1DM	NCT03406585	Wharton's Jelly Derived Mesenchymal Stromal Cell Treatment of Adult Patients Diagnosed With Type I Diabetes	Recruiting	IV	Allo	WJUCMSCs	18 Years to 40 Years	Phase 1|Phase 2	24	2018/1/23	
T1DM	NCT03484741	Mesenchymal Stem Cell Therapy for Type 1 Diabetes Mellitus Patients	Unknown status	IV	Auto/Allo	BMMSCs/WJUCMSCs	18 Years to 45 Years	Phase 1|Phase 2	15	2018/4/2	
T1DM	NCT03920397	Mesenchymal Stem Cells in Patients With Type 1 Diabetes Mellitus	Enrolling by invitation	IV	Allo	AdMSCs	16 Years to 35 Years	Not Applicable	30	2019/4/18	Araujo 2020[105]

T1DM	NCT03973827	Wharton's Jelly Derived Mesenchymal Stromal Cell Repeated Treatment of Adult Patients Diagnosed With Type I Diabetes	Recruiting	UNS	Allo	WJUCMSCs	18 Years to 64 Years	Phase 1|Phase 2	18	2019/6/4	
T1DM	NCT04061746	Cellular Therapy for Type 1 Diabetes Using Mesenchymal Stem Cells	Recruiting	IV	Allo	WJUCMSCs	12 Years to 30 Years	Phase 1	50	2019/8/20	
T1DM	NCT04078308	Mesenchymal Stem Cells Transplantation in Newly Diagnosed Type-1 Diabetes Patients	Active, not recruiting	IV	Auto	BMMSCs	8 Years to 40 Years	Phase 1|Phase 2	20	2019/9/6	
T1DM	NCT01143168	Stem Cell Therapy for Type 1 Diabetes Mellitus	Unknown status	IV	Auto/Allo	BMMSCs/WJUCMSCs	18 Years to 50 Years	Phase 1	24	2010/6/14	
SLE	NCT00659217	Effect of Mesenchymal Stem Cell Transplantation for Lupus Nephritis	Unknown status	UNS	Auto	UNS	18 Years to 60 Years	Phase 1|Phase 2	20	2008/4/16	
SLE	NCT03174587	Evaluate the Safety of CS20AT04 Inj. in Subjects With Lupus Nephritis	Completed	IV	Allo	BMMSCs	18 Years to 69 Years	Phase 1	7	2017/6/2	
SLE	NCT03458156	Umbilical Cord Mesenchymal Stem Cell Transplantation for Lupus Nephritis	Unknown status	UNS	Allo	WJUCMSCs	18 Years to 60 Years	Phase 1|Phase 2	30	2018/3/8	
SLE	NCT03673748	Treatment of Lupus Nephritis With Allogeneic Mesenchymal Stem Cells	Not yet recruiting	IV	Allo	UNS	18 Years to 65 Years	Phase 2	36	2018/9/17	
SLE	NCT03917797	Mesenchymal Stromal Cells (MSC's) in Renal Lupus	Recruiting	IV	Allo	WJUCMSCs	18 Years to 75 Years	Phase 2	39	2019/4/17	
SLE	NCT04318600	Allogeneic Amniotic Mesenchymal Stem Cell Therapy for Lupus Nephritis	Completed	IV	Allo	Amniotic MSCs	14 Years to 60 Years	Phase 1	16	2020/3/24	
SLE	NCT04522505	Long-term Follow-up for Evaluating the Safety of CS20AT04 in Subject With Lupus Nephritis	Active, not recruiting	UNS	Allo	BMMSCs	17 Years to 70 Years	Phase 1|Phase 2	7	2020/8/21	
SLE	NCT03580291	Human Umbilical Cord Mesenchymal Stem Cells Treatment for Lupus Nephritis (LN)	Not yet recruiting	IV	Allo	WJUCMSCs	18 Years to 60 Years	Phase 2	230	2018/7/9	
SLE	NCT00698191	Mesenchymal Stem Cells Transplantation for Refractory Systemic Lupus Erythematosus (SLE)	Unknown status	IV	Allo	BMMSCs	15 Years to 70 Years	Phase 1|Phase 2	20	2008/6/17	Liang 2018[106]
SLE	NCT01539902	Phase 2 Study of Human Umbilical Cord Derived Mesenchymal Stem Cell for the Treatment of Lupus Nephritis	Unknown status	UNS	Allo	WJUCMSCs	16 Years to 65 Years	Phase 2	25	2012/2/28	Deng 2017[107]

SLE	NCT01741857	Umbilical Cord Derived Mesenchymal Stem Cells Transplantation for Active and Refractory Systemic Lupus Erythematosus	Unknown status	UNS	Allo	WJUCMSCs	15 Years to 60 Years	Phase 1|Phase 2	40	2012/12/5	Wang 2014[109], Wang 2014[108]
SLE	NCT02633163	Phase 2 Trial of Mesenchymal Stem Cells in Systemic Lupus Erythematosus (MiSLE)	Recruiting	IV	Allo	WJUCMSCs	18 Years to 65 Years	Phase 2	81	2015/12/17	
SLE	NCT03171194	Pilot Trial of Mesenchymal Stem Cells for Systemic Lupus Erythematosus	Completed	IV	Allo	WJUCMSCs	18 Years to 65 Years	Phase 1	6	2017/5/31	
SLE	NCT03219801	Umbilical Cord Derived Mesenchymal Stem Cells Therapy in Systemic Lupus Erythematosus	Unknown status	IV	Allo	WJUCMSCs	14 Years to 60 Years	Early Phase 1	10	2017/7/18	
SLE	NCT03562065	Treatment of Refractory Systemic Lupus Erythematosus by Allogeneic Mesenchymal Stem Cells Derived From the Umbilical Cord	Not yet recruiting	IV	Allo	WJUCMSCs	18 Years to 70 Years	Phase 1|Phase 2	10	2018/6/19	
SLE	NCT04184258	Treatment of Systemic Lupus Erythematosus With Pooled Allogenic Mesenchymal Stem Cells	Recruiting	UNS	Allo	OMMSCs	18 Years to 75 Years	Phase 1|Phase 2	10	2019/12/3	
RA	NCT01985464	Umbilical Cord Tissue-derived Mesenchymal Stem Cells for Rheumatoid Arthritis	Active, not recruiting	IV	Allo	WJUCMSCs	18 Years and older	Phase 1|Phase 2	20	2013/11/15	
RA	NCT01547091	Safety and Efficacy Study of Umbilical Cord-Derived Mesenchymal Stem Cells for Rheumatoid Arthritis	Unknown status	IV	Allo	WJUCMSCs	18 Years to 70 Years	Phase 1|Phase 2	200	2012/3/7	
RA	NCT03828344	Safety and Tolerability of a Single Intravenous Infusion of BX-U001 in Refractory Rheumatoid Arthritis	Not yet recruiting	IV	Allo	WJUCMSCs	18 Years to 70 Years	Phase 1	16	2019/2/4	
RA	NCT03691909	A Clinical Trial to Determine the Safety and Efficacy of Hope Biosciences Autologous Mesenchymal Stem Cell Therapy (HB-adMSCs) for the Treatment of Rheumatoid Arthritis	Active, not recruiting	IV	Auto	AdMSCs	18 Years to 65 Years	Phase 1|Phase 2	15	2018/10/2	
RA	NCT03798028	The Safety and Effects of Mesenchymal Stem Cell (MSCs) in the Treatment of Rheumatoid Arthritis	Recruiting	IV	Allo	UCBMSCs	18 Years to 70 Years	Phase 1|Phase 2	250	2019/1/9	
RA	NCT03186417	Mesenchymal Stem Cells in Early Rheumatoid Arthritis	Recruiting	IV	Allo	UNS	18 Years to 80 Years	Phase 1	20	2017/6/14	
RA	NCT01663116	Cx611-0101, eASCs Intravenous Administration to Refractory Rheumatoid Arthritis Patients	Completed	IV	Allo	AdMSCs	18 Years and older	Phase 1|Phase 2	53	2012/8/13	Álvaro-Gracia 2017[97]

RA	NCT01873625	Transplantation of Bone Marrow Derived Mesenchymal Stem Cells in Affected Knee Osteoarthritis by Rheumatoid Arthritis	Completed	Intraarticular	Auto	BMMSCs	10 Years to 65 Years	Phase 2|Phase 3	60	2013/6/10	Shadmanfar 2018[98]
RA	NCT02643823	Human Umbilical Cord-Mesenchymal Stem Cells for Rheumatoid Arthritis	Unknown status	IV	Allo	WJUCMSCs	18 Years to 80 Years	Phase 1	40	2015/12/31	
RA	NCT04170426	Autologous Adipose-derived Stem Cells (AdMSCs) for Rheumatoid Arthritis	Not yet recruiting	IV	Auto	AdMSCs	18 Years to 75 Years	Phase 1|Phase 2	54	2019/11/20	
RA	NCT03067870	Transplantation of Autologous Bone Marrow Derived Stem Cells in Patients With Rheumatoid Arthritis	Active, not recruiting	IV	Auto	BMMSCs	17 Years to 75 Years	Phase 1	100	2017/3/1	
RA	NCT03333681	Evaluation of Stem Cell Therapy Effects on the Immune Response in Rheumatoid Arthritis Patients	Completed	IV	Auto	BMMSCs	35 Years to 60 Years	Phase 1	15	2017/11/7	
Psioriasis	NCT02491658	Safety and Efficacy of UC-MSCs in Patients With Psoriasis Vulgaris	Unknown status	IV	Allo	WJUCMSCs	18 Years to 65 Years	Phase 1|Phase 2	30	2015/7/8	
Psioriasis	NCT02918123	Safety of FURESTEM-CD Inj. in Patients With Moderate to Severe Plaque-type Psoriasis	Recruiting	SQ	Allo	UCBMSCs	19 Years to 65 Years	Phase 1	9	2016/9/28	
Psioriasis	NCT03265613	Safety and Efficacy of Expanded Allogeneic AD-MSCs in Patients With Moderate to Severe Psoriasis	Active, not recruiting	IV	Allo	AdMSCs	18 Years to 65 Years	Phase 1|Phase 2	7	2017/8/29	
Psioriasis	NCT03392311	Efficacy and Safety of AD-MSCs Plus Calpocitriol Ointment in Patients With Moderate to Severe Psoriasis	Recruiting	IV	Allo	AdMSCs	18 Years to 65 Years	Phase 1|Phase 2	5	2018/1/5	
Psioriasis	NCT03424629	Safety and Efficacy of UC-MSCs in Patients With Plaque Psoriasis	Unknown status	UNS	Allo	WJUCMSCs	18 Years to 60 Years	Phase 1	57	2018/2/7	
Psioriasis	NCT03745417	Efficacy and Safety of Expanded UCMSCs On Patients With Moderate to Severe Psoriasis	Recruiting	IV	Allo	WJUCMSCs	18 Years to 65 Years	Phase 1|Phase 2	5	2018/11/19	
Psioriasis	NCT03765957	Clinical Research on Treatment of Psoriasis by Human Umbilical Cord-derived Mesenchymal Stem Cells	Not yet recruiting	IV	Allo	WJUCMSCs	18 Years to 65 Years	Early Phase 1	50	2018/12/5	
Psioriasis	NCT04275024	Efficacy and Safety of AD-MSCs Plus Calpocitriol Ointment and PSORI-CM01 Granule in Psoriasis Patients	Recruiting	IV	UNS	AdMSCs	18 Years to 65 Years	Phase 1|Phase 2	8	2020/2/19	
Psioriasis	NCT04785027	Comparison of PSORI-CM01 Formula vs Gu Ben Hua Yu Formula Combined With AD-MSCs in Psoriasis	Recruiting	IV	Allo	AdMSCs	18 Years to 65 Years	Phase 1|Phase 2	16	2021/3/5	

Acquired Aplastic Anemia	NCT01297972	Mesenchymal Stem Cells in the Treatment of Relapsed/Refractory Severe Acquired Aplastic Anemia	Completed	IV	Allo	BMMSCs	18 Years to 65 Years	Phase 1|Phase 2	9	2011/2/17	Clé 2015[99]
Autoimmune hepatitis	NCT01661842	Umbilical Cord Mesenchymal Stem Cells for Patients With Autoimmune Hepatitis	Unknown status	IV	Allo	WJUCMSCs	18 Years to 65 Years	Phase 1|Phase 2	100	2012/8/10	
Immune Thrombocytopenia	NCT04014166	Study of Human Umbilical Cord-derived Mesenchymal Stem Cells for Treatment of Refractory Immune Thrombocytopenia	Recruiting	UNS	Allo	WJUCMSCs	18 Years to 60 Years	Phase 1|Phase 2	15	2019/7/10	
Primary biliary cirrhosis	NCT01440309	Efficacy and Safety Study of Allogenic Mesenchymal Stem Cells for Patients With Refractory Primary Biliary Cirrhosis	Unknown status	IV	Allo	BMMSCs	18 Years to 60 Years	Phase 1	20	2011/9/26	
Primary Biliary Cirrhosis	NCT01662973	Umbilical Cord Mesenchymal Stem Cells for Patients With Primary Biliary Cirrhosis	Unknown status	IV	Allo	WJUCMSCs	18 Years to 65 Years	Phase 1|Phase 2	100	2012/8/13	
Primary Biliary Cirrhosis	NCT04522869	Umbilical Cord Derived Mesenchymal Stem Cell (UC -MSC) Transplantation for Children Suffering From Biliary Atresia	Recruiting	Hepatic arterial injection	Allo	WJUCMSCs	5 Months to 2 Years	Phase 1|Phase 2	34	2020/8/21	
Primary Sclerosing Cholangitis	NCT02997878	A Single-arm,Phase IIa,Safety and Efficacy Trial of Selected MSCs in the Treatment of Patients With PSC & AiH	Recruiting	IV	Allo	WJUCMSCs	18 Years to 70 Years	Phase 1|Phase 2	56	2016/12/20	
Severe Aplastic Anemia	NCT02218437	Treatment Protocol of Child SAA With the Injection of Mesenchymal Stem Cells (Umbilical Cord Derived) ,MSC-SAA"	No Results Available	UNS	Allo	WJUCMSCs	1 Month to 18 Years	Phase 4	20	2014/8/18	
Primary Sjögren's Syndrome	NCT00953485	Allogeneic Mesenchymal Stem Cells Transplantation for Primary Sjögren's Syndrome (pSS)	Unknown status	IV	Allo	BMMSCs	15 Years to 70 Years	Phase 1|Phase 2	20	2009/8/6	Xu 2012[100]
Sjögren's Syndrome	NCT04615455	Mesenchymal Stem Cell Therapy of Dry Eye Disease in Patients With Sjögren's Syndrome	Recruiting	Transconjunctival injection	Allo	AdMSCs	18 Years and older	Phase 2	40	2020/11/4	
Systemic Sclerosis	NCT00962923	Allogeneic Mesenchymal Stem Cells Transplantation for Systemic Sclerosis (SSc)	Unknown status	IV	Allo	WJUCMSCs	15 Years to 65 Years	Phase 1|Phase 2	20	2009/8/20	Zhang 2017[101]

Systemic Sclerosis	NCT02213705	Treatment of Refractory Sever Systemic Scleroderma by Injection of Allogeneic Mesenchymal Stem Cells	Unknown status	UNS	Allo	UNS	18 Years to 70 Years	Phase 1|Phase 2	20	2014/8/11	
Systemic Sclerosis	NCT03211793	Mesenchymal Stromal Cells as Treatment for Digital Ulcers in Systemic Sclerosis	Not yet recruiting	IM	Allo	BMMSCs	18 Years and older	Phase 1|Phase 2	20	2017/7/7	van Rhijn-Brouwer 2018[102]
Systemic Sclerosis	NCT04356287	Treatment With Human Umbilical Cord-derived Mesenchymal Stromal Cells in Systemic Sclerosis	Not yet recruiting	IV	Allo	WJUCMSCs	18 Years and older	Phase 1|Phase 2	18	2020/4/22	
Systemic Sclerosis	NCT04432545	Infusion of Allogeneic Mesenchymal Stem Cells in Patients With Diffuse Cutaneous Systemic Sclerosis With Refractory Pulmonary Involvement	Available	IV	Allo	WJUCMSCs	18 Years to 65 Years	Not Applicable		2020/6/16	

Table S3: Clinical trials for OA using MSC-based products

Conditions	NCT Number	Title	Status	Delivery	Auto/ Allo?	Source MSCs	Age	Phases	Enrollment	First Posted	Publications
OA	NCT02291926	Human Umbilical Cord Mesenchymal Stem Cell Transplantation in Articular Cartilage Defect	Completed	Intraarticular injection	Allo	WJUCMSCs	18 Years to 75 Years	Phase 1	20	2014/11/17	
OA	NCT02237846	Clinical Study of Umbilical Cord Tissue Mesenchymal Stem Cells (UC-MSC) for Treatment of Osteoarthritis	Withdrawn	Intraarticular injection/IV	Allo	WJUCMSCs	18 Years to 80 Years	Phase 1|Phase 2	0	2014/9/11	
OA	NCT02580695	A Study to Assess Safety and Efficacy of Umbilical Cord-derived Mesenchymal Stromal Cells in Knee Osteoarthritis	Completed	Intraarticular injection	Allo	WJUCMSCs	18 Years to 70 Years	Phase 1|Phase 2	30	2015/10/20	Matas 2019[133]
OA	NCT04314661	Comparative Effectiveness of Arthroscopy and Non-Arthroscopy Using Mesenchymal Stem Cell Therapy (MSCs) and Conditioned Medium for Osteoartrithis	Recruiting	Intraarticular injection	Allo	WJUCMSC-CM	55 Years to 70 Years	Phase 1|Phase 2	15	2020/3/19	
OA	NCT03800810	Implantation of Allogenic Mesenchymal Stem Cell From Umbilical Cord Blood for Osteoarthritis Management	Recruiting	Intraarticular injection	Allo	WJUCMSCs	30 Years to 80 Years	Early Phase 1	9	2019/1/11	
OA	NCT03810521	Dose-escalation of Cellistem-OA in Patients With Knee Osteoarthritis	Recruiting	Intraarticular injection	Allo	WJUCMSCs	30 Years to 75 Years	Phase 1	24	2019/1/18	
OA	NCT03866330	Wharton's Jelly-derived Mesenchymal Stem Cells in Osteoarthritis	Recruiting	Intraarticular injection	Allo	WJUCMSCs	30 Years to 75 Years	Phase 1|Phase 2	100	2019/3/7	

OA	NCT03383081	The Safety/Efficacy of Human Umbilical Cord Mesenchymal Stem Cells (19#iSCLife®-OA)Therapy for Patients With Osteoarthritis	Not yet recruiting	Intraarticular injection	Allo	WJUCMSCs	up to 70 Years	Phase 2	60	2017/12/26	
OA	NCT03166865	Clinical Study of Umbilical Cord Mesenchymal Stem Cells (UC-MSC) for Treatment of Knee Osteoarthritis	Recruiting	Intraarticular injection	Allo	WJUCMSCs	30 Years to 70 Years	Phase 1|Phase 2	60	2017/5/25	
OA	NCT03357770	The Maximum Tolerated Dose of Mesenchymal Stem Cells From Umbilical Cord	Not yet recruiting	Intraarticular injection	Allo	WJUCMSCs	18 Years to 75 Years	Not Applicable	9	2017/11/30	
OA	NCT03358654	Evaluating Safety and Efficacy of Mesenchymal Stem Cells From Umbilical Cord	Not yet recruiting	Intraarticular injection	Allo	WJUCMSCs	18 Years to 75 Years	evaluate the safety and efficacy	9	2017/11/30	
OA	NCT02118519	Mesenchymal Stem Cells in Knee Cartilage Injuries	Completed	Intraarticular injection	Auto	BMMSCs	40 Years to 68 Years	Phase 2	13	2014/4/21	Al-Najar 2017[121]
OA	NCT02162693	Clinical Trial of Autologous Adipose Tissue-Derived Mesenchymal Progenitor Cells (MPCs) Therapy for Knee Osteoarthritis	Completed	Intraarticular injection	Auto	AdMSCs	18 Years to 70 Years	Phase 2	53	2014/6/13	Lu 2019[127]
OA	NCT02037204	IMPACT: Safety and Feasibility of a Single-stage Procedure for Focal Cartilage Lesions of the Knee.	Completed	On cartilage lesions	Allo	Unspecified	18 Years to 45 Years	Phase 1|Phase 2	35	2014/1/15	
OA	NCT02658344	Clinical Trial to Evaluate Efficacy and Safety of JOINTSTEM in Patients With Degenerative Arthritis	Completed	UNS	Auto	AdMSCs	18 Years and older	Phase 2	24	2016/1/18	
OA	NCT02544802	Mesenchymal Stem Cell Treatment for Primary Osteoarthritis Knee	Unknown status	Intraarticular injection	Auto	AdMSCs	50 Years to 70 Years	Phase 1	4	2015/9/9	
OA	NCT02351011	Human Autologous MSCs for the Treatment of Mid to Late Stage Knee OA	Completed	UNS	Auto	BMMSCs	40 Years to 65 Years	Phase 1|Phase 2	12	2015/1/30	
OA	NCT02365142	Treatment of Osteoarthritis by Intra-articular Injection of Bone Marrow Mesenchymal Stem Cells With Platelet Rich Plasma	Unknown status	Intraarticular injection	Auto	BMMSCs	40 Years to 80 Years	Phase 1|Phase 2	38	2015/2/18	Lamo-Espinosa 2020[123]
OA	NCT01739504	Autologous Adipose-Derived Stromal Cells Delivered Intra-articularly in Patients With Osteoarthritis.	Terminated	Intraarticular injection	Auto	AdMSCs	18 Years to 80 Years	Assess the Safety and Effects	10	2012/12/3	

OA	NCT01809769	Autologous Adipose Tissue Derived Mesenchymal Stem Cells Therapy for Patients With Knee Osteoarthritis	Completed	Intraarticular injection	Auto	AdMSCs	40 Years to 70 Years	Phase 1|Phase 2	18	2013/3/13	Song 2018[126]
OA	NCT01985633	Mesenchymal Stem Cells Enhanced With PRP Versus PRP In OA Knee	Unknown status	Intraarticular injection	Auto	BMMSCs	40 Years to 75 Years	Phase 1|Phase 2	24	2013/11/15	
OA	NCT01504464	The Effects of Intra-articular Injection of Mesenchymal Stem Cells in Knee Joint Osteoarthritis	Completed	Intraarticular injection	Auto	BMMSCs	18 Years to 65 Years	Phase 2	40	2012/1/5	
OA	NCT01895413	Autologous Bone Marrow Mesenchymal Stem Cells Transplantation for Articular Cartilage Defects Repair	Completed	Intraarticular injection	Auto	BMMSCs	25 Years to 65 Years	Phase 1|Phase 2	10	2013/7/10	
OA	NCT01626677	Follow-Up Study of CARTISTEM® Versus Microfracture for the Treatment of Knee Articular Cartilage Injury or Defect	Completed	UNS	Allo	UCBMSCs	18 Years and older	Phase 3	103	2012/6/25	
OA	NCT02123368	Treatment of Knee Osteoarthritis by Intra-articular Injection of Bone Marrow Mesenchymal Stem Cells	Completed	Intraarticular injection	Auto	BMMSCs	50 Years to 80 Years	Phase 1|Phase 2	30	2014/4/25	Lamo-Espinosa 2016[120], Lamo-Espinosa 2018[122]
OA	NCT01586312	Treatment of Knee Osteoarthritis With Allogenic Mesenchymal Stem Cells	Completed	Intraarticular injection	Allo	BMMSCs	18 Years to 75 Years	Phase 1|Phase 2	30	2012/4/26	Vega 2015[129], García-Sancho 2017[131]
OA	NCT01585857	ADIPOA - Clinical Study	Completed	Intraarticular injection	Auto	AdMSCs	50 Years to 75 Years	Phase 1	18	2012/4/26	Pers 2016[124]
OA	NCT01453738	Allogeneic Mesenchymal Stem Cells in Osteoarthritis	Completed	Intraarticular injection	Allo	BMMSCs	40 Years to 70 Years	Phase 2	60	2011/10/18	Gupta 2016[130]
OA	NCT01448434	Allogeneic Mesenchymal Stem Cells for Osteoarthritis	Completed	Intraarticular injection	Allo	UNS	20 Years to 70 Years	Phase 2	72	2011/10/7	
OA	NCT01459640	Intra-Articular Autologous Bone Marrow Mesenchymal Stem Cells Transplantation to Treat Mild to Moderate Osteoarthritis	Unknown status	Intraarticular injection	Auto	BMMSCs	18 Years to 70 Years	Phase 2	50	2011/10/25	

OA	NCT01227694	Adult Stem Cell Therapy for Repairing Articular Cartilage in Gonarthrosis	Completed	Intraarticular injection	Auto	BMMSCs	18 Years to 65 Years	Phase 1|Phase 2	15	2010/10/25	
OA	NCT01436058	Side Effects of Autologous Mesenchymal Stem Cell Transplantation in Ankle Joint Osteoartritis	Completed	Intraarticular injection	Auto	BMMSCs	18 Years to 65 Years	Phase 1	6	2011/9/19	Emadedin 2015[119]
OA	NCT01159899	Transplantation of Bone Marrow Stem Cells Stimulated by Proteins Scaffold to Heal Defects Articular Cartilage of the Knee	Unknown status	Intraarticular injection	Auto	BMMSCs	30 Years to 75 Years	Early Phase 1	50	2010/7/12	
OA	NCT01499056	Mesenchymal Stem Cell Transplantation in Osteoarthritis of Hip Joint	Completed	Intraarticular injection	Auto	BMMSCs	18 Years to 65 Years	Phase 1	6	2011/12/26	Emadedin 2015[119]
OA	NCT01183728	Treatment of Knee Osteoarthritis With Autologous Mesenchymal Stem Cells	Completed	Intraarticular injection	Auto	BMMSCs	18 Years to 76 Years	Phase 1|Phase 2	12	2010/8/18	
OA	NCT01207661	Articular Cartilage Resurfacing With Mesenchymal Stem Cells In Osteoarthritis Of Knee Joint	Completed	Intraarticular injection	Auto	BMMSCs	18 Years to 65 Years	Phase 1	6	2010/9/23	Emadedin 2015[119]
OA	NCT01041001	Study to Compare Efficacy and Safety of Cartistem and Microfracture in Patients With Knee Articular Cartilage Injury	Completed	UNS	Allo	UCBMSCs	18 Years and older	Phase 3	104	2009/12/30	
OA	NCT01300598	Autologous Adipose Tissue Derived Mesenchymal Stem Cells Transplantation in Patient With Degenerative Arthritis	Completed	Intraarticular injection	Auto	AdMSCs	18 Years to 75 Years	Phase 1|Phase 2	18	2011/2/21	Jo 2017[125]
OA	NCT00850187	Autologous Transplantation of Mesenchymal Stem Cells (MSCs) and Scaffold in Full-thickness Articular Cartilage	Completed	UNS	Auto	BMMSCs	45 Years to 60 Years	Phase 1	6	2009/2/24	
OA	NCT00891501	The Use of Autologous Bone Marrow Mesenchymal Stem Cells in the Treatment of Articular Cartilage Defects	Unknown status	Intraarticular injection	Auto	BMMSCs	15 Years to 55 Years	Phase 2|Phase 3	25	2009/5/1	
OA	NCT02674399	A Phase 2 Study to Evaluate the Efficacy and Safety of JointStem in Treatment of Osteoarthritis	Completed	UNS	Auto	AdMSCs	22 Years to 60 Years	Phase 2	28	2016/2/4	
OA	NCT02641860	Clinical Trial of Allogenic Adipose Tissue-Derived Mesenchymal Progenitor Cells Therapy for Knee Osteoarthritis	Completed	Intraarticular injection	Allo	AdMSCs	18 Years to 70 Years	Phase 1	22	2015/12/30	Zhao 2019[132]

OA	NCT04368806	A Phase 2b/3a Study to Evaluate the Efficacy and Safety of JointStem in Patients Diagnosed as Knee Osteoarthritis	Not yet recruiting	UNS	Auto	AdMSCs	18 Years and older	Phase 2|Phase 3	140	2020/4/30	
OA	NCT04448106	Autologous Adipose Tissue-Derived Mesenchymal Stem Cells (AdMSCs) for Osteoarthritis	Not yet recruiting	Intraarticular injection/IV	Auto	AdMSCs	18 Years and older	Phase 2	300	2020/6/25	
OA	NCT04351932	Bone Marrow Versus Adipose Autologous Mesenchymal Stem Cells for the Treatment of Knee Osteoarthritis	Not yet recruiting	Intraarticular injection	UNS	BMMSCs/AdMSCs	18 Years to 70 Years	Phase 3	54	2020/4/17	
OA	NCT04339504	Follow-up Safety and Efficacy Evaluation on Subjects Who Completed Phase I Clinical Trial	Recruiting	Intraarticular injection	Allo	UCBMSCs	19 Years and older	Phase 1	12	2020/4/9	
OA	NCT04427930	Follow-up Study for Participants of Jointstem Phase 3 Clinical Trial	Enrolling by invitation	UNS	Auto	AdMSCs	20 Years and older	Phase 3	260	2020/6/11	
OA	NCT04326985	RCT Mesenchymal Stem Cells Versus Hyaluronic Acid in OA Knee):	Completed	Intraarticular injection	Auto	UNS	18 Years to 65 Years	Early Phase 1	20	2020/3/30	
OA	NCT02855073	Clinical Trial to Compare ReJoinTM to Sodium Hyaluronate Injection for Knee Osteoarthritis Cartilage Defects	Active, not recruiting	Intraarticular injection	Auto	AdMSCs	18 Years to 70 Years	Phase 2	28	2016/8/4	Qiao 2020[128]
OA	NCT04453111	Efficacy of Bone-marrow-derived and Placenta-derived Multipotent Mesenchymal Stem / Stromal Cells for Osteoarthritis	Recruiting	Intraarticular injection	Auto/Allo	BMMSCs/PMSCs	18 Years to 75 Years	Phase 1|Phase 2	45	2020/7/1	
OA	NCT04043819	Evaluation of Safety and Exploratory Efficacy of an Autologous Adipose-derived Cell Therapy Product for Treatment of Single Knee Osteoarthritis	Active, not recruiting	Intraarticular injection	Auto	AdMSCs	18 Years to 80 Years	Phase 1	125	2019/8/2	
OA	NCT03007576	Treatment of Knee Osteoarthritis With Autologous Mesenchymal Stromal Cell Product (RegStem)	Completed	Intraarticular injection	Auto	AdMSCs	50 Years to 75 Years	Phase 1	12	2017/1/2	
OA	NCT02805855	Autologous Culture Expanded Mesenchymal Stromal Cells for Knee Osteoarthritis	Recruiting	UNS	Auto	AdMSCs	40 Years to 70 Years	Phase 1	24	2016/6/20	
OA	NCT03943576	Allogeneic Adipose Tissue-Derived Mesenchymal Stem Cells (GXCPC1) for Knee Osteoarthritis	Recruiting	Intraarticular injection	Allo	AdMSCs	40 Years to 80 Years	Phase 1|Phase 2	30	2019/5/9	

OA	NCT04240873	Clinical Study of Intra Articular Injection of Catholic MASTER Cell (Bone Marrow Derived Mesenchymal Stem Cell) in Knee Osteoarthritis	Recruiting	Intraarticular injection	UNS	BMMSCs	20 Years to 80 Years	Phase 1|Phase 2	24	2020/1/27
OA	NCT03164083	The Effects of Stromal Vascular Fraction and Mesenchymal Stem Cells as Intra-articular Injection in Knee Joint Osteoarthritis	Withdrawn	Intraarticular injection	Auto	BMMSCs	25 Years to 65 Years	Phase 2	0	2017/5/23
OA	NCT04037345	Evaluate Safety and Explore Efficacy of SMUP-IA-01 in Patients With Knee Osteoarthritis	Recruiting	Intraarticular injection	Allo	UCBMSCs	19 Years and older	Phase 1	12	2019/7/30
OA	NCT03602872	Safety of Allogeneic Bone Marrow Derived Mesenchymal Stem Cells in Subjects With Osteoarthritis	Withdrawn	Intraarticular injection	Allo	BMMSCs	35 Years to 65 Years	Phase 1	0	2018/7/27
OA	NCT03969680	Mesenchymal Stem Cell Transplantation for Osteoarthritis	Recruiting	Intraarticular injection	Auto	BMMSCs	40 Years to 70 Years	explore the efficacy and safety	60	2019/5/31
OA	NCT03990805	A Phase 3 Study to Evaluate the Efficacy and Safety of JointStem in Treatment of Osteoarthritis	Active, not recruiting	Intraarticular injection	Auto	AdMSCs	20 Years to 100 Years	Phase 3	260	2019/6/19
OA	NCT03869229	Adipose-derived Mesenchymal Stem Cells in Osteoarthritis	Recruiting	Intraarticular injection	Auto	AdMSCs	30 Years to 75 Years	Phase 1|Phase 2	100	2019/3/11
OA	NCT04130100	Clinical Study of Pulp Mesenchymal Stem Cells in the Treatment of Primary Mild to Moderate Knee Osteoarthritis	Recruiting	Intraarticular injection	UNS	DPMSCs	40 Years to 70 Years	Early Phase 1	60	2019/10/17
OA	NCT03818737	Multicenter Trial of Stem Cell Therapy for Osteoarthritis (MILES)	Recruiting	Intraarticular injection	Allo	WJUCMSCs	40 Years to 70 Years	Phase 3	480	2019/1/28
OA	NCT03956719	Treatment of Early Knee Osteoarthritis With Autologous Adipose-derived Mesenchymal Stem Cells	Recruiting	Intraarticular injection	Auto	AdMSCs	15 Years to 65 Years	explore the efficacy and safety	8	2019/5/21
OA	NCT03477942	Impact of Mesenchymal Stem Cells in Knee Osteoarthritis	Recruiting	Intraarticular injection	Auto	BMMSCs	18 Years to 60 Years	Phase 1	16	2018/3/27
OA	NCT03955497	Effectiveness of Autologous Adipose-derived Stem Cells in the Treatment of Knee Cartilage Injury	Recruiting	Intraarticular injection	Auto	AdMSCs	18 Years to 70 Years	Phase 1|Phase 2	30	2019/5/20

OA	NCT03589287	Allogeneic Bone Marrow MSC Therapy for Knee Osteoarthritis	Recruiting	Intraarticular injection	Allo	BMMSCs	40 Years and older	Phase 1|Phase 2	15	2018/7/17
OA	NCT03357575	The Comparison of Efficacy and Safety of the Mesenchymal Stem Cells From Adipose and Hyaluronic Acid	Not yet recruiting	Intraarticular injection	UNS	AdMSCs	18 Years to 75 Years	evaluate the efficacy and safety	14	2017/11/30
OA	NCT03337243	Effect of Implanting Allogenic Cytokines Derived From Human Amniotic Membrane (HAM) and Mesenchymal Stem Cells Derived From Human Umbilical Cord Wharton's Jelly (HUMCWJ) on Pain and Functioning of Knee Osteoarthritis	Completed	Intraarticular injection	Allo	WJUCMSCs/Amniotic MSCs	50 Years to 85 Years	Not Applicable	60	2017/11/8
OA	NCT02963727	Use of Wharton Jelly Derived Mesenchymal Stem Cells for Knee Osteoarthrosis	Recruiting	Intraarticular injection	Allo	WJUCMSCs	42 Years to 75 Years	Phase 1	10	2016/11/15
OA	NCT02966951	Use of Adipose Tissue Derived Mesenchymal Stem Cells for Knee Osteoarthrosis	Recruiting	Intraarticular injection	UNS	AdMSCs	42 Years to 75 Years	Phase 1	10	2016/11/17
OA	NCT03028428	Intra-articular Injection of MSCs in Treatment of Knee OA	Unknown status	Intraarticular injection	Allo	PMSCs	40 Years to 75 Years	Phase 2	1	2017/1/23
OA	NCT03000712	Investigator Initiated Trial to Evaluate the Efficacy and Safety of Intra-articular Injection of ADMSC Comared to Negative Control After High Tibial Osteotomy in the Osteoarthritis Patients.	Active, not recruiting	Intraarticular injection	Auto	AdMSCs	20 Years to 80 Years	evaluate the efficacy and safety	26	2016/12/22
OA	NCT02838069	A Study Evaluating the Efficacy of a Single Injection Autologous Adipose Derived Mesenchymal Stromal Cells in Patients With Knee Osteoarthritis	Recruiting	Intraarticular injection	Auto	AdMSCs	45 Years to 75 Years	Phase 2	153	2016/7/20
OA	NCT02776943	UCMSC Transplantation in the Treatment of Cartilage Damage	Unknown status	Intraarticular injection	Allo	WJUCMSCs	18 Years to 70 Years	Phase 1|Phase 2	20	2016/5/18
OA	NCT04223622	Effects of ASC Secretome on Human Osteochondral Explants	Not yet recruiting	Intraarticular injection	UNS	AdMSC-secretome	18 Years and older	Not Applicable	24	2020/1/10

OA	NCT04212728	Treatment of Knee Osteoarthritis With Autologous Adipose-derived Mesenchymal Stem Cells	Recruiting	Intraarticular injection	Auto	AdMSCs	40 Years to 70 Years	explore the efficacy and safety	60	2019/12/27	
OA	NCT04604288	Individual Patient Expanded Access IND of Autologous HB-asMSCS for the Treatment of Hip Osteoarthritis	No longer available	Intraarticular injection	Auto	AdMSCs	Child, Adult, Older Adult	Not Applicable		2020/10/27	
OA	NCT04208646	Allogenic Adipose Tissue-Derived Mesenchymal Progenitor Cells for the Treatment of Knee Osteoarthritis	Not yet recruiting	Intraarticular injection	Allo	AdMSCs	40 Years to 75 Years	Phase 2	108	2019/12/23	

Table S4: Clinical trials for pulmonary inflammation & COVID-19 using MSC-based products

Conditions	NCT Number	Title	Status	Delivery	Auto/Allo?	Source MSCs	Age	Phases	Enrollment	First Posted	Publications
ARDS	NCT01775774	Human Mesenchymal Stem Cells For Acute Respiratory Distress Syndrome	Completed	IV	Allo	BMMSCs	18 Years and older	Phase 1	9	2013/1/25	Liu 2014[140], Wilson 2015[137]
ARDS	NCT01902082	Adipose-derived Mesenchymal Stem Cells in Acute Respiratory Distress Syndrome	Unknown status	IV	Allo	AdMSCs	18 Years to 90 Years	Phase 1	20	2013/7/18	Zheng 2014[136]
ARDS	NCT02097641	Human Mesenchymal Stromal Cells For Acute Respiratory Distress Syndrome (START)	Completed	IV	Allo	BMMSCs	18 Years and older	Phase 2	60	2014/3/27	Liu 2014[140], Matthay 2019[138]
ARDS	NCT02112500	Mesenchymal Stem Cell in Patients With Acute Severe Respiratory Failure	Unknown status	IV	Auto	BMMSCs	20 Years to 80 Years	Phase 2	10	2014/4/14	
ARDS	NCT02215811	Treatment of Severe Acute Respiratory Distress Syndrome With Allogeneic Bone Marrow-derived Mesenchymal Stromal Cells	Unknown status	UNS	Allo	BMMSCs	18 Years and older	Phase 1	10	2014/8/13	
ARDS	NCT02444455	Human Umbilical-Cord-Derived Mesenchymal Stem Cell Therapy in Acute Lung Injury	Unknown status	IV	Allo	WJUCMSCs	35 Years to 70 Years	Phase 1|Phase 2	20	2015/5/14	
ARDS	NCT02804945	Mesenchymal Stem Cells (MSCs) for Treatment of Acute Respiratory Distress Syndrome (ARD) in Patients With Malignancies	Completed	IV	Allo	BMMSCs	18 Years and older	Phase 1	20	2016/6/17	
ARDS	NCT03608592	Human Umbilical Cord Mesenchymal Stem Cells (MSCs) Therapy in ARDS	Recruiting	IV	Allo	WJUCMSCs	18 Years and older	Phase 1|Phase 2	26	2018/8/1	
ARDS	NCT03818854	Mesenchymal Stromal Cells For Acute Respiratory Distress Syndrome	Recruiting	IV	Allo	BMMSCs	18 Years and older	Phase 2	120	2019/1/28	

ARDS	NCT04282928	Efficacy and Safety of Umbilical Cord Mesenchymal Stem Cells for the Treatment of Severe Viral Pneumonian	Not yet recruiting	IV	Allo	WJUCMSCs	18 Years to 75 Years	Phase 1	40	2020/2/25	
ARDS	NCT04289194	Clinical Study to Assess the Safety and Preliminary Efficacy of HCR040 in Acute Respiratory Distress Syndrome	Active, not recruiting	IV	Allo	AdMSCs	18 Years and older	Phase 1|Phase 2	26	2020/2/28	
ARDS	NCT04347967	Mesenchymal Stem Cells for The Treatment of Acute Respiratory Distress Syndrome (ARDS)	Not yet recruiting	IV	Allo	WJUCMSCs	20 Years to 85 Years	Phase 1	18	2020/4/15	
ARDS	NCT04544215	A Clinical Study of Mesenchymal Progenitor Cell Exosomes Nebulizer for the Treatment of Pulmonary Infection	Recruiting	Aerosol inhalation	Allo	AdMSC-exosomes	18 Years to 75 Years	Phase 1|Phase 2	60	2020/9/10	
ARDS	NCT04602104	A Clinical Study of Mesenchymal Stem Cell Exosomes Nebulizer for the Treatment of ARDS	Not yet recruiting	Aerosol Inhalation	Allo	UNS-MSC-Exo	18 Years to 70 Years	Phase 1|Phase 2	169	2020/10/26	
Asthma	NCT02192736	Safety and Feasibility Study of Intranasal Mesenchymal Trophic Factor (MTF) for Treatment of Asthma	Active, not recruiting	Intra-nasal	Allo	WJUCMSCs	21 Years to 60 Years	Phase 1|Phase 2	20	2014/7/17	
Asthma	NCT03137199	Allogeneic Human Cells (hMSC) Via Intravenous Delivery in Patients With Mild Asthma	Terminated	IV	Allo	BMMSCs	18 Years to 65 Years	Phase 1	3	2017/5/2	
COPD	NCT00683722	PROCHYMAL??(Human Adult Stem Cells) for the Treatment of Moderate to Severe Chronic Obstructive Pulmonary Disease (COPD)	Completed	IV	Allo	BMMSCs	40 Years to 80 Years	Phase 2	62	2008/5/23	Weiss 2013[141]
COPD	NCT01306513	Safety and Feasibility Study of Administration of Mesenchymal Stemcells for Treatment of Emphysema	Completed	IV	Auto	BMMSCs	40 Years to 65 Years	Phase 1	10	2011/3/2	Stolk 2016[142]
COPD	NCT01758055	Safety Study of Endobronchial Transplantation of Autologous Mesenchymal Stem Cells (MSCs) in Emphysema Patients	Unknown status	IT	Auto	BMMSCs	16 Years to 70 Years	Phase 1	12	2012/12/31	
COPD	NCT01849159	Clinical Study of the Efficacy and Safety of the Application of Allogeneic Mesenchymal (Stromal) Cells of Bone Marrow, Cultured Under the Hypoxia in the Treatment of Patients With Severe Pulmonary Emphysema	Withdrawn	IV	Allo	BMMSCs	35 Years to 75 Years	Phase 1|Phase 2	0	2013/5/8	
COPD	NCT01872624	Safety Study of Bone-marrow Derived Mesenchymal Stromal Cells Associated With Endobronchial Valves in Emphysema	Completed	IT	UNS	BMMSCs	18 Years and older	Phase 1	10	2013/6/7	
COPD	NCT02645305	Adipose Derived Stem Cells Transplantation for Chronic Obstructive Pulmonary Disease	Unknown status	IV	Auto	AdMSCs	40 Years to 80 Years	Phase 1|Phase 2	20	2016/1/1	

COPD	NCT02749448	Mesenchymal Stem Cells Therapy for Treatment of Airway Remodeling in Mustard Patients	Unknown status	UNS	Auto	AdMSCs	45 Years to 65 Years	Phase 1	10	2016/4/25	Marzouni 2020[143]
COPD	NCT04018729	Cell Therapy Associated With Endobronchial Valve	Not yet recruiting	IT	UNS	BMMSCs	18 Years and older	Phase 2|Phase 3	34	2019/7/12	
COPD	NCT04206007	Mesenchymal Stem Cells for The Treatment of Chronic Obstructive Pulmonary Disease	Recruiting	IV	Allo	WJUCMSCs	40 Years to 75 Years	Phase 1	9	2019/12/20	
COPD	NCT04433104	Umbilical Cord Mesenchymal Stem Cells Transplantation in the Treatment of Chronic Obstructive Pulmonary Disease	Recruiting	IV	Allo	WJUCMSCs	40 Years and older	Phase 1|Phase 2	40	2020/6/16	
COPD	NCT04047810	Mesenchymal Stem Cells in the Treatment of Subjects With Advance Chronic Obstructive Pulmonary Disease (COPD)	Recruiting	IV	UNS	UNS	18 Years to 90 Years	Phase 1	15	2019/8/7	
COVID-19	NCT03042143	Repair of Acute Respiratory Distress Syndrome by Stromal Cell Administration (REALIST) (COVID-19)	Recruiting	IV	Allo	WJUCMSCs	16 Years and older	Phase 1|Phase 2	75	2017/2/3	Gorman 2020[146]
COVID-19	NCT04252118	Mesenchymal Stem Cell Treatment for Pneumonia Patients Infected With COVID-19	Recruiting	IV	UNS	Unspecified	18 Years to 70 Years	Phase 1	20	2020/2/5	
COVID-19	NCT04269525	Umbilical Cord(UC)-Derived Mesenchymal Stem Cells(MSCs) Treatment for the 2019-novel Coronavirus(nCOV) Pneumonia	Recruiting	IV	Allo	WJUCMSCs	18 Years to 80 Years	Phase 2	16	2020/2/13	
COVID-19	NCT04273646	Study of Human Umbilical Cord Mesenchymal Stem Cells in the Treatment of Severe COVID-19	Not yet recruiting	IV	Allo	WJUCMSCs	18 Years to 65 Years	Not Applicable	48	2020/2/18	
COVID-19	NCT04288102	Treatment With Human Umbilical Cord-derived Mesenchymal Stem Cells for Severe Corona Virus Disease 2019 (COVID-19)	Completed	IV	Allo	WJUCMSCs	18 Years to 75 Years	Phase 2	100	2020/2/28	
COVID-19	NCT04315987	NestaCell簀 Mesenchymal Stem Cell to Treat Patients With Severe COVID-19 Pneumonia	Not yet recruiting	IV	UNS	UNS	18 Years and older	Phase 2	90	2020/3/20	
COVID-19	NCT04331613	Safety and Efficacy of CAStem for Severe COVID-19 Associated With/Without ARDS	Recruiting	IV	Allo	ESC-MSCs	18 Years to 70 Years	Phase 1|Phase 2	9	2020/4/2	
COVID-19	NCT04333368	Cell Therapy Using Umbilical Cord-derived Mesenchymal Stromal Cells in SARS-CoV-2-related ARDS	Recruiting	IV	Allo	WJUCMSCs	18 Years and older	Phase 1|Phase 2	40	2020/4/3	
COVID-19	NCT04336254	Safety and Efficacy Study of Allogeneic Human Dental Pulp Mesenchymal Stem Cells to Treat Severe COVID-19 Patients	Recruiting	IV	Allo	DPMSCs	18 Years to 65 Years	Phase 1|Phase 2	20	2020/4/7	Ye 2020[147]

COVID-19	NCT04339660	Clinical Research of Human Mesenchymal Stem Cells in the Treatment of COVID-19 Pneumonia	Recruiting	IV	Allo	WJUCMSCs	18 Years to 75 Years	Phase 1|Phase 2	30	2020/4/9
COVID-19	NCT04341610	ASC Therapy for Patients With Severe Respiratory COVID-19	Withdrawn	UNS	Allo	AdMSCs	18 Years to 80 Years	Phase 1|Phase 2	0	2020/4/10
COVID-19	NCT04345601	Mesenchymal Stromal Cells for the Treatment of SARS-CoV-2 Induced Acute Respiratory Failure (COVID-19 Disease)	Not yet recruiting	IV	Allo	BMMSCs	18 Years and older	Early Phase 1	30	2020/4/14
COVID-19	NCT04346368	Bone Marrow-Derived Mesenchymal Stem Cell Treatment for Severe Patients With Coronavirus Disease 2019 (COVID-19)	Not yet recruiting	IV	UNS	BMMSCs	18 Years to 75 Years	Phase 1|Phase 2	20	2020/4/15
COVID-19	NCT04348435	A Randomized, Double-Blind, Placebo-Controlled Clinical Trial to Determine the Safety and Efficacy of Hope Biosciences Allogeneic Mesenchymal Stem Cell Therapy (HB-adMSCs) to Provide Protection Against COVID-19	Enrolling by invitation	IV	Allo	AdMSCs	Child, Adult, Older Adult	Phase 2	100	2020/4/16
COVID-19	NCT04348461	BAttLe Against COVID-19 Using MesenchYmal Stromal Cells	Not yet recruiting	IV	Allo	AdMSCs	18 Years and older	Phase 2	100	2020/4/16
COVID-19	NCT04349631	A Clinical Trial to Determine the Safety and Efficacy of Hope Biosciences Autologous Mesenchymal Stem Cell Therapy (HB-adMSCs) to Provide Protection Against COVID-19	Enrolling by invitation	IV	Auto	AdMSCs	Child, Adult, Older Adult	Phase 2	56	2020/4/16
COVID-19	NCT04352803	Adipose Mesenchymal Cells for Abatement of SARS-CoV-2 Respiratory Compromise in COVID-19 Disease	Not yet recruiting	IV	Auto	AdMSCs	18 Years to 90 Years	Phase 1	20	2020/4/20
COVID-19	NCT04355728	Use of UC-MSCs for COVID-19 Patients	Active, not recruiting	IV	Allo	WJUCMSCs	18 Years and older	Phase 1|Phase 2	24	2020/4/21
COVID-19	NCT04361942	Treatment of Severe COVID-19 Pneumonia With Allogeneic Mesenchymal Stromal Cells (COVID_MSV)	Recruiting	IV	Allo	UNS	18 Years and older	Phase 2	24	2020/4/24
COVID-19	NCT04362189	Efficacy and Safety Study of Allogeneic HB-adMSCs for the Treatment of COVID-19	Active, not recruiting	IV	Allo	AdMSCs	Child, Adult, Older Adult	Phase 2	100	2020/4/24
COVID-19	NCT04366063	Mesenchymal Stem Cell Therapy for SARS-CoV-2-related Acute Respiratory Distress Syndrome	Recruiting	IV	UNS	UNS-MSC-Evs	18 Years to 65 Years	Phase 2|Phase 3	60	2020/4/28
COVID-19	NCT04366271	Clinical Trial of Allogeneic Mesenchymal Cells From Umbilical Cord Tissue in Patients With COVID-19	Recruiting	IV	Allo	WJUCMSCs	40 Years to 80 Years	Phase 2	106	2020/4/28

COVID-19	NCT04366323	Clinical Trial to Assess the Safety and Efficacy of Intravenous Administration of Allogeneic Adult Mesenchymal Stem Cells of Expanded Adipose Tissue in Patients With Severe Pneumonia Due to COVID-19	Recruiting	IV	Allo	AdMSCs	18 Years to 80 Years	Phase 1|Phase 2	26	2020/4/28
COVID-19	NCT04366830	Intermediate-size Expanded Access Program (EAP), Mesenchymal Stromal Cells (MSC) for Acute Respiratory Distress Syndrome (ARDS) Due to COVID-19 Infection	No longer available	IV	Allo	BMMSCs	18 Years and older	Phase 1|Phase 2		2020/4/29
COVID-19	NCT04367077	MultiStem Administration for COVID-19 Induced ARDS (MACoVIA)	Recruiting	IV	Allo	BMMSCs	18 Years to 89 Years	Phase 2|Phase 3	400	2020/4/29
COVID-19	NCT04371393	MSCs in COVID-19 ARDS	Recruiting	IV	Allo	BMMSCs	18 Years and older	Phase 3	300	2020/5/1
COVID-19	NCT04371601	Safety and Effectiveness of Mesenchymal Stem Cells in the Treatment of Pneumonia of Coronavirus Disease 2019	Active, not recruiting	IV	Allo	WJUCMSCs	18 Years to 70 Years	Early Phase 1	60	2020/5/1
COVID-19	NCT04377334	Mesenchymal Stem Cells (MSCs) in Inflammation-Resolution Programs of Coronavirus Disease 2019 (COVID-19) Induced Acute Respiratory Distress Syndrome (ARDS)	Not yet recruiting	IV	Allo	BMMSCs	18 Years and older	Phase 2	40	2020/5/6
COVID-19	NCT04382547	Treatment of Covid-19 Associated Pneumonia With Allogenic Pooled Olfactory Mucosa-derived Mesenchymal Stem Cells	Enrolling by invitation	IV	Allo	OMMSCs	18 Years to 70 Years	Phase 1|Phase 2	40	2020/5/11
COVID-19	NCT04389450	Double-Blind, Multicenter, Study to Evaluate the Efficacy of PLX PAD for the Treatment of COVID-19	Recruiting	IM	Allo	PMSCs	40 Years to 80 Years	Phase 2	140	2020/5/15
COVID-19	NCT04390139	Efficacy and Safety Evaluation of Mesenchymal Stem Cells for the Treatment of Patients With Respiratory Distress Due to COVID-19	Recruiting	IV	Allo	WJUCMSCs	18 Years to 75 Years	Phase 1|Phase 2	30	2020/5/15
COVID-19	NCT04390152	Safety and Efficacy of Intravenous Wharton's Jelly Derived Mesenchymal Stem Cells in Acute Respiratory Distress Syndrome Due to COVID 19	Not yet recruiting	IV	Allo	WJUCMSCs	18 Years to 80 Years	Phase 1|Phase 2	40	2020/5/15
COVID-19	NCT04392778	Clinical Use of Stem Cells for the Treatment of Covid-19	Recruiting	IV	Alo	UNS	40 Years to 60 Years	Phase 1|Phase 2	30	2020/5/19

COVID-19	NCT04397796	Study of the Safety of Therapeutic Tx With Immunomodulatory MSC in Adults With COVID-19 Infection Requiring Mechanical Ventilation	Recruiting	IV	Allo	BMMSCs	18 Years to 80 Years	Phase 1	45	2020/5/21
COVID-19	NCT04398303	ACT-20 in Patients With Severe COVID-19 Pneumonia	Not yet recruiting	IV	Allo	WJUCMSCs/MSC-CM	18 Years to 85 Years	Phase 1|Phase 2	70	2020/5/21
COVID-19	NCT04399889	hCT-MSCs for COVID19 ARDS	Recruiting	IV	Allo	WJUCMSCs	18 Years and older	Phase 1|Phase 2	30	2020/5/22
COVID-19	NCT04400032	Cellular Immuno-Therapy for COVID-19 Acute Respiratory Distress Syndrome - Vanguard	Not yet recruiting	IV	Allo	BMMSCs	18 Years and older	Phase 1	9	2020/5/22
COVID-19	NCT04416139	Mesenchymal Stem Cell for Acute Respiratory Distress Syndrome Due for COVID-19	Recruiting	IV	Allo	WJUCMSCs	18 Years and older	Phase 2	10	2020/6/4
COVID-19	NCT04428801	Autologous Adipose-derived Stem Cells (AdMSCs) for COVID-19	Not yet recruiting	IV	Auto	AdMSCs	18 Years and older	Phase 2	200	2020/6/11
COVID-19	NCT04429763	Safety and Efficacy of Mesenchymal Stem Cells in the Management of Severe COVID-19 Pneumonia	Not yet recruiting	UNS	Allo	WJUCMSCs	18 Years to 79 Years	Phase 2	30	2020/6/12
COVID-19	NCT04444271	Mesenchymal Stem Cell Infusion for COVID-19 Infection	Recruiting	IV	Allo	BMMSCs	10 Years and older	Phase 2	20	2020/6/23
COVID-19	NCT04445454	Mesenchymal Stromal Cell Therapy for Severe Covid-19 Infection	Recruiting	IV	Allo	BMMSCs	18 Years to 70 Years	Phase 1|Phase 2	20	2020/6/24
COVID-19	NCT04447833	Mesenchymal Stromal Cell Therapy For The Treatment Of Acute Respiratory Distress Syndrome	Recruiting	IV	Alo	BMMSCs	18 Years to 65 Years	Phase 1	9	2020/6/25
COVID-19	NCT04452097	Use of hUC-MSC Product (BX-U001) for the Treatment of COVID-19 With ARDS	Not yet recruiting	IV	Allo	WJUCMSCs	18 Years to 80 Years	Phase 1|Phase 2	39	2020/6/30
COVID-19	NCT04456361	Use of Mesenchymal Stem Cells in Acute Respiratory Distress Syndrome Caused by COVID-19	Active, not recruiting	IV	Allo	WJUCMSCs	18 Years and older	Early Phase 1	9	2020/7/2
COVID-19	NCT04456439	Intermediate-size Expanded Access Program (EAP), Mesenchymal Stromal Cells (MSC) for Multisystem Inflammatory Syndrome in Children (MIS-C) Associated With Coronavirus Disease (COVID-19)	Available	IV	Allo	BMMSCs	2 Months to 17 Years	assess the safety and efficacy		2020/7/2

COVID-19	NCT04457609	Administration of Allogenic UC-MSCs as Adjuvant Therapy for Critically-Ill COVID-19 Patients	Recruiting	IV	Allo	WJUCMSCs	18 Years to 95 Years	Phase 1	40	2020/7/7
COVID-19	NCT04461925	Treatment of Coronavirus COVID-19 Pneumonia (Pathogen SARS-CoV-2) With Cryopreserved Allogeneic P_MMSCs and UC-MMSCs	Recruiting	IV	Allo	PMSCs	18 Years to 75 Years	Phase 1|Phase 2	30	2020/7/8
COVID-19	NCT04466098	Multiple Dosing of Mesenchymal Stromal Cells in Patients With ARDS (COVID-19)	Recruiting	UNS	UNS	UNS	18 Years to 80 Years	Phase 2	30	2020/7/10
COVID-19	NCT04467047	Safety and Feasibility of Allogenic MSC in the Treatment of COVID-19	Not yet recruiting	IV	Allo	UNS	Child, Adult, Older Adult	Phase 1	10	2020/7/10
COVID-19	NCT04490486	Umbilical Cord Tissue (UC) Derived Mesenchymal Stem Cells (MSCs) Versus Placebo to Treat Acute Pulmonary Inflammation Due to COVID-19	Not yet recruiting	IV	Allo	WJUCMSCs	18 Years and older	Phase 1	21	2020/7/29
COVID-19	NCT04492501	Investigational Treatments for COVID-19 in Tertiary Care Hospital of Pakistan	Completed	UNS	UNS	BMMSCs	18 Years to 90 Years	Not Applicable	600	2020/7/30
COVID-19	NCT04525378	MSC-based Therapy in COVID-19-associated Acute Respiratory Distress Syndrome	Recruiting	IV	UNS	UNS	18 Years and older	Phase 1	20	2020/8/25
COVID-19	NCT04527224	Study to Evaluate the Efficacy and Safety of AstroStem-V in Treatment of COVID-19 Pneumonia	Not yet recruiting	UNS	Allo	AdMSCs	19 Years to 80 Years	Phase 1|Phase 2	10	2020/8/26
COVID-19	NCT04535856	Therapeutic Study to Evaluate the Safety and Efficacy of DW-MSC in COVID-19 Patients	Not yet recruiting	IV	Allo	UNS	19 Years and older	Phase 1	9	2020/9/2
COVID-19	NCT04537351	The MEseNchymal coviD-19 Trial: a Pilot Study to Investigate Early Efficacy of MSCs in Adults With COVID-19	Recruiting	IV	Allo	iPSC-MSC	18 Years and older	Phase 1|Phase 2	24	2020/9/3
COVID-19	NCT04276987	A Pilot Clinical Study on Inhalation of Mesenchymal Stem Cells Exosomes Treating Severe Novel Coronavirus Pneumonia	Completed	Aerosol inhalation	Allo	AdMSC-exosomes	18 Years to 75 Years	Phase 1	24	2020/2/19
COVID-19	NCT04293692	Therapy for Pneumonia Patients iInfected by 2019 Novel Coronavirus	Withdrawn	IV	Allo	WJUCMSCs	18 Years to 75 Years	Phase 1|Phase 2	0	2020/3/3
COVID-19	NCT04302519	Novel Coronavirus Induced Severe Pneumonia Treated by Dental Pulp Mesenchymal Stem Cells	Not yet recruiting	IV	UNS	DPMSCs	18 Years to 75 Years	Early Phase 1	24	2020/3/10
COVID-19	NCT04313322	Treatment of COVID-19 Patients Using Wharton's Jelly-Mesenchymal Stem Cells	Recruiting	IV	Allo	WJUCMSCs	18 Years and older	Phase 1	5	2020/3/18

COVID-19	NCT04437823	Efficacy of Intravenous Infusions of Stem Cells in the Treatment of COVID-19 Patients	Recruiting	IV	Allo	WJUCMSCs	30 Years to 70 Years	Phase 2	20	2020/6/18	
COVID-19	NCT04629105	Regenerative Medicine for COVID-19 and Flu-Elicited ARDS Using Longeveron Mesenchymal Stem Cells (LMSCs) (RECOVER)	Recruiting	IV	Allo	BMMSCs	18 Years and older	Phase 1	70	2020/11/16	
COVID-19	NCT04625738	Efficacy of Infusions of MSC From Wharton Jelly in the SARS-Cov-2 (COVID-19) Related Acute Respiratory Distress Syndrome	Not yet recruiting	IV	Allo	WJUCMSCs	18 Years and older	Phase 2	30	2020/11/12	
COVID-19	NCT04611256	Mesenchymal Stem Cells in Patients Diagnosed With COVID-19	Recruiting	IV	UNS	AdMSCs	18 Years to 65 Years	Phase 1	20	2020/11/2	
COVID-19	NCT04573270	Mesenchymal Stem Cells for the Treatment of COVID-19	Completed	IV	Allo	WJUCMSCs	18 Years and older	Phase 1	40	2020/10/5	
COVID-19	NCT04565665	Cord Blood-Derived Mesenchymal Stem Cells for the Treatment of COVID-19 Related Acute Respiratory Distress Syndrome	Recruiting	IV	Allo	UCBMSCs	18 Years and older	Phase 1	70	2020/9/25	
COVID-19	NCT04615429	Clinical Trial to Assess the Efficacy of MSC in Patients With ARDS Due to COVID-19	Recruiting	IV	Allo	BMMSCs	18 Years and older	Phase 2	20	2020/11/4	Payares-Herrera 2021[139]
COVID-19	NCT04753476	Treatment of Severe COVID-19 Patients Using Secretome of Hypoxia-Mesenchymal Stem Cells in Indonesia	Recruiting	IM	UNS	UNS	Child, Adult, Older Adult	Phase 2	48	2021/2/15	
COVID-19	NCT04657458	Expanded Access Protocol on Bone Marrow Mesenchymal Stem Cell Derived Extracellular Vesicle Infusion Treatment for Patients With COVID-19 Associated ARDS	Available	IV	UNS	BMMSCs	18 Years and older	Not Applicable		2020/12/8	
COVID-19	NCT04728698	Study of Intravenous Administration of Allogeneic Adipose-Derived Mesenchymal Stem Cells for COVID-19-Induced Acute Respiratory Distress	Not yet recruiting	IV	Allo	AdMSCs	18 Years and older ?? (Adult, Older Adult)	Phase 2	100	2021/1/28	
COVID-19	NCT04713878	Mesenchymal Stem Cells Therapy in Patients With COVID-19 Pneumonia	Completed	IV	UNS	UNS	18 Years to 90 Years	Not Applicable	21	2021/1/19	
COVID-19	NCT04780685	A Phase II Study in Patients With Moderate to Severe ARDS Due to COVID-19	Recruiting	IV	Allo	UNS	18 Years and older	Phase 2	40	2021/3/3	
COVID-19	NCT04798066	Intermediate Size Expanded Access Protocol Evaluating HB-adMSC's for the Treatment of Post-COVID-19 Syndrome	No longer available	IV	Auto	AdMSCs	18 Years to 65 Years	Phase 1|Phase 2		2021/3/15	

COVID-19	NCT04798716	The Use of Exosomes for the Treatment of Acute Respiratory Distress Syndrome or Novel Coronavirus Pneumonia Caused by COVID-19	Not yet recruiting	IV	UNS	UNS-MSC-Exo	18 Years and older	Phase 1|Phase 2	55	2021/3/15	
IPF	NCT01385644	A Study to Evaluate the Potential Role of Mesenchymal Stem Cells in the Treatment of Idiopathic Pulmonary Fibrosis	Completed	IV	Allo	PMSCs	40 Years to 80 Years	Phase 1	8	2011/6/30	
IPF	NCT01919827	Study of Autologous Mesenchymal Stem Cells to Treat Idiopathic Pulmonary Fibrosis	Completed	IT	Auto	BMMSCs	30 Years to 80 Years	Phase 1	17	2013/8/9	
IPF	NCT02013700	Allogeneic Human Cells (hMSC)in Patients With Idiopathic Pulmonary Fibrosis Via Intravenous Delivery (AETHER)	Completed	IV	Allo	BMMSCs	40 Years to 90 Years	Phase 1	9	2013/12/17	Glassberg 2017[144]
IPF	NCT02135380	Evaluate Safety and Efficacy of Intravenous Autologous ADMSc for Treatment of Idiopathic Pulmonary Fibrosis	Unknown status	IV	Auto	AdMSCs	30 Years to 70 Years	Phase 1|Phase 2	60	2014/5/9	
IPF	NCT02594839	Safety and Efficacy of Allogeneic Mesenchymal Stem Cells in Patients With Rapidly Progressive Interstitial Lung Disease	Completed	IV	Allo	BMMSCs	20 Years to 80 Years	Phase 1|Phase 2	20	2015/11/3	
Allograft dysfunction	NCT02709343	Trial of Bone-marrow Derived Mesenchymal Stromal Cells (MSC) for New Onset Chronic Lung Allograft Dysfunction	Recruiting	IV	Allo	BMMSCs	18 Years and older	Phase 2	82	2016/3/16	
Bronchiectasis	NCT02625246	Safety and Potential Efficacy of Human Mesenchymal Stem Cells in Non-Cystic Fibrosis Bronchiectasis	Completed	IV	Allo	BMMSCs	30 Years to 87 Years	Phase 1	6	2015/12/9	
Cystic Fibrosis	NCT02866721	Safety and Tolerability Study of Allogeneic Mesenchymal Stem Cell Infusion in Adults With Cystic Fibrosis	Active, not recruiting	IV	Allo	BMMSCs	18 Years and older	Phase 1	14	2016/8/15	
Cystic Fibrosis	NCT03058068	Human MesenchymAl Stem Cells Infusion in Patients With Cystic Fibrosis	Withdrawn	IV	Allo	UNS	20 Years to 45 Years	Phase 1	0	2017/2/20	
Idiopathic Pulmonary Hemosiderosis	NCT02985346	Efficacy of Bone Marrow Mesenchymal Stem Cell in Pulmonary Hemosiderosis	Not yet recruiting	UNS	UNS	BMMSCs	1 Month to 18 Years	Early Phase 1	100	2016/12/7	
Interstitial Lung Disease	NCT03929120	Allogeneic Bone Marrow Mesenchymal Stem Cells for Patients With Interstitial Lung Disease (ILD) & Connective Tissue Disorders (CTD)	Recruiting	IV	Allo	BMMSCs	18 Years to 80 Years	Phase 1	10	2019/4/26	
Interstitial Pulmonary Fibrosis	NCT03187431	Role of Stem Cell Therapy in Interstitial Pulmonary Fibrosis	Unknown status	IV	Auto	BMMSCs	18 Years to 65 Years	Phase 1	12	2017/6/15	

Lung injury	NCT02444858	Human Umbilical-Cord-Derived Mesenchymal Stem Cell Therapy in Paraquat Poisoning Induced Lung Injury	Unknown status	IV	Allo	WJUCMSCs	15 Years to 60 Years	Phase 1|Phase 2	40	2015/5/15	
Lung injury test in HD	NCT04313647	A Tolerance Clinical Study on Aerosol Inhalation of Mesenchymal Stem Cells Exosomes In Healthy Volunteers	Recruiting	Aerosol inhalation	Allo	AdMSC-exosomes	18 Years to 45 Years	Phase 1	27	2020/3/18	
Obliterative Bronchiolitis	NCT01175655	A Study to Evaluate the Potential of Mesenchymal Stromal Cells to Treat Obliterative Bronchiolitis After Lung Transplantation	Completed	IV	Allo	UNS	Child, Adult, Older Adult	Phase 1	10	2010/8/5	
Obliterative Bronchiolitis	NCT02181712	Mesenchymal Stem Cell Therapy for Lung Rejection	Recruiting	IV	Allo	BMMSCs	18 Years to 75 Years	Phase 1	19	2014/7/4	
Obliterative Bronchiolitis	NCT02543073	MSC for Treatment of Interstitial Lung Disease After Allo-HSCT	Unknown status	IV	Allo	BMMSCs	18 Years to 65 Years	Phase 1|Phase 2	60	2015/9/7	Chen 2019[145]
Pneumoconiosis	NCT02668068	A Study on Pneumoconiosis Treated With Whole-lung Lavage Combined With Mesenchymal Stem Cells	Completed	Bronchial lavage	Allo	WJUCMSCs	18 Years to 70 Years	Phase 1	80	2016/1/29	
Pneumoconiosis	NCT02790762	Human Umbilical Cord-Mesenchymal Stem Cells for Pneumoconiosis	Unknown status	IV	Allo	WJUCMSCs	18 Years to 75 Years	Phase 1	10	2016/6/6	
Radiation-induced Pulmonary Fibrosis	NCT02277145	A Study on Radiation-induced Pulmonary Fibrosis Treated With Clinical Grade Umbilical Cord Mesenchymal Stem Cells	Completed	IT	Allo	WJUCMSCs	18 Years to 70 Years	Phase 1	10	2014/10/28	

Table S5: Clinical trials for liver cirrhosis using MSC-based products

Conditions	NCT Number	Title	Status	Delivery	Auto/Allo?	Source MSCs	Age	Phases	Enrollment	First Posted	Publications
Cirrhosis	NCT00476060	Mesenchymal Stem Cell Transplantation in Decompensated Cirrhosis	Unknown status	IV	Auto	BMMSCs	18 Years to 65 Years	Phase 2	36	2007/5/21	
Cirrhosis	NCT00976287	Autologous Bone Marrow Mesenchymal Stem Cells Transplantation Via Hepatic Artery in Patients With Liver Cirrhosis	Unknown status	Hepatic artery	Auto	BMMSCs	18 Years to 70 Years	Phase 2	50	2009/9/14	
Cirrhosis	NCT00993941	Bone Mesenchymal Stem Cell (BMSC) Transplantation in Liver Cirrhosis Via Portal Vein	Unknown status	IV	Auto	BMMSCs	18 Years to 60 Years	Phase 2	60	2009/10/14	
Cirrhosis	NCT01220492	Umbilical Cord Mesenchymal Stem Cells for Patients With Liver Cirrhosis	Completed	IV	Allo	WJUCMSCs	18 Years to 65 Years	Phase 1|Phase 2	266	2010/10/14	

Cirrhosis	NCT01223664	Allogenic Bone Marrow Stem Cells Transplantation in Patients With Liver Cirrhosis	Unknown status	Portal vein/ Hepatic artery	Allo	BMMSCs	16 Years to 65 Years	Phase 2	60	2010/10/19	
Cirrhosis	NCT01224327	Umbilical Cord Mesenchymal Stem Cells Infusion Via Hepatic Artery in Cirrhosis Patients	Unknown status	Hepatic artery	Allo	WJUCMSCs	18 Years to 70 Years	Phase 1|Phase 2	50	2010/10/20	
Cirrhosis	NCT01233102	Mesenchymal Stem Cells Treat Liver Cirrhosis	Suspended	IV/Hepatic artery	Allo	WJUCMSCs	18 Years to 70 Years	Phase 1|Phase 2	200	2010/11/3	
Cirrhosis	NCT01342250	Human Umbilical Cord Mesenchymal Stem Cells Transplantation for Patients With Decompensated Liver Cirrhosis	Completed	UNS	Allo	WJUCMSCs	18 Years to 70 Years	Phase 1|Phase 2	20	2011/4/27	
Cirrhosis	NCT01454336	Transplantation of Autologous Mesenchymal Stem Cell in Decompensate Cirrhotic Patients With Pioglitazone	Completed	Portal vein	Auto	BMMSCs	18 Years to 65 Years	Phase 1	3	2011/10/19	
Cirrhosis	NCT01483248	Human Menstrual Blood-derived Mesenchymal Stem Cells for Patients With Liver Cirrhosis	Unknown status	IV	Auto	MBMSCs	20 Years to 50 Years	Phase 1|Phase 2	50	2011/12/1	
Cirrhosis	NCT01499459	Autologous Mesenchymal Stem Cell Transplantation in Liver Cirrhosis	Unknown status	IV	Auto	BMMSCs	18 Years to 80 Years	Phase 2	25	2011/12/26	
Cirrhosis	NCT01573923	Safety and Efficacy Study of Umbilical Mesenchymal Stem Cells for Liver Cirrhosis	Unknown status	IV	Allo	WJUCMSCs	30 Years to 60 Years	Phase 1|Phase 2	320	2012/4/10	
Cirrhosis	NCT01591200	Dose Finding Study to Assess Safety and Efficacy of Stem Cells in Liver Cirrhosis	Completed	Hepatic artery	Allo	BMMSCs	18 Years to 65 Years	Phase 2	40	2012/5/3	
Cirrhosis	NCT01741090	The Effectiveness and Safety for Mesenchymal Stem Cell for Alcoholic Liver Cirrhosis	Unknown status	UNS	Auto	BMMSCs	20 Years to 60 Years	Phase 2	12	2012/12/4	Jang 2014[158]
Cirrhosis	NCT01854125	Autologous Mesenchymal Stem Cell Transplantation in Cirrhosis Patients With Refractory Ascites	Unknown status	Hepatic artery	Auto	BMMSCs	18 Years to 65 Years	Phase 3	30	2013/5/15	
Cirrhosis	NCT02327832	Safety Study of Liver Regeneration Therapy Using Cultured Autologous BMSCs	Unknown status	IV	Auto	BMMSCs	20 Years to 75 Years	Phase 1	10	2014/12/30	
Cirrhosis	NCT02652351	Human Umbilical Cord-Mesenchymal Stem Cells for Hepatic Cirrhosis	Unknown status	UNS	Allo	WJUCMSCs	18 Years to 80 Years	Phase 1	20	2016/1/11	
Cirrhosis	NCT02943889	Stem Cell Transplantation in Cirrhotic Patients	Unknown status	Portal vein	Auto	BMMSCs	Child, Adult, Older Adult	Phase 1|Phase 2	40	2016/10/25	

Cirrhosis	NCT03460795	Safety and Efficacy Study of Co-transfering of Mesenchymal Stem Cell and Regulatory T Cells in Treating End-stage Liver Disease	Not yet recruiting	UNS	UNS	UNS	18 Years to 65 Years	Phase 1|Phase 2	30	2018/3/9
Cirrhosis	NCT03529136	Clinical Trial of Umbilical Cord Mesenchymal Stem Cell Transfusion in Decompensated Liver Cirrhosis	Unknown status	IV	Allo	WJUCMSCs	18 Years to 65 Years	Phase 2	252	2018/5/18
Cirrhosis	NCT03626090	Mesenchymal Stem Cell Therapy for Liver Cirrhosis	Recruiting	IV	Auto	BMMSCs	21 Years to 69 Years	Phase 1|Phase 2	20	2018/8/10
Cirrhosis	NCT03945487	Mesenchymal Stem Cells Treatment for Decompensated Liver Cirrhosis	Recruiting	IV	Allo	WJUCMSCs	18 Years to 69 Years	Phase 2	200	2019/5/10
Cirrhosis	NCT04243681	Combination of Autologous MSC and HSC Infusion in Patients With Decompensated Cirrhosis	Recruiting	UNS	Auto	UNS	20 Years to 70 Years	Phase 4	5	2020/1/28
Cirrhosis	NCT03838250	Study to Evaluate Hepatic Artery Injection of Autologous Human Bone Marrow-Derived MSCs in Patients With Alcoholic LC	Recruiting	Hepatic artery	Auto	BMMSCs	18 Years to 70 Years	Phase 1	10	2019/2/12
Cirrhosis	NCT01877759	A Clinical Study to Evaluate the Safety and Efficacy of Mesenchymal Stem Cells in Liver Cirrhosis	Unknown status	IV/Hepatic artery	Auto/Allo	BMMSCs/WJUCMSCs	25 Years to 65 Years	Phase 1|Phase 2	20	2013/6/14
Liver failure	NCT01218464	Safety and Efficacy of Human Mesenchymal Stem Cells for Treatment of Liver Failure	Unknown status	IV	Allo	WJUCMSCs	18 Years to 70 Years	Phase 1|Phase 2	70	2010/10/11
Liver failure	NCT03629015	Safety Study of Stemchymal® in Acute Liver Failure	Not yet recruiting	IV	Allo	AdMSCs	20 Years to 70 Years	Phase 1	20	2018/8/14
Liver failure	NCT03668171	Mesenchymal Stem Cell Transplantation for Acute-on-chronic Liver Failure	Recruiting	IV	UNS	UNS	18 Years to 70 Years	Not Applicable	200	2018/9/12
Liver failure	NCT03860155	Allogeneic ABCB5-positive Stem Cells for Treatment of Acute-on-Chronic Liver Failure	Recruiting	IV	Allo	Skin-MSCs	20 Years to 75 Years	Phase 1|Phase 2	18	2019/3/1
Liver failure	NCT03863002	Safety and Efficacy of Mesenchymal Stem Cell Transplantation for Acute-on-Chronic Liver Failure	Not yet recruiting	IV	UNS	UNS	16 Years to 60 Years	Phase 1|Phase 2	45	2019/3/5
Hepatitis/Cirrhosis	NCT01728727	Safety and Efficacy of Human Umbilical Cord Derived Mesenchymal Stem Cells for Treatment of HBV-related Liver Cirrhosis	Unknown status	Hepatic artery	Allo	WJUCMSCs	18 Years to 65 Years	Phase 1|Phase 2	240	2012/11/20

Hepatitis/Cirrhosis	NCT03826433	Clinical Study of Human Umbilical Cord Mesenchymal Stem Cells (19#iSCLife®-LC)in the Treatment of Decompensated Hepatitis b Cirrhosis	Recruiting	IV	Allo	WJUCMSCs	18 Years to 60 Years	Phase 1	20	2019/2/1	
Hepatitis/Cirrhosis	NCT04357600	Umbilical Cord Mesenchymal Stem Cell for Liver Cirrhosis Patient Caused by Hepatitis B	Recruiting	IV	Allo	WJUCMSCs	18 Years to 65 Years	Phase 1|Phase 2	12	2020/4/22	
Hepatitis/Cirrhosis	NCT02705742	Mesenchymal Stem Cells Transplantation for Liver Cirrhosis Due to HCV Hepatitis	Unknown status	IV	Auto	AdMSCs	18 Years to 70 Years	Phase 1|Phase 2	5	2016/3/10	
Hepatitis/Liver failure	NCT00956891	Therapeutic Effects of Liver Failure Patients Caused by Chronic Hepatitis B After Autologous MSCs Transplantation	Completed	UNS	Auto	BMMSCs	15 Years to 70 Years	Not Applicable	158	2009/8/11	
Hepatitis/Liver failure	NCT01221454	Allogenic Bone Marrow Stem Cell Transplantation in Liver Failure	Unknown status	IV	Allo	BMMSCS	16 Years to 65 Years	Phase 2	60	2010/10/15	
Hepatitis/Liver failure	NCT01322906	Allogeneic Bone Marrow Mesenchymal Stem Cells Transplantation in Patients With Liver Failure Caused by Hepatitis B Virus (HBV)	Unknown status	IV	Allo	BMMSCs	16 Years to 65 Years	Phase 2	120	2011/3/25	
Hepatitis/Liver failure	NCT01724398	Umbilical Cord Mesenchymal Stem Cells Transplantation Combined With Plasma Exchange for Patients With Liver Failure	Unknown status	IV	Allo	WJUCMSCs	16 Years to 65 Years	Phase 1|Phase 2	120	2012/11/9	Xu 2019[159]
Hepatitis/Liver failure	NCT02812121	UC-MSC Infusion for HBV-Related Acute-on-Chronic Liver Failure	Unknown status	IV	Allo	UCBMSCs	18 Years to 65 Years	Phase 2	261	2016/6/24	
Hepatitis/Liver failure	NCT01844063	Safety and Efficacy of Diverse Mesenchymal Stem Cells Transplantation for Liver Failure	Unknown status	IV	Allo	BMMSCs/WJUCMSCs	18 Years to 65 Years	Phase 1|Phase 2	210	2013/5/1	

Table S6: Clinical trials for non-immune diseases using MSC-derived products

Condition	NCT Number	Title	Status	Delivery	Auto/Allo?	Source MSCs	Age	Phase	Enrollment	First Posted
Hypoxic-Ischemic Encephalopathy	NCT02854579	Neural Progenitor Cell and Paracrine Factors to Treat Hypoxic Ischemic Encephalopathy	Unknown status	Intrathecal	UNS	UNS-MSC-CM	up to 14 Days	Not Applicable	120	2016/8/3
Diabetic foot ulcers	NCT02943486	Mesenchymal Stromal Cell Derivatives in the Treatment of Chronic Diabetic Foot Ulcers Type 1 and 2	Unknown status	Intradermic	UNS	UNS-MSCs/MSC-CM	40 Years to 80 Years	Phase 1|Phase 2	51	2016/10/24
Acute ischemic stroke	NCT03384433	Allogenic Mesenchymal Stem Cell Derived Exosome in Patients With Acute Ischemic Stroke	Completed	IV	Allo	UNS-MSC-Exo	40 Years to 80 Years	Phase 1|Phase 2	5	2017/12/27

Macular holes	NCT03437759	MSC-Exos Promote Healing of MHs	Recruiting	Into vitreous cavity	Allo	WJUCMSC-Exo	up to 80 Years	Early Phase 1	44	2018/2/19
Pancreatic cancer	NCT03608631	iExosomes in Treating Participants With Metastatic Pancreas Cancer With KrasG12D Mutation	Not yet recruiting	IV	UNS	UNS-MSC-Exo	18 Years and older	Phase 1	28	2018/8/1
Androgenic alopecia	NCT03676400	Hair Growth Efficacy and Safety of NGF-574H in Adult With Androgenic Alopecia	Completed	On hair	Allo	WJUCMSC-CM	18 Years to 60 Years	Not Applicable	84	2018/9/18
Residual burn wounds	NCT04235296	Mesenchymal Stem Cell Conditioned Medium-derived Pleiotropic Factor in Treating Residual Burn Wound	Recruiting	On the wound	UNS	UNS-MSC-PF	6 Years to 60 Years	Phase 1	30	2020/1/21
Chronic wounds	NCT04235868	Mesenchymal Stem Cell-derived Pleiotropic Factor in Treating Non-healing Wounds	Recruiting	On the wound	UNS	UNS-MSC-CM	18 Years to 75 Years	Phase 1	30	2020/1/22
Cerebral palsy	NCT04314687	Stem Cell and Conditioned Medium for Cerebral Palsy	Recruiting	Intrathecal	Allo	WJUCMSC-CM	6 Months to 3 Years	Phase 1|Phase 2	78	2020/3/19
Retinitis pigmentosa	NCT04315025	Safety Issues of Peribulbar Injection of UC-MSC in Patients With Retinitis Pigmentosa	Completed	Peribulbar	Allo	WJUCMSC-CM	18 Years to 65 Years	Phase 1|Phase 2	18	2020/3/19
Keloid scars	NCT04326959	Implantation of Mesenchymal Stem Cell, Conditioned Medium, or Triamcinolone Acetonide for Keloid	Not yet recruiting	Intralesion	Allo	WJUCMSC-CM	18 Years to 55 Years	Phase 1|Phase 2	24	2020/3/30
Multiple organ failure	NCT04356300	Exosome of Mesenchymal Stem Cells for Multiple Organ Dysfuntion Syndrome After Surgical Repaire of Acute Type A Aortic Dissection	Not yet recruiting	IV	Allo	WJUCMSC-Exo	20 Years to 80 Years	Not Applicable	60	2020/4/22
Alzheimer disease	NCT04388982	the Safety and the Efficacy Evaluation of Allogenic Adipose MSC-Exos in Patients With Alzheimer's Disease	Not yet recruiting	Nasal dripping	Allo	AdMSC-Exo	50 Years and older	Phase 1|Phase 2	9	2020/5/15
Nasal trauma	NCT04536233	Effects of MSCs Derived Pleiotropic Factors on Wound Healing in Endonasal Surgeries	Not yet recruiting	On the wound	UNS	UNS-MSC-PF	18 Years to 60 Years	Phase 1	50	2020/9/2
Chronic wounds	NCT04538885	Mesenchymal Stem Cell-derived Pleiotropic Factor in Treating Poorly Healed Wounds of Postoperative Incision	Not yet recruiting	On the wound	UNS	UNS-MSC-PF	18 Years to 75 Years	Phase 1	50	2020/9/4
Amyotrophic lateral sclerosis	NCT04681118	Expanded Access Protocol: Repeated Administration of Nurown (Autologous MSC-NTF Cells) for the Treatment of ALS	Available	Intrathecal	Auto	BMMSC-NF	18 Years to 63 Years	Not Applicable		2020/12/23
